# Synthesis and Cytotoxicity of Complex Betulinic Acid Amides

**DOI:** 10.1021/acsomega.6c00184

**Published:** 2026-05-19

**Authors:** Martina Wimmerová, Pavlína Kyjaková, David Šaman, Zdeněk Wimmer

**Affiliations:** † Department of Chemistry of Natural Compounds, University of Chemistry and Technology in Prague, Technická 5, 16028 Prague 6, Czech Republic; ‡ Institute of Organic Chemistry and Biochemistry, Czech Academy of Sciences, Flemingovo náměstí 2, 16610 Prague 6, Czech Republic

## Abstract

The main objective of this investigation was focused on the structure modification of betulinic acid to design novel cytotoxic compounds. The cytotoxicity of the amides of betulinic acid with several diamines was compared with that of the compounds bearing an additional amide bond formed by means of 4-methoxybenzoic acid. It was prepared by a green biotechnological approach from *trans*-anethole isolated from anise (*Pimpinella anisum* L.) seeds. Cisplatin and doxorubicin, the pharmacologically used anticancer agents, were used as the positive references. One of the target compounds (**9**) showed cytotoxicity in HeLa (IC_50_ = 5.4 ± 0.8 μM; SI > 9.3) and MCF7 (IC_50_ = 7.0 ± 1.1 μM; SI > 7.2) cancer cell lines while being nontoxic in normal human foreskin fibroblasts (IC_50_ > 50 μM), and it showed a better cytotoxicity profile than structurally related compounds **8** and **10**. The aromatic substituent and the C(3)-OAc group in **9** were also advantageous for the cytotoxicity profile of **9** in comparison with its intermediate compound **8**. Several intermediates and target compounds showed a better cytotoxicity profile than cisplatin.

## Graphical abstract

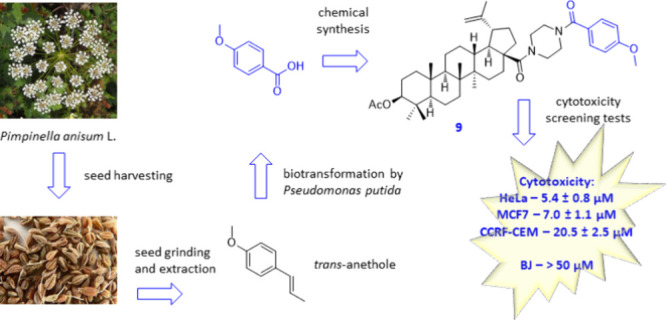

## 1 Introduction

In this investigation, betulinic acid, (3β)-3-hydroxylup-20(29)-en-28-oic acid (**1**; Scheme 1), a pentacyclic triterpenoid of the lupane family, was targeted. Many derivatives of betulinic acid have already proven their biological and even their pharmacological potential.^1^ The basic information on betulinic acid has already been given in our recent review papers on this topic.^1,2^ The current investigation should answer a question, if cytotoxicity of betulinic acid amides may increase due to benefiting from the additional structure modification by 4-methoxybenzoic acid, the product obtained by the biotransformation of *trans*-anethole, i.e., one of the products extracted from anise (*Pimpinella anisum* L.; Apiaceae) seeds. This strategy of designing novel cytotoxic agents was based on a similar finding made recently with the amides of moronic acid, morolic acid, oleanolic acid, and ursolic acid and with the oxime derivatives of betulonic acid and platanic acid.^3–6^

**Scheme 1:**
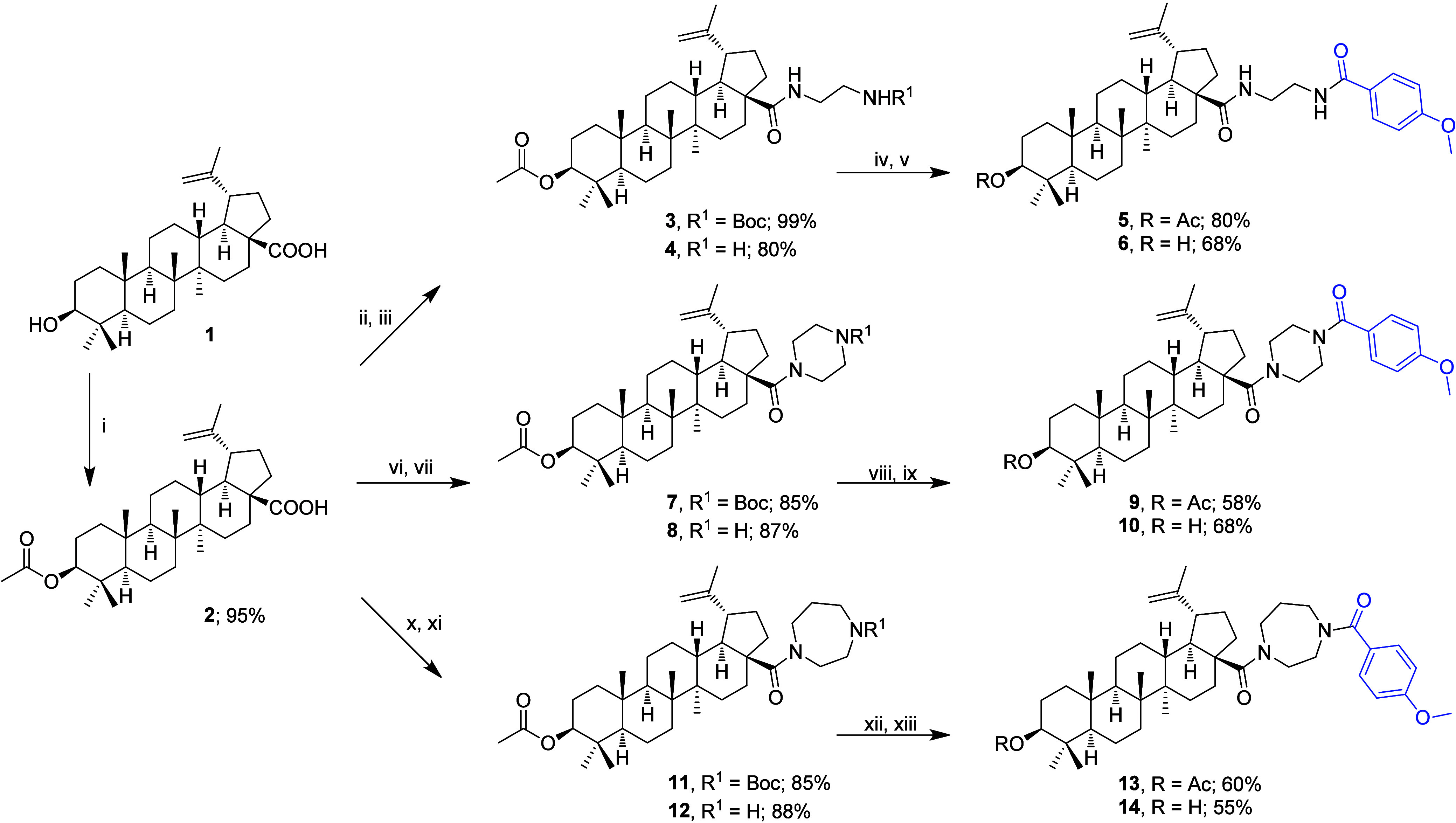
Synthetic Procedure^a^

a Legend: i: Ac_2_O, DMAP, DIPEA, THF; ii: (a) oxalyl chloride, DCM, (b) *N*-Boc-ethylenediamine, DIPEA, DCM; iii: HCl (g), 1,4-dioxane; iv: 4-methoxybenzoic acid, HOBt, NMM, EDC·HCl, DCM; v: LiOH·H_2_O, methanol; vi: (a) oxalyl chloride, DCM, (b) *N*-Boc-piperazine, DIPEA, DCM; vii: HCl (g), 1,4-dioxane; viii: 4-methoxybenzoic acid, HOBt, NMM, EDC·HCl, DCM; ix: LiOH·H_2_O, methanol; x: (a) oxalyl chloride, DCM, (b) *N*-Boc-homopiperazine hydrochloride, DIPEA, DCM; xi: HCl (g), 1,4-dioxane; xii: 4-methoxybenzoic acid, HOBt, NMM, EDC·HCl, DCM; xiii: LiOH·H_2_O, methanol.

In this research, the investigation has focused on plant triterpenoids and on their derivatives bearing various structures to develop novel antimicrobial, antiviral, and/or cytotoxic agents.^1,2^ Generally, the attention has often been focused on plant products to design a potential way of their derivatization. Plant triterpenoids were decorated by novel structural motifs to obtain new compounds displaying biological and/or supramolecular characteristics.^3–5^ The amide group present in the potentially biologically important compounds structurally based on plant triterpenoids seems to be an advantageous functional group for getting compounds with the required characteristics.^3–5^

In this work, the primary structure modification of betulinic acid was planned to be performed using several simple polyamines, i.e., ethylenediamine (1,2-diaminoethane), piperazine, and homopiperazine (1,4-diazepane). These simple polyamines had been used in the construction of different pharmaceutically important agents many times by different authors.^7–14^ We have used them for they bear two amine groups enabling subsequent construction of the amide bonds among different substituents. Even if the advantage of introducing the amide bonds into the triterpenoid-based compounds displaying cytotoxicity, antimicrobial activity, or antiviral activity has already been mentioned in the review papers,^1,2^ two series of the amide derivatives of betulinic acid should be pointed out. A series of 2-, 3-, and 4-picolyl amides of betulinic acid was published in 2018, showing clearly the enhancing effect of the prepared compounds on their cytotoxicity in comparison with the parent triterpenoid.^15^ The most successful compound of that series showed its IC_50_ = 0.5 ± 0.1 μM and a high therapeutic index (TI = 100) in the human malignant melanoma cell line (G-361), while it was inactive in normal human fibroblasts. A detailed investigation that included the flow cytometry analysis and studies of the activities of caspase-3 and caspase-7 resulted in a finding that this amide compound increased the proportion of cancer cells (G-361) in the G_0_/G_1_ phase and decreased their proportion in the concomitant phases, mainly G_2_/M. Apoptotic markers were detected, proving the antiproliferative effect of the studied compound. The experiments resulted in showing a degradation of caspase-7 into the cleaved fragments and the final apoptotic cascade.^15^ The enhancing effect of the amide derivatives of betulinic acid for antimicrobial activity and cytotoxicity was also found during the investigation of the cystamine amides of betulinic acid.^16^ Two compounds of the prepared series showed their selectivity for the human T-lymphoblastic leukemia cancer cells (CCRF-CEM) combined with the cytotoxicity in CCRF-CEM, showing IC_50_ = 3.0 ± 0.7 μM (TI = 20) and IC_50_ = 4.0 ± 1.9 μM (TI = 10), respectively. In turn, other compounds of this series displayed no cytotoxicity and showed high antimicrobial activity toward *Streptococcus mutans*, *Actinomyces odontolycus*, *Clostridium perfrigens*, and *Bacillus cereus*.^16^ Other papers published by the research team, which have already been mentioned above, dealt with either the more complex amide derivatives of different triterpenoids or with other series of triterpenoid-based compounds.^3–6^ However, a general conclusion resulting from this broad investigation proved that a beneficial effect of all these structure modifications of the triterpenoid molecules by introducing the new amide bonds into the target molecules on the biological activity existed and was observed.

Therefore, when we looked for an additional structure modifier of the amides of betulinic acid with ethylenediamine, piperazine, and homopiperazine, all of them bearing two amino groups, and which one could be used for the subsequent substitution of the free amino group, attention was focused on 4-methoxybenzoic acid. The importance of this versatile organic compound has been increasing across various practical applications, ranging from food research and industry and fragrance formulations up to applications in specific pharmaceutical compounds, namely, therapeutic agents.^17^ Even if different synthetic procedures may be applied in the synthesis of 4-methoxybenzoic acid, a biotransformation of *trans*-anethole has been preferred for the subsequent applications of 4-methoxybenzoic acid in food, cosmetic, and pharmaceutical areas.^17^ This finding directly connected the current project with another research activity of the team, focused on the investigation of anise (*Pimpinella anisum* L.; Apiaceae) seeds produced out of the Mediterranean area, where this herbaceous plant is usually grown.^18,19^ The anise plant grows in central Europe (Czech Republic; 50 °15′34″N and 14 °31′04″E) in the area having a specifically mild and relatively dry climate. Because *trans*-anethole was identified as the main component of the anise seed extract (cf. below in the text), we have included its microorganism-mediated biotransformation into 4-methoxybenzoic acid and used 4-methoxybenzoic acid in the construction of the target compounds displaying cytotoxicity.

The objectives of the present investigation have been focused on several partial tasks: (a) the extraction of the soft powdered anise seeds by two independent methods; (b) GC–MS analysis of the extracts resulting in a qualitative and quantitative identification of the extracted plant products; (c) selection of the convenient model product extracted, *trans*-anethole, based on the GC–MS analysis of the extracts; (d) microorganism-mediated conversion of *trans*-anethole into 4-methoxybenzoic acid for a subsequent structure modification of betulinic acid; (e) preparation of the amide derivatives of betulinic acid by a chemical synthesis using simple polyamines and 4-methoxybenzoic acid as the structure modifiers; (f) cytotoxicity screening tests of the prepared compounds in several cancer cell lines; and (g) evaluation of the cytotoxicity effects of the investigated compounds.

## 2 Experimental Section

### 2.1 Extraction of the Anise Seeds by Maceration

The soft powdered anise seeds (10 g) were put into the Erlenmeyer flask, covered with the solvent (ethanol or hexane; 50 mL), closed, and periodically shaken for 2 days. The supernatant was decanted, and the soft powder was covered with a fresh solvent (ethanol or hexane; 50 mL), closed, and periodically shaken for 5 days. The supernatant was decanted, and both parts of the supernatant were collected and filtered through a sintered glass funnel; the solvent was evaporated under reduced pressure, and the residue was stored in a refrigerator at 4 °C. This procedure resulted in obtaining three different extracts: (A) maceration of the soft powdered anise seeds with ethanol (1.0 g); (B) maceration of the soft powdered anise seeds with hexane, while ethanol was used for washing the solid phase during workup of the extract (1.2 g); and (C) maceration of the soft powdered anise seeds with hexane (1.4 g). The extractions were subjected to the GC–MS analysis. The results are summarized in Table 1.

**Table 1:** Relative Content of *trans*-Anethole (**PA1**), 4-Methoxybenzaldehyde (**PA2**), Estragole (**PA3**), and l-Fenchone (**PA4**) Based on the GC/MS Analysis of the Anise Seed Extracts (A)–(F)

		extraction method,^a^ the content of the products **PA1**–**PA4** [%]				
product	retention time [min]	(A)	(B)	(C)	(D)	(F)
**PA1**	11.79	90.90	79.34	77.92	86.99	82.78
**PA2**	11.16	0.95	1.02	1.06	0.76	1.34
**PA3**	10.16	2.10	4.03	4.80	2.87	3.45
**PA4**	8.15	6.06	15.62	16.23	9.38	12.44

a The Soxhlet-based water extract (E) of the anise seeds contained no relevant product.

### 2.2 Extraction of the Anise Seeds Using the Soxhlet Apparatus

The Soxhlet matrix was filled with soft powdered anise seeds (10 g); the solvent (ethanol or water; 175 mL) was put into the distillation flask, and heated to the boiling point of the solvent for 6 h. The extract was concentrated under reduced pressure to get the solvent-free residue that was stored in a refrigerator at 4 °C. This procedure resulted in three different extracts: (D) the extraction of the soft powdered anise seeds with ethanol (1.1 g); (E) the extraction of the soft powdered anise seeds with water (1.0 g); and (F) the extraction of the soft powdered anise seeds with ethanol under the inert argon atmosphere (1.3 g). The extractions were subjected to the GC–MS analysis. The results are summarized in Table 1.

### 2.3 Analytical and Synthetic Procedures

#### 2.3.1 General Analytical Methods Used

The NMR measurements were performed on a Bruker AVANCE II 600 MHz spectrometer equipped with a 5 mm TCI cryoprobe in a 5 mm tube in different solvents. The ^1^H NMR and ^13^C NMR spectra were recorded at 600.13 and 150.90 MHz (AVANCE II 600 MHz) in CDCl_3_ or DMSO-*d*_6_ using tetramethylsilane (δ = 0.0 – CDCl_3_) or signal of solvent (δ = 2.50 or 39.50 for ^1^H/^13^C – DMSO-*d*_6_) as internal references. The ^1^H NMR data are presented in the following order: chemical shift (δ) expressed in ppm, multiplicity (s, singlet; d, doublet; t, triplet; q, quartet; m, multiplet), number of protons, and coupling constants in Hertz. For unambiguous assignment of both ^1^H and ^13^C signals 2D NMR ^1^H,^13^C gHSQC and gHMBC spectra were measured using standard parameter sets and pulse programs delivered by the producer of the spectrometer. Infrared (IR) spectra were measured with a Nicolet iS5 FT-IR spectrometer. Mass spectra were measured with a Waters ZMD mass spectrometer in the positive ESI mode (coin voltage, CV = 10 to 20 eV). The gas chromatography/mass spectrometry (GC/MS) analysis was performed on a ThermoFisher Scientific Trace 1310 GC instrument equipped with an ISQ LT mass-selective detector (Waltham, MA, USA). Thin-layer chromatography was carried out on silica gel plates (Merck 60F_254_), and the visualization was performed by both UV detection and spraying with the methanolic solution of phosphomolybdic acid (5%) followed by heating the plate. For column chromatography, silica gel 60 (0.063–0.200 mm) from Merck was used. *Pseudomonas putida*, strain DBM 3177, was obtained from the collection of microorganisms of the University of Chemistry and Technology in Prague, Czech Republic. All chemicals and solvents were purchased from regular commercial sources in the analytical grade, and the solvents were purified by the general methods before use. Betulinic acid was purchased from Dr. Jan Šarek – Betulinines (www.betulinines.com). Figure 1 should assist with an unambiguous assignment of the NMR signals of the carbon atoms, the numbers of which are single primed ones. The assignment of the ^1^H and ^13^C NMR signals of the prepared compounds, the scanned ^1^H and ^13^C NMR spectra of the prepared compounds (Figures S1–S26), as well as the calculated ^1^H and ^13^C NMR spectra (ChemBioDraw Ultra, version 12.0) are presented in the Supporting Information, Part I.

**Figure 1:**
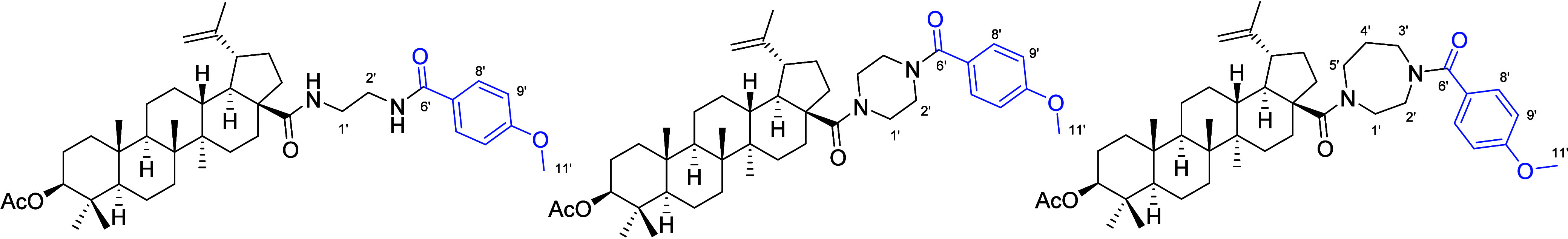
Numbering of the Single Primed Carbon Atoms. The numbers should assist in the assignment of the NMR signal. They do not correspond to the numbering of the carbon atoms according to the nomenclature rules.

#### 2.3.2 GC/MS Analysis of the Extracts and the Isolation of *trans*-Anethole

The extracts were analyzed using GC coupled with MS detection (70 eV ionization voltage, source temperature 200 °C, transferline heated to 260 °C) equipped with a nonpolar capillary column ZB-5MS (30 m, internal diameter 0.25 mm, film thickness 0.25 μm; Phenomenex, Torrance, CA, USA). The temperature program was 50 °C, gradually increased to 320 °C at 8 °C·min^–1^, and held for 20 min. Helium was used as the carrier gas at a flow of 1.2 mL·min^–1^. The split/splitless injector was heated to 200 °C, and the samples were injected in the splitless mode with a purge time of 1 min. The identity of the compounds was confirmed by comparing the EI mass spectra and the retention indices using the NIST (National Institute of Standards and Technology, Gaithersburg, MD, USA) library database.

Finally, *trans*-anethole (**PA1**) was isolated from the extraction mixtures by column chromatography on silica gel using various ratios of petroleum ether/diethyl ether mixtures as the mobile phases. Its identification was made by the above-described GC/MS analysis.

#### 2.3.3 A Biotransformation of *trans*-Anethole (**PA1**) to 4-Methoxybenzoic Acid (**PA1-MBA**)

This experiment was made according to the procedure described in the literature.^20–22^ The fermentation medium was composed of glucose (10 g), peptone (5 g), yeast extract (2.5 g), K_2_HPO_4_·3H_2_O (1.2 g), NaCl (0.5 g), MgSO_4_·7H_2_O (1.0 g), and (NH_4_)_2_SO_4_ (0.5 g), FeSO_4_ (0.01 g), and distilled water (1000 mL). *Pseudomonas putida*, strain DBM 3177, was inoculated in 250 mL Erlenmeyer flasks containing a fresh fermentation medium (100 mL) and *trans*-anethole (5 g·L^–1^). The microorganism was cultured at 30 °C and 200 min^–1^ for 48 h. Then, the culture liquid was extracted with chloroform, the extract was dried over sodium sulfate, the solid was filtered off, and the solvent was evaporated under a reduced pressure. The residue was subjected to column chromatography on silica gel using chloroform as the mobile phase, affording 4-methoxybenzoic acid (**PA1-MBA**) in a 25% yield. The product was identified by GC/MS using the NIST database.

#### 2.3.4 (3β)-3-(Acetyloxy)­lup-20­(29)-en-28-oic Acid (**2**)

Acetic anhydride (450 μL; 4.74 mmol), DMAP (55 mg; 0.45 mmol), and diisopropyl ethyl amine (DIPEA, 1 mL; 5.74 mmol) were added to a solution of **1** (1.53 g; 3.35 mmol) in THF (10 mL) under the argon atmosphere. The reaction mixture was stirred and heated under reflux for 3 h. The mixture was cooled to r.t., quenched with water, stirred for 1 h, and extracted with chloroform (6 × 15 mL). The extract was dried over sodium sulfate, the solid was filtered off, and the solvent was evaporated under reduced pressure, affording the crude product that was purified by column chromatography on silica gel, using chloroform as the mobile phase, affording pure **2** in a 95% yield. MS: *m*/*z* = 497.3 [M–H]^−^ (ESI^–^). For C_32_H_50_O_4_ (498.74) calcd. C 77.06, H 10.10, found C 77.10, H 10.08.

#### 2.3.5 *tert*-Butyl-4-[(3β)-3-(acetyloxy)-28-oxolup-20­(29)-en-28-yl]­ethylenediamine-1-carboxylate (**3**)

Oxalyl chloride (3.51 mL; 7.02 mmol) was added to a stirred solution of **2** (500 mg; 1.002 mmol) in dichloromethane (DCM) (10 mL) under the argon atmosphere, and the stirring was continued for 3 h. Then, the mixture was evaporated under a reduced pressure, leaving a residue that was dissolved in fresh DCM under stirring. *N*-Boc-ethylenediamine (190 μL; 1.20 mmol) and DIPEA (454 μL; 2.61 mmol) were added under the argon atmosphere, and the reaction mixture was stirred overnight. Then, the solvent was evaporated under the reduced pressure, and the residue was purified by column chromatography on silica gel using chloroform/methanol mixture (100:0 to 125:1) as the mobile phase, affording product **3** in a 99% yield. IR (cm^–1^): 3359 (CO-NH), 2940 (CH_2_), 2868 (CH_3_), 1715 (C=O), 1639 (C=O), 1506 (NH), 1453 (CH_2_), 1364 (CH_3_), 1242 (C–O–C), 1167 (C–N–C), 978 (CH). MS: *m*/*z* = 641.3 [M + H]^+^, 663.3 [M + Na]^+^, 1299.5 [2M+NH_4_]^+^ (ESI^+^). For C_39_H_64_N_2_O_5_ (640.94) calcd. C 73.08, H 10.06, N 4.37, found C 73.10, H 10.05, N 4.39.

#### 2.3.6 (3β)-28-Oxo-28-(ethylenediamin-1-yl)­lup-20­(29)-en-3-yl Acetate (**4**)

Hydrogen chloride (g) dissolved in 1,4-dioxane (1.06 mL) was added to **3** (610 mg; 0.95 mmol) in a sealed reaction vessel. The mixture was left to react at 35 °C overnight. The volatiles were allowed to evaporate under a reduced pressure, and the crude residue was purified on silica gel using a chloroform/methanol mixture (30:1 to 5:1) as the mobile phase, affording **4** in an 80% yield. IR (cm^–1^): 3350 (CO-NH, NH_2_), 2939 (CH_2_), 2850 (CH_3_), 1730 (C=O), 1365 (CH_3_), 1242 (C–O–C), 1025 (NH_2_), 977 (CH), 751 (NH_2_). MS: *m*/*z* = 541.3 [M + H]^+^ (ESI^+^). For C_34_H_56_N_2_O_3_ (540.82), calcd. C, 75.51, H, 10.44, N, 5.18, found: C, 75.54, H, 10.41, N, 5.21.

#### 2.3.7 (3β)-28-Oxo-28-[*N*-(4-methoxybenzoyl)­ethylenediamin-1-yl]­lup-20­(29)-en-3-yl Acetate (**5**)

1-Hydroxybenzotriazole (HOBt; 15 mg; 0.11 mmol), *N*-methylmorpholine (165 μL; 1.5 mmol), and EDC·HCl (148 mg; 0.75 mmol) were added to a solution of **4** (270 mg; 0.5 mmol) in DCM (27 mL). The reaction was left to stand overnight and then extracted with chloroform (7 × 10 mL). The extract was dried over sodium sulfate, the solid was filtered off, and the solvent was evaporated under a reduced pressure. The residue was purified by column chromatography on silica gel using chloroform/methanol (70:1 to 50:1) as the mobile phase, affording **5** in an 80% yield. IR (cm^–1^): 3350 (CO-NH), 2938 (CH_2_), 2874 (CH_3_), 1727 (C=O), 1635 (C=O) 1503 (NH), 1245 (C–O–C), 1024 (C–O–C), 977 (CH). MS: *m*/*z* = 675.3 [M + H]^+^ (ESI^+^), 673.4 [M–H]^+^ (ESI^–^). For C_42_H_62_N_2_O_5_ (674.95) calcd. C 74.74, H 9.26, N 4.15, found C 74.72, H 9.28, N 4.18.

#### 2.3.8 (3β)-3-Hydroxy-28-[*N*-(4-methoxybenzoyl)­ethylenediamin-1-yl]­lup-20­(29)-en-28-one (**6**)

LiOH·H_2_O (78 mg; 1.85 mmol) was added to a solution of **5** (250 mg; 0.37 mmol) in methanol (15 mL) under stirring. The reaction was heated under reflux for 5 h. The solvent was evaporated under a reduced pressure, and the residue was extracted with chloroform (7 × 10 mL). The extract was dried over sodium sulfate, the solid was filtered off, and the solvent was evaporated under a reduced pressure. The residue was purified by column chromatography on silica gel using chloroform/methanol (125:1 to 70:1) as the mobile phase, affording **6** in a 68% yield. IR (cm^–1^): 3347 (CO-NH, OH), 3070 (CH_2_), 2936 (CH_2_), 2863 (CH_3_), 1666 (CH), 1635 (C=O), 1605 (C=C), 1503 (NH), 1245 (C–O–C), 1030 (C–O–C). MS: *m*/*z* = 633.4 [M + H]^+^, (ESI^+^), 631.0 [M–H]^+^, (ESI^–^). For C_40_H_60_N_2_O_4_ (632.92) calcd. C 75.91, H 9.56, N 4.43, found C 75.94, H 9.54, N 4.45.

#### 2.3.9 *tert*-Butyl 4-[(3β)-3-(Acetyloxy)-28-oxolup-20(29)-en-28-yl]­piperazin-1-carboxylate (**7**)

Compound **7** was prepared in an 85% yield by applying the procedure described in [Sec sec2.3.4] and using **2** and *N*-Boc-piperazine as the key reagents. IR (cm^–1^): 3065 (CH_2_), 2939 (CH_2_), 2867 (CH_3_), 1727 (C=O), 1697 (C=C), 1634 (C=O), 1391 (CH_2_), 1364 (CH_3_), 1239 (C–O–C), 1164 (C–N–C), 1024 (C–O–C), 997 (CH), 978 (CH), 751 (C–N–C). MS: *m*/*z* = 667.3 [M + H]^+^ (ESI^+^). For C_41_H_66_N_2_O_5_ (666.97), calcd. Calcd for C_16_H_15_N_2_O_2_S: C, 73.83, H, 9.97, N, 4.20; found: C, 73.80, H, 9.99, N, 4.22.

#### 2.3.10 (3β)-28-Oxo-28-(piperazin-1-yl)­lup-20­(29)-en-3-yl Acetate (**8**)

Compound **8** was prepared in an 87% yield from **7** by applying the procedure described in [Sec sec2.3.6]. IR (cm^–1^): 3400 (CO-NH, NH_2_), 2969 (CH_2_), 2900 (CH_3_), 1720 (C=O), 1630 (C=O), 1470 (CH_2_), 1405 (CO-H), 1392 (CH_2_), 1249 (C–O–C), 1075 (C–C) 1065 (C–O–C), 1045 (C–O–C), 1027 (C–O–C). MS: *m*/*z* = 567.6 [M + H]^+^ (ESI^+^). For C_36_H_58_N_2_O_3_ (566.44), calcd. Calcd for C_22_H_22_N_2_O_2_S: C, 76.28, H, 10.31, N, 4.94; found: C, 76.25, H, 10.30, N, 4.96.

#### 2.3.11 (3β)-28-Oxo-28-[*N*-(4-methoxybenzoyl)­piperazin-1-yl]­lup-20­(29)-en-3-yl Acetate (**9**)

Compound **9** was prepared in a 58% yield from **8** by applying the procedure described in [Sec sec2.3.7]. IR (cm^–1^): 2930 (CH_2_), 2850 (CH_3_), 1725 (C=O), 1635 (C=O), 1628 (C=C), 1430 (CH_3_), 1244 (C–O–C), 1171 (C–O–C), 1000 (C–N–C), 747 (NH_2_). MS: *m*/*z* = 701.3 [M + H]^+^, 722.8 [M + Na]^+^ (ESI^+^). For C_44_H_64_N_2_O_5_ (700.99), calcd. Calcd for C_26_H_28_N_2_O_2_: C, 75.39, H, 9.20, N, 4.00; found: C, 75.42, H, 9.18, N, 4.03.

#### 2.3.12 (3β)-3-Hydroxy-28-[*N*-(4-methoxybenzoyl)­piperazin-1-yl]­lup-20­(29)-en-28-one (**10**)

Compound **10** was prepared in a 68% yield from **9** by applying the procedure described in [Sec sec2.3.8]. IR (cm^–1^): 3490 (CO-NH, OH), 2936 (CH_2_), 2850 (CH_3_), 1627 (C=C), 1454 (CH_2_), 1427 (CH_3_), 1407 (C–O–H), 1250 (C–O–C), 1175 (C–O–C), 1002 (C–O–C). MS: *m*/*z* = 659.4 [M + H]^+^ (ESI^+^). For C_42_H_62_N_2_O_4_ (658.95) calcd: C 76.55, H 9.48, N 4.25; found: C 76.52, H 9.49, N 4.22.

#### 2.3.13 *tert*-Butyl 4-[(3β)-3-(Acetyloxy)-28-oxolup-20(29)-en-28-yl]­homopiperazin-1-carboxylate (**11**)

Compound **11** was prepared in an 85% yield by applying the procedure described in [Sec sec2.3.5] and using **2** and *N*-Boc-homopiperazine hydrochloride as the key reagents. IR (cm^–1^): 3450 (CO-NH, OH), 2936 (CH_2_), 2850 (CH_3_), 1613 (C=C), 1471 (CH_2_), 1426 (CH_3_), 1400 (C–O–H), 1244 (C–O–C), 1173 (C–N–C), 1030 (C–O–C), 841 (C–N–C). MS: *m*/*z* = 680.5 [M + H]^+^, 704.2 [M + Na]^+^ (ESI^+^). For C_42_H_68_N_2_O_5_ (681.00), calcd: C 74.07, H 10.06, N 4.11; found: C 74.10, H 10.04, N 4.14.

#### 2.3.14 (3β)-28-Oxo-28-(homopiperazin-1-yl)­lup-20­(29)-en-3-yl Acetate (**12**)

Compound **12** was prepared in an 88% yield from **11** by applying the procedure described in [Sec sec2.3.6]. IR (cm^–1^): 3450 (CO-NH, OH), 2936 (CH_2_), 2850 (CH_3_), 1613 (C=C), 1471 (CH_2_), 1426 (CH_3_), 1400 (C–O–H), 1244 (C–O–C), 1173 (C–N–C), 1030 (C–O–C), 841 (C–N–C). MS: *m*/*z* = 581.5 [M + H]^+^ (ESI^+^). For C_37_H_60_N_2_O_3_ (580.88), calcd. C, 76.50, H, 10.41, N, 4.82, found: C, 76.53, H, 10.39, N, 4.84.

#### 2.3.15 (3β)-28-Oxo-28-[*N*-(4-methoxybenzoyl)­homopiperazin-1-yl]­lup-20­(29)-en-3-yl Acetate (**13**)

Compound **13** was prepared in a 60% yield from **12** by applying the procedure described in [Sec sec2.3.7]. IR (cm^–1^): 3450 (CO-NH, OH), 2936 (CH_2_), 2850 (CH_3_), 1613 (C=C), 1471 (CH_2_), 1426 (CH_3_), 1400 (C–O–H), 1244 (C–O–C), 1173 (C–N–C), 1030 (C–O–C), 841 (C–N–C). MS: *m*/*z* = 714.5 [M + H]^+^ (ESI^+^). For C_45_H_66_N_2_O_5_ (715.02), calcd. Calcd for C_28_H_30_N_2_O_2_S: C, 75.59, H, 9.30, N, 3.92; found: C, 75.61, H, 9.29, N, 3.94.

#### 2.3.16 (3β)-3-Hydroxy-28-[*N*-(4-methoxybenzoyl)­homopiperazin-1-yl]­lup-20­(29)-en-28-one (**14**)

Compound **14** was prepared in a 55% yield from **13** by applying the procedure described in [Sec sec2.3.8]. IR (cm^–1^): 3450 (CO-NH, OH), 2936 (CH_2_), 2850 (CH_3_), 1613 (C=C), 1471 (CH_2_), 1426 (CH_3_), 1400 (C–O–H), 1244 (C–O–C), 1173 (C–N–C), 1030 (C–O–C), 841 (C–N–C). MS: *m*/*z* = 673.1 [M + H]^+^, 695.2 [M + Na]^+^ (ESI^+^). For C_43_H_64_N_2_O_4_ (672.98), calcd. C, 76.74, H, 9.59, N, 4.16, found: C, 76.71, H, 9.61, N, 4.14.

#### 2.3.17 Cell Cultures

The screening cell lines, T-lymphoblastic leukemia CCRF-CEM, breast carcinoma MCF7, cervical carcinoma HeLa, and human foreskin fibroblasts BJ, were obtained from the American Type Culture Collection (Manassas, VA, USA). The procedure for cell culturing has already been described.^15,16,23^

#### 2.3.18 Cytotoxicity Screening Tests

Description of the experimental procedure used in the cytotoxicity test has already been published.^15,16,23^ The IC_50_ values obtained with the compounds **4**–**6**, **8**–**10,** and **12**–**14** are shown in Table 2.

**Table 2:** Cytotoxicity Values (IC_50_ ± SD [μM]) of **4**–**6**, **8**–**10,** and **12**–**14** in Three Cancer Cell Lines and in the Normal Human Fibroblasts (BJ) after 72 h

	IC_50_ ± SD [μM], 72 h			
compound	MCF7^a^	CCRF-CEM^b^	HeLa^c^	BJ^d^
**4**	4.3 ± 0.1	4.5 ± 0.2	3.9 ± 0.0	8.7 ± 2.8
**5**	11.2 ± 0.2	30.0 ± 2.1	13.7 ± 0.2	>50
**6**	>50	>50	>50	>50
**8**	5.1 ± 0.8	2.8 ± 1.1	3.8 ± 0.1	9.9 ± 2.4
**9**	7.0 ± 1.1^e^	20.5 ± 2.5^f^	5.4 ± 0.8^g^	>50
**10**	5.7 ± 0.9	13.3 ± 2.5	7.3 ± 0.6	4.1 ± 0.6
**12**	4.9 ± 0.2	3.5 ± 0.6	4.1 ± 0.3	10.6 ± 2.5
**13**	12.1 ± 1.4	32.5 ± 2.6	15.7 ± 0.9	>50
**14**	>50	>50	>50	>50
**CDDP** ^h^	7.7 ± 1.7	0.8 ± 0.1	11.4 ± 3.8	6.9 ± 0.9
doxorubicin	0.273 ± 0.019	n. t.^i^	0.868 ± 0.054	0.278 ± 0.036

a Breast adenocarcinoma.

b T-Lymphoblastic leukemia.

c Cervical cancer.

d Normal foreskin fibroblast.

e SI (selectivity index) > 7.2.

f SI > 2.5.

g SI > 9.3.

h Cisplatin, *cis*-diaminedichloroplatinum­(II).

i This was not tested in our research team.

## 3 Results and Discussion

### 3.1 Synthetic Procedure

The synthetic procedure for the preparation of the planned betulinic acid derivatives started by converting betulinic acid (**1**) into its C(3)-acetate **2** to protect the C(3)–OH group in the subsequent reaction steps [Scheme 1; path (i)]. To prepare the required products **5** and **6**, the triterpenoid derivative **2** was initially converted to its acyl chloride using oxalyl chloride as the reagent, and the crude intermediate was immediately used for the preparation of **3** using *N*-Boc-ethylenediamine as the key reagent and adding DIPEA as the base [path (ii)]. To liberate the protected amino group, the Boc-protecting group in **3** was removed by hydrogen chloride (gas) dissolved in 1,4-dioxane, yielding **4** [path (iii)]. Compound **4** was subjected to formation of a new amide bond with 4-methoxybenzoic acid, finally using 1-hydroxybenzotriazole (HOBt), *N*-methylmorpholine (NMM), and 1-ethyl-3-(3-(dimethylamino)­propyl)­carbodiimide hydrochloride (EDC·HCl) as the reaction components, dissolved in DCM, finally yielding **5** [path (iv)]. However, to develop this reaction step with an as high as possible yield of **5**, two other ways were tested; how to perform this transformation before the above-described procedure was selected: (a) Using 1-propanephosphonic acid anhydride (T3P) as a condensation agent of **4** with 4-methoxybenzoic acid in dry pyridine and (b) converting 4-methoxybenzoic acid to its acyl chloride using oxalyl chloride and then reacting it with **4** using DIPEA as a base and a condensation agent. However, neither of these two alternative methods, (a) or (b), resulted in obtaining **5** in acceptable yields. After solving the successful procedure described above [path (iv)], the acetyl protecting group in **5** was removed by LiOH·H_2_O in methanol to get **6** [path (v)]. Other reactions shown in Scheme 1 are analogous: To prepare products **9** and **10**, triterpenoid derivative **2** was again converted to its acyl chloride, and subsequently, using the crude acyl chloride in a reaction with *N*-Boc-piperazine, **7** was prepared [path (vi)]. Paths (vii) to (ix) are analogous to paths (iii) to (v) described above. To prepare products **13** and **14**, the synthesis started from **2**, which was first converted to its acyl chloride, used in its crude form in the following reaction with *N*-Boc-homopiperazine hydrochloride, affording intermediate **11** (path x). Paths (xi) to (xiii) are analogous to paths (iii) to (v) described above. The demonstrated synthetic pathways were used in different modifications during the investigation performed in our team. Therefore, three most recent papers may be presented as examples of different general synthetic methods applied in this investigation and used in the above-described synthetic procedure.^3,6,24^

### 3.2 Anise Seeds, Extraction Methods, GC–MS Analysis of the Extracts, and Isolation of Its Main Components

To prepare 4-methoxybenzoic acid from *trans*-anethole by the biotransformation process, anise seeds were grown and harvested to extract the required plant products therefrom. Anise seeds were left to dry at room temperature for a month and then powdered using a standard laboratory grinder, leaving a soft powder that was stored in a closed small container under an argon atmosphere at room temperature. In this investigation, only two simple and independent extraction methods have been selected: maceration and the Soxhlet-type extraction of the powder material. The extractions were made by using different solvents. The extracts were subjected to GC–MS analysis to identify their components (Table 1).

The GC–MS analysis of the extracts resulted in the identification of six different plant products. An international database was used to assist with the identification of compounds. The results of the GC–MS analysis of the extracts (A) to (F) are summarized in Table 1. During the analysis, it was found that the extract (E) obtained through the extraction of the powdered anise seeds with water contained no relevant extracted plant product, giving clear evidence that the plant products from the anise seeds are low-polar compounds.

The results revealed that both the extraction methods used were comparable based on the content of the identified compounds: *trans*-anethole (1-methoxy-4-[(1*E*)-prop-1-en-1-yl]­benzene; **PA1**), *p*-anisaldehyde (4-methoxybenzaldehyde; **PA2**), estragole (1-methoxy-4-(prop-2-en-1-yl)­benzene; **PA3**), l-fenchone [(1*R*,4*S*)-1,3,3-trimethylbicyclo­[2.2.1]­heptan-2-one; **PA4**], hexadecanoic (palmitic) acid, and (9*Z*)-octadecenoic (oleic) acid. The most important anise seed products **PA1**–**PA4** are shown in Figure 2; the structures of the palmitic acid and oleic acid are not given. The obtained results of the GC analysis are presented in the Supporting Information. The content of the main compound in these extractions, *trans*-anethole (**PA1**), varied only slightly; nevertheless, certain advantage of using ethanol as the extraction solvent, both in the maceration and the Soxhlet-based procedures, seems to be evident.

**Figure 2:**
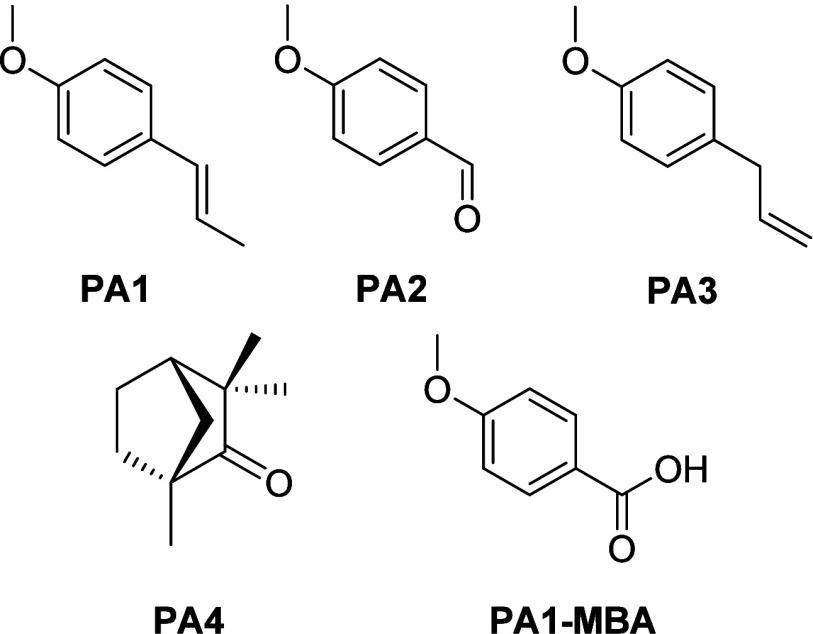
Structures of *trans*-anethole (**PA1**), 4-methoxybenzaldehyde (**PA2**), estragole (**PA3**), and l-fenchone (**PA4**) isolated from the anise seeds. 4-Methoxybenzoic acid (**PA1-MBA**) is the product of a biotransformation of *trans*-anethole (**PA1**) by *Pseudomonas putida* DBM 3177.

Finally, *trans*-anethole (**PA1**), the main component of the extracts [methods (A)–(D) and (F); Table 1] of the soft powdered anise seeds, was isolated from the extraction mixtures by column chromatography on silica gel using petroleum ether/diethyl ether mixture as the mobile phase.

### 3.3 Microorganism-Mediated Transformation of *trans*-Anethole to 4-Methoxybenzoic Acid

As the main component of the extracts of the anise seeds, *trans*-anethole (**PA1**) was found in the quantities between 78 and 91% in the extracts based on the solvent and the conditions used (Table 1). Due to the structure of *trans*-anethole (**PA1**), a very limited possibility exists to use it directly for the derivatization of betulinic acid, and therefore, a decision was made to convert *trans*-anethole (**PA1**) into 4-methoxybenzoic acid, a compound receiving increasing importance as stated above. It represents a product of the biotransformation of *trans*-anethole and, potentially, a product of a transformation of 4-methoxybenzaldehyde (**PA2**) and estragole (**PA3**). Because the contents of 4-methoxybenzaldehyde (**PA2**) and estragole (**PA3**) in the extracts of the anise seeds were relatively low, attention was focused on *trans*-anethole (**PA1**). l-Fenchone (**PA4**) was not considered for use in this investigation due to its low content in the extracts from the anise seeds and due to a need for an additional structural transformation before its application in the synthesis. In the past, a conversion of *trans*-anethole (**PA1**) to 4-methoxybenzoic acid (**PA1-MBA**; Figure 2) was made by the biotechnological approach using *Burkholderia* sp. as the key microorganism capable of performing this type of biotransformation.^20,21^ Because *Burkholderia* sp. was not available in the collection of microorganisms of the University of Chemistry and Technology in Prague, a similar microorganism, classified as *Pseudomonas putida*, strain DBM 3177, was employed in the investigation and used for the biotransformation of *trans*-anethole (**PA1**) to 4-methoxybenzoic acid (**PA1-MBA**; Figure 2).^22^ The required product, 4-methoxybenzoic acid (**PA1-MBA**), was obtained in a 25% yield, which was a comparable yield to that obtained by using *Burkholderia* sp. for the biotransformation process in the past.

### 3.4 Cytotoxicity and the Structure–Activity Relationships

Based on the results achieved, several compounds showing cytotoxicity, namely in the MCF7 and HeLa cancer cell lines, were selected. Compounds **4**, **8,** and **12** that are considered as the synthetic precursors of the compounds **5**, **6**, **9**, **10**, **13,** and **14** showed cytotoxicity in all three cancer cell lines; however, they were also toxic in human fibroblasts. In turn, the target compounds **6**, **10,** and **14** were either inactive (**6** and **14**), or cytotoxic in all tested cell lines (**10**), both cancer and nonmalignant ones. However, the C(3)-acetates **5**, **9,** and **13**, i.e., the esterified target compounds, showed cytotoxicity in all three cancer cell lines, and they were not toxic to the human fibroblasts. This result revealed that the additional amide bond present not only in molecule **9** but also in molecules **5** and **13** compared to their precursors **4**, **8,** and **12** is beneficial for the cytotoxicity profile of these compounds bearing a molecular motif of 4-methoxybenzoic acid (**PA1-MBA**) that was derived by a biotransformation process from *trans*-anethole (**PA1**) extracted from the seeds of *P. anisum* L. (Table 2). The piperazine unit in **9** and **10** seems to be responsible for their cytotoxicity in comparison with **5**, **6**, **13,** and **14**, bearing the ethylenediamine unit or the homopiperazine unit in their molecules, respectively. The in silico modeling of the molecule conformations in **5**, **6**, **9**, **10**, **13,** and **14** resulted in no clear answer, because the conformations of **5** and **9** are very similar, while that of the homopiperazine-unit-bearing compound **13** differ from **5** and **9** (cf. Figures S27–S29 in the Supporting Information, Part II). Therefore, the reason for the found cytotoxicity of compound **9**, bearing the piperazine unit, in comparison with the other related compounds of this series seems to be more complex (Table 2). Nevertheless, **9** was evaluated as the most successful structure of this series concerning the cytotoxicity effect showing the selectivity index values SI > 9.3 (HeLa) and SI > 7.2 (MCF7). Generally, the compounds showing cytotoxicity influenced the cell cycle and caused apoptosis that is initiated by the caspase-3 and caspase-7 cascade activation.^15,16^ A positive reference compound, *cis*-diaminedichloroplatinum­(II), (**CDDP**, cisplatin), a pharmacologically used agent for treating cancers, was used in the cytotoxicity screening tests. Table 2 also shows that the pharmacological profile of **CDDP** is comparable with that of **4**, **8,** and **12**, while **5**, **9,** and **13** show it better than **CDDP**. Because the **CDDP** molecule contains the platinum ion and the species can be characterized as a coordination complex of platinum, doxorubicin, an organic molecule-based chemotherapy medication for treating cancer, was used as the latter positive reference. However, the cytotoxicity of doxorubicin is much higher than that of **CDDP** and the studied compounds (Table 2).

### 3.5 Structure Elucidation Tools

Supporting Information, Part I (Figures S1–S26) shows the ^1^H and ^13^C NMR spectra of the prepared compounds. The list of peaks is described in the Experimental section, paragraphs [Sec sec2.3.4]–[Sec sec2.3.16], with the experimental details on the synthesis of the compounds. In addition, to assist in the structure elucidation of the prepared compounds in more detail, the 2D ^1^H,^13^C gHSGC and gHMBC NMR spectra are presented in the Supporting Information (Part III, Figures S30–S37), accompanied by a brief description of the strategy of using the 2D ^1^H,^13^C gHSGC and gHMBC NMR spectra in the structure elucidation.

## 4 Conclusions

In this study, six natural products were obtained from the softly powdered anise seeds by two different ways of extraction. All plant products were identified by GC/MS, and four of them (**PA1**–**PA4**) were considered as important plant products. The major one of them, *trans*-anethole (**PA1**), was structurally modified by a biotransformation process by *P. putida* strain DBM 3177 into 4-methoxybenzoic acid (**PA1-MBA**) that was used in designing a novel series of potentially cytotoxic compounds (Scheme 1). The yield of the product (**PA1-MBA**) of the biotransformation reaction was about 25%. Compounds **4**–**6**, **8**–**10,** and **12**–**14** were prepared and subjected to the cytotoxicity screening test using three different cancer cell lines (HeLa, MCF7, and CCRF-CEM). Normal human fibroblasts (BJ) were used as the reference cells for comparing the selectivity of the effect of the studied compounds in treating cancer cell lines. The most successful compound of this series (**9**) showed an optimum level of cytotoxicity in the cancer cell lines and a low or no toxicity in the normal fibroblasts. It was followed by compounds **5** and **13** that were less cytotoxic than **9** but showed similar cytotoxicity profiles. Compound **9** was found as the agent displaying the highest cytotoxicity in this series of compounds, i.e., in HeLa (IC_50_ = 5.4 ± 0.8 μM; SI > 9.3) and MCF7 (IC_50_ = 7.0 ± 1.1 μM; SI > 7.2) but less cytotoxic in CCRF-CEM (IC_50_ = 20.5 ± 2.5 μM; SI > 2.5), and noncytotoxic in the normal human fibroblasts (BJ; IC_50_ > 50 μM). This investigation clearly revealed the potential of anise in producing a natural product, *trans*-anethole (**PA1**), capable of serving as a convenient natural and sustainable product with the application in medicinal chemistry and in the production of potential agents for cancer treatment.

## Supplementary Material



## Notes

Experimental procedures, product characterization, molecular modeling, copies of the ^1^H and ^13^C NMR spectra, and results of the GC/MS analysis (PDF)

## Abbreviations

BJ: normal foreskin fibroblast
CCRF-CEM: T-lymphoblastic leukemia
DCM: dichloromethane
DIPEA: ethyl diisopropyl amine
EDC·HCl: 1-ethyl-3-(3-(dimethylamino)­propyl)­carbodiimide hydrochloride
HeLa: cervical cancer
HOBt: hydroxybenzotriazole
MCF7: breast adenocarcinoma
NMM: *N*-methylmorpholine
T3P: 1-propanephosphonic acid anhydride
