# Supplementary material for: Synthesis and Cytotoxicity of Complex Betulinic Acid Amides
Source: ACS Omega. 2026 May 19;11(21):30955–62. doi: 10.1021/acsomega.6c00184 (PMC13234881; doi:10.1021/acsomega.6c00184)

SUPPORTING INFORMATION

Synthesis and Cytotoxicity of Complex Betulinic Acid Amides

Martina Wimmerová,[a #] Pavlína Kyjaková,[b #] David Šaman,[b] and Zdeněk Wimmer [a,*]

[a] University of Chemistry and Technology in Prague, Department of Chemistry of Natural Compounds, Technická 5, CZ-16028 Prague 6, Czech Republic;

[b] Institute of Organic Chemistry and Biochemistry, Czech Academy of Sciences, Flemingovo náměstí 2, CZ-16610 Prague 6, Czech Republic.

Content:

Experimental part

Part I. NMR spectra (Figures S1– S26) and 3D structures of the selected compounds (Figures S27– S29)

Part II. Results of the GC×GC analysis of the extracts of anise (*Pimpinella anisum* L.) seeds

Part III. 2D ^{1}H,^{13}C gHSQC and gHMBC NMR spectra of the selected key compounds (Figures S30–S37)

Part I. NMR spectra, assignment of the signals in the ^{1}H and ^{13}C NMR spectra

(3β)-3-(Acetyloxy)lup-20(29)-en-28-oic acid (**2**)

^{1}H-NMR (600.13 MHz, $CDCl_3$): δ [ppm] 0.83 (s, 3H, 23-CH_3), 0.84 (s, 3H, 24-CH_3), 0.85 (d, 3H, J=0.6 Hz, 25-CH_3), 0.93 (s, 3H, 26-CH_3), 0.97 (d, 3H, J=0.6 Hz, 27-CH_3), 1.69 (dd, 3H, J_1=0.6 Hz, J_2=1.4 Hz, 30-CH_3), 2.04 (s, 3H, **CH_3**CO), 3.00 (dt, 1H, J_1=4.8 Hz, J_2=10.8 Hz, J_3=10.8 Hz, 19-CH), 4.46-4.49 (m, 1H, 3-CH), 4.60-4.62 (m, 1H, H-29), 4.73-4.75 (m, 1H, H-29). ^{13}C-NMR (150.90 MHz, $CDCl_3$): δ [ppm] 14.66 (q, C27), 16.05 (q, C26), 16.17 (q, C25), 16.46 (q, C24), 18.17(t, C6), 19.35 (t, C11), 20.87 (q, C30), 21.29 (q, **CH_3**CO), 23.70 (t, C2), 25.46 (t, C12), 27.95 (q, C23), 29.70 (t, C21), 30.58 (t, C15), 31.01 (d, C3), 32.16 (t, C16), 37.04 (t, C22), 37.14 (s, C10), 37.26 (t, C7), 37.81 (s, C4), 38.40 (d, C13), 38.44 (t, C1), 40.72 (s, C8), 42.44 (s, C14), 46.95 (d, C18), 49.30 (d, C19), 50.42 (d, C9), 55.44 (d, C5), 56.40 (s, C17), 109.73 (t, C29), 150.36 (s, C20), 171.03 (s, CH_3**CO**), 181.86 (s, C28).

tert-Butyl-4-[(3β)-3-(acetyloxy)-28-oxolup-20(29)-en-28-yl]ethylenediamine-1-carboxylate (**3**)

^{1}H NMR (600.13 MHz, $CDCl_3$): δ [ppm] 0.78 (1H, dd, J_1=2.2 Hz, J_2=11.4 Hz, H-5), 0.82 (3H, s, H24), 0.84 (3H, s, H-23), 0.84 (3H, s, H-25), 0.93 (3H, s, H-26), 0.95 (3H, s, H-27), 1.13 (2H, dt, J_1=3.4 Hz, J_2=3.4 Hz, J_3=13.3 Hz, H-21), 1.43 (9H, s, H-7'), 1.55 (1H, t, J=11.3 Hz, H-18), 1.66 (2H, dt, J_1=3.4 Hz, J_2=3.4 Hz, J_3=13.0 Hz, H-7), 1.68 (3H, dd, J_1=0.7 Hz, J_2=1.4 Hz, H-30), 1.74 (2H, bdd, J_1=7.8 Hz, J_2=12.0 Hz, H-1), 1.91-1.98 (4H, m, H-15+H-16), 2.04 (3H, s, **CH_3**CO), 2.45 (1H, ddd, J_1=3.7 Hz, J_2=11.6 Hz, J_3=13.0 Hz, H-13), 3.12 (1H, dt, J_1=4.4 Hz, J_2=11.1 Hz, J_3=11.1 Hz, H-19), 3.22-3.38 (2H, m, H-1'), 3.22-3.38 (2H, m, H-2'), 4.46 (1H, dd, J_1=6.2 Hz, J_2=10.4 Hz, H3), 4.59 (1H, dq, J_1=1.4 Hz, J_2=1.4 Hz, J_3=1.4 Hz, J_4=2.4 Hz, H-29), 4.74 (1H, dq, J_1=0.7 Hz, J_2=0.7 Hz, J_3=0.7 Hz, J_4=2.4 Hz, H-29). ^{13}C NMR (150.90 MHz, $CDCl_3$): δ [ppm] 14.60 (q, C27), 16.20 (q, C24), 16.20 (q, C25), 16.50 (q, C26), 18.20

(t, C6), 19.40 (q, C30), 20.90 (t, C11), 21.30 (q, **CH_3**CO), 23.70 (t, C2), 25.60 (t, C12), 27.90 (q, C23), 28.40 (q, C7'), 29.50 (t, C21), 30.90 (t, C15), 33.60 (t, C16), 34.30 (t, C22), 37.10 (s, C10), 37.70 (d, C13), 37.80 (s, C4), 38.40 (t, C1), 38.40 (t, C7), 40.40 (t, C1'), 40.70 (s, C8), 40.70 (t, C2'), 42.40 (s, C14), 46.70 (d, C19), 50.10 (d, C18), 50.50 (d, C9), 55.40 (s, C17), 55.60 (d, C5), 79.60 (s, C6'), 80.90 (d, C3), 109.40 (t, C29), 151.00 (s, C20), 156.90 (s, C5'), 171.00 (s, CH_3**CO**), 177.20 (s, C28).

(3β)-28-Oxo-28-(ethylenediamin-1-yl)lup-20(29)-en-3-yl acetate (**4**)

^{1}H NMR (600.13 MHz, $CDCl_3$): δ [ppm] 0.78 (1H, dd, J_1=1.9 Hz, J_2=11.5 Hz, H-5), 0.83 (3H, s, H-23), 0.84 (3H, s, H-24), 0.84 (3H, s, H-25), 0.92 (3H, s, H-26), 0.96 (3H, s, H-27), 1.68 (3H, dd, J_1=0.7 Hz, J_2=1.3 Hz, H-30), 2.04 (3H, s, **CH_3**CO), 2.10 (1H, dt, J_1=3.2 Hz, J_2=3.2 Hz, J_3=13.7 Hz, H-13), 2.43 (2H, ddd, J_1=3.7 Hz, J_2=11.7 Hz, J_3=12.8 Hz, H-16), 3.01 (2H, t, J=5.7 Hz, H-2'), 3.07 (2H, dt, J_1=4.5 Hz, J_2=11.0 Hz, J_3=11.0 Hz, H-19), 3.43 (1H, dq, J_1=5.6 Hz, J_2=5.6 Hz, J_3=5.6 Hz, J_4=14.0 Hz, H-1'), 3.51 (1H, dq, J_1=5.6 Hz, J_2=5.6 Hz, J_3=5.6 Hz, J_4=14.0 Hz, H-2'), 4.47 (2H, dd, J_1=5.7 Hz, J_2=10.7 Hz, H-3), 4.59 (1H, dq, J_1=1.3 Hz, J_2=1.3 Hz, J_3=1.3 Hz, J_4=2.4 Hz, H-29), 4.72 (1H, dq, J_1=0.7 Hz, J_2=0.7 Hz, J_3=0.7 Hz, J_4=2.4 Hz, H-29), 6.84 (2H, t, J=5.6 Hz, NH). ^{13}C NMR (150.90 MHz, $CDCl_3$): δ [ppm] 14.58 (q, C27), 16.14 (q, C24), 16.19 (q, C25), 16.46 (q, C26), 18.17 (t, C6), 19.37 (q, C30), 20.96 (t, C11), 21.26 (q, **CH_3**CO), 23.67 (t, C2), 25.54 (t, C12), 27.92 (q, C23), 29.46 (t, C21), 30.91 (t, C15), 33.45 (t, C16), 34.32 (t, C22), 37.10 (d, C13), 37.70 (s, C10), 37.76 (t, C7), 38.33 (s, C4), 38.38 (t, C1), 39.26 (t, C1'), 40.75 (s, C8), 41.00 (t, C2'), 42.42 (s, C14), 46.79 (t, C19), 50.15 (d, C18), 50.51 (d, C9), 55.44 (s, C17), 55.77 (d, C5), 80.90 (d, C3), 109.51 (t, C29), 150.72 (s, C20), 170.98 (s, CH_3**CO**), 177.61 (s, C28).

(3β)-28-Oxo-28-[*N*-(4-methoxybenzoyl)ethylenediamin-1-yl]lup-20(29)-en-3-yl acetate (**5**)

^{1}H NMR (600.13 MHz, $CDCl_3$): δ [ppm] 0.67 (3H, s, H-24), 0.71 (3H, d, *J*=0.4 Hz, H-25), 0.72 (1H, dd, J_1=2.0 Hz, J_2=11.5 Hz, H-5), 0.80 (3H, s, H-26), 0.81 (3H, s, H-23), 0.91 (3H, d, *J*=0.7 Hz, H-27), 1.14 (2H, dt, J_1=3.2 Hz, J_2=3.2 Hz, J_3=13.2 Hz, H-21), 1.57 (1H, t, *J*=11.7 Hz, H-18), 1.66 (3H, dd, J_1=0.7 Hz, J_2=1.4 Hz, H-30), 1.72 (2H, ddd, J_1=1.0 Hz, J_2=8.0 Hz, J_3=12.5 Hz, H1), 1.85 (2H, ddd, J_1=8.0 Hz, J_2=11.1 Hz, J_3=13.5 Hz, H-15), 1.96 (2H, dt, J_1=3.5 Hz, J_2=3.5 Hz, J_3=13.8 Hz, H-16), 2.04 (3H, s, **CH_3**CO), 2.30 (1H, ddd, J_1=3.7 Hz, J_2=11.6 Hz, J_3=12.4 Hz, H-13), 3.13 (1H, dt, J_1=4.6 Hz, J_2=11.1 Hz, J_3=11.1 Hz, H-19), 3.47-3.52 (2H, m, H-1'), 3.57-3.62 (2H, m, H-2'), 3.83 (3H, s, H-11'), 4.44 (1H, dd, J_1=5.2 Hz, J_2=11.0 Hz, H-3), 4.59 (1H, dq, J_1=1.4 Hz, J_2=1.4 Hz, J_3=1.4 Hz, J_4=2.4 Hz, H-29), 4.73 (1H, dq, J_1=0.7 Hz, J_2=0.7 Hz, J_3=0.7 Hz, J_4=2.4 Hz, H-29), 6.90-6.93 (2H, m, H-9'), 7.80-7.82 (2H, m, H-8'). ^{13}C NMR (150.90 MHz, $CDCl_3$): δ [ppm] 14.50 (q, C27), 15.90 (q, C24), 16.00 (q, C25), 16.40 (q, C26), 18.10 (t, C6), 19.50 (q, C30), 20.80 (t, C11), 21.27 (q, **CH_3**CO), 23.70 (t, C2), 25.50 (t, C12), 27.90 (q, C23), 29.50 (t, C21), 30.80 (t, C15), 33.60 (t, C16), 34.10 (t, C22), 37.00 (s, C10), 37.70 (t, C7), 37.90 (d, C13), 38.30 (s, C4), 38.40 (t, C1), 39.40 (s, C8), 40.60 (t, C1'), 42.10 (t, C2'), 42.40 (s, C14), 46.90 (d, C19), 50.10 (d, C18), 50.40 (d, C9), 55.30 (q, C11'), 55.40 (d, C5), 55.80 (s, C17), 80.90 (d, C3), 109.40 (t, C29), 113.60 (d, C9'), 126.10 (s, C7'), 128.90 (d, C8'), 150.80 (s, C20), 162.50 (s, C10'), 167.60 (s, C6'), 170.96 (s, CH_3**CO**), 178.70 (s, C28).

(3β)-3-Hydroxy-28-[*N*-(4-methoxybenzoyl)ethylenediamin-1-yl]lup-20(29)-en-28-one (**6**)

^{1}H NMR (600.13 MHz, DMSO-d_6): δ [ppm] 0.58 (1H, dd, J_1=2.0 Hz, J_2=11.6 Hz, H-5), 0.63 (3H, s, H-25), 0.70 (3H, s, H-26), 0.85 (3H, s, H-27), 0.87 (3H, s, H-24), 0.89 (3H, s, H-23), 0.93 (2H, dt, J_1=3.1 Hz, J_2=3.1 Hz, J_3=13.2 Hz, H-21), 1.61 (3H, dd, J_1=0.7 Hz, J_2=1.4 Hz, H-30), 1.70 (2H, dt, J_1=3.6 Hz, J_2=3.6 Hz, J_3=13.5 Hz, H-1), 2.10 (2H, dt, J_1=3.4 Hz, J_2=3.4 Hz,

J_3=13.5 Hz, H-16), 2.47 (1H, ddd, J_1=3.7 Hz, J_2=11.6 Hz, J_3=13.0 Hz, H-13), 2.95 (1H, dd, J_1=5.1 Hz, J_2=10.2 Hz, H-3), 3.00 (1H, dt, J_1=4.6 Hz, J_2=10.9 Hz, J_3=10.9 Hz, H-19), 3.47-3.52 (2H, m, H-1'), 3.57-3.62 (2H, m, H-2'), 3.83 (3H, s, H-11'), 4.52 (1H, dq, J_1=1.4 Hz, J_2=1.4 Hz, J_3=1.4 Hz, J_4=2.6 Hz, H-29), 4.64 (1H, dq, J_1=0.7 Hz, J_2=0.7 Hz, J_3=0.7 Hz, J_4=2.6 Hz, H-29), 6.97-6.99 (2H, m, H-9'), 7.81-7.83 (2H, m, H-8'). ^{13}C NMR (150.90 MHz, DMSO-d_6): δ [ppm] 14.30 (q, C27), 15.00 (q, C24), 15.80 (q, C25), 15.90 (q, C26), 17.90 (t, C6), 19.00 (q, C30), 20.50 (t, C11), 25.20 (t, C12), 27.10 (t, C2), 28.10 (q, C23), 30.70 (t, C21), 32.40 (t, C15), 33.70 (t, C16), 35.10 (t, C22), 35.80 (t, C7), 36.70 (s, C10), 36.70 (d, C13), 38.20 (s, C4), 38.50 (t, C1), 40.20 (s, C8), 40.60 (t, C1'), 41.80 (s, C14), 42.10 (t, C2'), 46.20 (d, C19), 49.60 (d, C9), 50.00 (d, C18), 54.90 (d, C5), 54.90 (s, C17), 55.20 (q, C11'), 76.80 (d, C3), 109.20 (t, C29), 113.40 (d, C9'), 126.60 (s, C7'), 128.90 (d, C8'), 150.90 (s, C20), 162.30 (s, C10'), 167.80 (s, C6'), 176.10 (s, C28).

tert-Butyl 4-[(3β)-3-(acetyloxy)-28-oxolup-20(29)-en-28-yl]piperazin-1-carboxylate (**7**)

^{1}H NMR (600.13 MHz, $CDCl_3$): δ [ppm] 0.78 (1H, dd, J_1=2.1 Hz, J_2=11.2 Hz, H-5), 0.82 (3H, s, H-25), 0.83 (3H, s, H-26), 0.84 (3H, s, H-24), 0.92 (3H, s, H-23), 0.95 (3H, s, H-27), 1.15 (2H, dt, J_1=3.4 Hz, J_2=3.4 Hz, J_3=13.4 Hz, H-21), 1.46 (9H, s, H-7'), 1.68 (3H, dd, J_1=0.7 Hz, J_2=1.3 Hz, H-30), 1.80 (2H, m, H-15), 1.91 (2H, m, H-7), 2.03 (3H, s, **CH_3**CO), 2.07 (2H, dt, J_1=3.2 Hz, J_2=3.2 Hz, J_3=13.3 Hz, H-16), 2.85 (1H, ddd, J_1=3.5 Hz, J_2=11.4 Hz, J_3=13.1 Hz, H-13), 2.97 (1H, dt, J_1=4.2 Hz, J_2=11.2 Hz, J_3=11.2 Hz, H-19), 3.35-3.65 (4H, m, H-1'), 3.35-3.66 (4H, m, H-2'), 4.46 (1H, dd, J_1=5.6 Hz, J_2=10.7 Hz, H-3), 4.58 (1H, dq, J_1=1.3 Hz, J_2=1.3 Hz, J_3=1.3 Hz, J_4=2.4 Hz, H-29), 4.72 (1H, dq, J_1=0.7 Hz, J_2=0.7 Hz, J_3=0.7 Hz, J_4=2.4 Hz, H-29). ^{13}C NMR (150.90 MHz, $CDCl_3$): δ [ppm] 14.60 (q, C27), 16.10 (q, C24), 16.20 (q, C25), 16.50 (q, C26), 18.20 (t, C6), 19.60 (q, C30), 21.10 (t, C11), 21.30 (q, **CH_3**CO), 23.70 (t, C2), 25.60 (t, C12), 27.90 (q, C23), 28.40 (q, C7'), 29.80 (t, C21), 31.00 (d, C3), 31.30 (t,

C15), 32.60 (t, C16), 34.30 (t, C22), 36.00 (s, C10), 36.90 (t, C7), 37.10 (d, C13), 37.80 (s, C4), 38.40 (t, C1), 40.70 (s, C8), 41.90 (s, C14), 43.40 (t, C1'), 44.20 (t, C2'), 45.70 (d, C19), 50.70 (d, C9), 52.70 (d, C18), 54.60 (s, C17), 55.50 (d, C5), 80.20 (s, C6'), 109.30 (t, C29), 151.20 (s, C20), 154.70 (s, C5'), 171.00 (s, CH_3**CO**), 173.80 (s, C28).

(3β)-28-Oxo-28-(piperazin-1-yl)lup-20(29)-en-3-yl acetate (**8**)

^{1}H NMR (600.13 MHz, $CDCl_3$): δ [ppm] 0.78 (1H, dd, J_1=2.1 Hz, J_2=11.2 Hz, H-5), 0.82 (3H, s, H-25), 0.83 (3H, s, H-26), 0.84 (3H, s, H-24), 0.92 (3H, s, H-23), 0.95 (3H, s, H-27), 1.15 (2H, dt, J_1=3.4 Hz, J_2=3.4 Hz, J_3=13.4 Hz, H-21), 1.46 (9H, s, H-7'), 1.68 (3H, dd, J_1=0.7 Hz, J_2=1.3 Hz, H-30), 1.80 (2H, m, H-15), 1.91 (2H, m, H-7), 2.04 (3H, s, **CH_3**CO), 2.07 (2H, dt, J_1=3.2 Hz, J_2=3.2 Hz, J_3=13.3 Hz, H-16), 2.85 (1H, ddd, J_1=3.5 Hz, J_2=11.4 Hz, J_3=13.1 Hz, H-13), 2.97 (1H, dt, J_1=4.2 Hz, J_2=11.2 Hz, J_3=11.2 Hz, H-19), 3.35-3.65 (4H, m, H-1'), 3.35-3.66 (4H, m, H-2'), 4.46 (1H, dd, J_1=5.6 Hz, J_2=10.7 Hz, H-3), 4.58 (1H, dq, J_1=1.3 Hz, J_2=1.3 Hz, J_3=1.3 Hz, J_4=2.4 Hz, H-29), 4.72 (1H, dq, J_1=0.7 Hz, J_2=0.7 Hz, J_3=0.7 Hz, J_4=2.4 Hz, H-29). ^{13}C NMR (150.90 MHz, $CDCl_3$): δ [ppm] 14.60 (q, C27), 16.10 (q, C24), 16.20 (q, C25), 16.50 (q, C26), 18.20 (t, C6), 19.60 (q, C30), 21.10 (t, C11), 21.30 (q, **CH_3**CO), 23.70 (t, C2), 25.60 (t, C12), 27.90 (q, C23), 28.40 (q, C7'), 29.80 (t, C21), 31.00 (d, C3), 31.30 (t, C15), 32.60 (t, C16), 34.30 (t, C22), 36.00 (s, C10), 36.90 (t, C7), 37.10 (d, C13), 37.80 (s, C4), 38.40 (t, C1), 40.70 (s, C8), 41.90 (s, C14), 43.40 (t, C1'), 44.20 (t, C2'), 45.70 (d, C19), 50.70 (d, C9), 52.70 (d, C18), 54.60 (s, C17), 55.50 (d, C5), 80.20 (s, C6'), 109.30 (t, C29), 151.20 (s, C20), 154.70 (s, C5'), 171.00 (s, CH_3**CO**), 173.80 (s, C28).

(3β)-28-Oxo-28-[*N*-(4-methoxybenzoyl)piperazin-1-yl]lup-20(29)-en-3-yl acetate (**9**)

^{1}H NMR (600.13 MHz, $CDCl_3$): δ [ppm] 0.78 (1H, dd, J_1=2.3 Hz, J_2=11.3 Hz, H-5), 0.83 (3H, s, H-23), 0.83 (3H, s, H-25), 0.84 (3H, s, H-26), 0.93 (3H, s, H-24), 0.96 (3H, s, H-27), 1.16

(2H, dt, J_1=3.2 Hz, J_2=3.2 Hz, J_3=13.4 Hz, H-21), 1.57 (1H, t, J_1=11.3 Hz, J_2=11.3 Hz, H-18), 1.68 (3H, dd, J_1=0.7 Hz, J_2=1.3 Hz, H-30), 1.82-1.86 (2H, m, H-15), 1.91-1.95 (2H, m, H-7), 2.04 (s, **CH_3**CO), 2.08 (2H, bdt, J_1=3.2 Hz, J_2=3.2 Hz, J_3=13.2 Hz, H-16), 2.84 (1H, ddd, J_1=3.5 Hz, J_2=11.5 Hz, J_3=13.1 Hz, H-13), 2.97 (1H, dt, J_1=4.0 Hz, J_2=11.2 Hz, J_3=11.2 Hz, H-19), 3.55-3.70 (8H, m, H-1'+H-2'), 3.84 (3H, s, H-11'), 4.47 (1H, dd, J_1=5.9 Hz, J_2=9.3 Hz, H-3), 4.59 (1H, dq, J_1=1.3 Hz, J_2=1.3 Hz, J_3=1.3 Hz, J_4=2.3 Hz, H-29), 4.73 (1H, dq, J_1=0.7 Hz, J_2=0.7 Hz, J_3=0.7 Hz, J_4=2.3 Hz, H-29), 6.91-6.94 (2H, m, H-9'), 7.37-7.40 (2H, m, H-8'). ^{13}C NMR (150.90 MHz, $CDCl_3$): δ [ppm] 14.60 (q, C27), 16.10 (q, C24), 16.20 (q, C25), 16.50 (q, C26), 18.20 (t, C6), 19.60 (q, C30), 21.10 (t, C11), 21.26 (q, **CH_3**CO), 23.70 (t, C2), 25.60 (t, C12), 27.90 (q, C23), 29.80 (t, C21), 31.30 (t, C15), 32.60 (t, C16), 34.30 (t, C22), 36.00 (t, C7), 36.90 (d, C13), 37.10 (s, C10), 37.80 (s, C4), 38.40 (t, C1), 40.70 (s, C8), 41.90 (s, C14), 45.70 (d, C19), 50.70 (d, C9), 52.70 (d, C18), 54.70 (s, C17), 55.40 (q, C11'), 55.50 (d, C5), 80.90 (d, C3), 109.40 (t, C29), 113.80 (d, C9'), 127.30 (s, C7'), 129.20 (d, C8'), 151.10 (s, C20), 161.00 (s, C10'), 170.98 (s, CH_3**CO**), 170.70 (s, C6'), 173.90 (s, C28).

(3β)-3-Hydroxy-28-[*N*-(4-methoxybenzoyl)piperazin-1-yl]lup-20(29)-en-28-one (**10**)

^{1}H NMR (600.13 MHz, $CDCl_3$): δ [ppm] 0.68 (1H, dd, J_1=2.2 Hz, J_2=11.5 Hz, H-3), 0.75 (3H, s, H-25), 0.82 (3H, s, H-26), 0.90 (1H, dt, J_1=3.7 Hz, J_2=12.8 Hz, J_3=12.8 Hz, H-1), 0.93 (3H, s, H-24), 0.96 (3H, s, H-27), 0.97 (3H, s, H-23), 1.18 (2H, dt, J_1=3.3 Hz, J_2=3.3 Hz, J_3=13.4 Hz, H-21), 1.67 (1H, dt, J_1=3.5 Hz, J_2=3.5 Hz, J_3=13.0 Hz, H-1), 1.67 (3H, dd, J_1=0.7 Hz, J_2=1.4 Hz, H-30), 1.82 (2H, m, H-15), 1.92 (2H, m, H-7), 2.08 (2H, bdt, J_1=3.2 Hz, J_2=3.2 Hz, J_3=13.5 Hz, H-16), 2.85 (1H, ddd, J_1=3.6 Hz, J_2=11.4 Hz, J_3=13.0 Hz, H-13), 2.97 (1H, dt, J_1=4.3 Hz, J_2=11.4 Hz, J_3=11.4 Hz, H-19), 3.18 (1H, dd, J_1=4.8 Hz, J_2=11.5 Hz, H-5), 3.50-3.75 (8H, m, H-1'+H-2'), 3.84 (3H, s, H-11'), 4.58 (1H, dq, J_1=1.4 Hz, J_2=1.4 Hz, J_3=1.4 Hz, J_4=2.4 Hz, H-29), 4.73 (1H, dq, J_1=0.7 Hz, J_2=0.7 Hz, J_3=0.7 Hz, J_4=2.4 Hz, H-29), 6.90-6.94

(2H, m, H-9'), 7.38-7.41 (2H, m, H-8'). ^{13}C NMR (150.90 MHz, $CDCl_3$): δ [ppm] 14.70 (q, C27), 15.30 (q, C24), 16.10 (q, C25), 16.20 (q, C26), 18.30 (t, C6), 19.60 (q, C30), 21.10 (t, C11), 25.60 (t, C12), 27.40 (t, C2), 28.00 (q, C23), 29.80 (t, C21), 31.30 (t, C15), 32.60 (t, C16), 34.40 (t, C22), 36.00 (t, C7), 36.90 (s, C10), 37.20 (d, C13), 38.70 (s, C4), 38.90 (t, C1), 40.70 (s, C8), 41.90 (s, C14), 45.60 (d, C19), 50.80 (d, C9), 52.70 (d, C18), 54.70 (s, C17), 55.40 (d, C5), 55.40 (q, C11'), 79.00 (d, C3), 109.30 (t, C29), 113.80 (d, C9'), 127.30 (s, C7'), 129.20 (d, C8'), 151.10 (s, C20), 161.00 (s, C10'), 170.70 (s, C6'), 173.90 (s, C28).

tert-Butyl 4-[(3β)-3-(acetyloxy)-28-oxolup-20(29)-en-28-yl]homopiperazin-1-carboxylate (**11**)

^{1}H NMR (600.13 MHz, $CDCl_3$): δ [ppm] 0.68 (1H, bdd, J_1=1.8 Hz, J_2=11.2 Hz, H-5), 0.75 (3H, s, H-25), 0.82 (3H, s, H-26), 0.93 (3H, s, H-24), 0.96 (3H, s, H-23), 0.96 (3H, s, H-27), 1.16 (2H, bt, J=13.3 Hz, H-21), 1.67 (3H, bd, J=1.4 Hz, H-30), 2.04 (s, 3H, **CH_3**CO), 2.07 (2H, bs, H-16), 2.89 (1H, bt, J=12.5 Hz, H-13), 3.00 (1H, bt, J=11.5 Hz, H-19), 3.17 (1H, dd, J_1=4.8 Hz, J_2=11.5 Hz, H-3), 3.35-4.10 (2H, m, H-1'), 3.35-4.10 (2H, m, H-2'), 3.35-4.10 (2H, m, H-3'), 3.35-4.10 (2H, m, H-4'), 3.35-4.10 (2H, m, H-5'), 4.57 (1H, bs, H-29), 4.72 (1H, bs, H-29), ^{13}C NMR (150.90 MHz, $CDCl_3$): δ [ppm] 14.70 (q, C27), 15.30 (q, C24), 16.10 (q, C25), 16.20 (q, C26), 18.30 (t, C6), 19.60 (q, C30), 21.10 (t, C11), 21.29 (q, **CH_3**CO), 25.60 (t, C12), 27.20 (d, C13), 27.40 (t, C2), 28.00 (q, C23), 28.30 (t, C4'), 30.20 (t, C21), 31.40 (t, C15), 32.20 (t, C16), 34.40 (t, C22), 36.10 (t, C7), 37.00 (s, C10), 38.70 (s, C4), 38.80 (t, C1), 40.70 (s, C8), 42.00 (s, C14), 45.60 (t, C3'), 45.80 (d, C19), 47.70 (t, C5'), 48.10 (t, C1'), 50.80 (d, C9), 53.00 (d, C18), 55.10 (s, C17), 55.40 (d, C5), 79.00 (d, C3), 109.20 (t, C29), 151.40 (s, C20), 171.03 (CH_3**CO**), 174.90 (s, C28).

(3β)-28-Oxo-28-(homopiperazin-1-yl)lup-20(29)-en-3-yl acetate (**12**)

^{1}H NMR (600.13 MHz, $CDCl_3$): δ [ppm] 0.68 (1H, bdd, J_1=1.8 Hz, J_2=11.2 Hz, H-5), 0.75 (3H, s, H-25), 0.82 (3H, s, H-26), 0.93 (3H, s, H-24), 0.96 (3H, s, H-23), 0.96 (3H, s, H-27), 1.16 (2H, bt, *J*=13.3 Hz, H-21), 1.67 (3H, bd, *J*=1.4 Hz, H-30), 2.03 (s, 3H, C2'), 2.07 (2H, bs, H-16), 2.89 (1H, bt, *J*=12.5 Hz, H-13), 3.00 (1H, bt, *J*=11.5 Hz, H-19), 3.17 (1H, dd, J_1=4.8 Hz, J_2=11.5 Hz, H-3), 3.35-4.10 (2H, m, H-1'), 3.35-4.10 (2H, m, H-2'), 3.35-4.10 (2H, m, H-3'), 3.35-4.10 (2H, m, H-4'), 3.35-4.10 (2H, m, H-5'), 4.57 (1H, bs, H-29), 4.72 (1H, bs, H-29). ^{13}C NMR (150.90 MHz, $CDCl_3$): δ [ppm] 14.70 (q, C27), 15.30 (q, C24), 16.10 (q, C25), 16.20 (q, C26), 18.30 (t, C6), 19.60 (q, C30), 21.10 (t, C11), 21.29 (**CH_3**CO), 25.60 (t, C12), 27.20 (d, C13), 27.40 (t, C2), 28.00 (q, C23), 28.30 (t, C4'), 30.20 (t, C21), 31.40 (t, C15), 32.20 (t, C16), 34.40 (t, C22), 36.10 (t, C7), 37.00 (s, C10), 38.70 (s, C4), 38.80 (t, C1), 40.70 (s, C8), 42.00 (s, C14), 45.60 (t, C3'), 45.80 (d, C19), 47.70 (t, C5'), 48.10 (t, C1'), 50.80 (d, C9), 53.00 (d, C18), 55.10 (s, C17), 55.40 (d, C5), 79.00 (d, C3), 109.20 (t, C29), 151.40 (s, C20), 171.03 (CH_3**CO**), 174.90 (s, C28).

(3β)-28-Oxo-28-[*N*-(4-methoxybenzoyl)homopiperazin-1-yl]lup-20(29)-en-3-yl acetate (**13**)

^{1}H NMR (600.13 MHz, $CDCl_3$): δ [ppm] 0.68 (1H, bdd, J_1=1.8 Hz, J_2=11.2 Hz, H-5), 0.75 (3H, s, H-25), 0.82 (3H, s, H-26), 0.93 (3H, s, H-24), 0.96 (3H, s, H-23), 0.96 (3H, s, H-27), 1.16 (2H, bt, *J*=13.3 Hz, H-21), 1.67 (3H, bd, *J*=1.4 Hz, H-30), 2.04 (s, 3H, **CH_3**CO), 2.07 (2H, bs, H-16), 2.89 (1H, bt, *J*=12.5 Hz, H-13), 3.00 (1H, bt, *J*=11.5 Hz, H-19), 3.17 (1H, dd, J_1=4.8 Hz, J_2=11.5 Hz, H-3), 3.35-4.10 (2H, m, H-1'), 3.35-4.10 (2H, m, H-2'), 3.35-4.10 (2H, m, H-3'), 3.35-4.10 (2H, m, H-4'), 3.35-4.10 (2H, m, H-5'), 3.82 (3H, s, H-11'), 4.57 (1H, bs, H-29), 4.72 (1H, bs, H-29), 6.86-6.92 (2H, m, H-9'), 7.35-7.41 (2H, m, H-8'). ^{13}C NMR (150.90 MHz, $CDCl_3$): δ [ppm] 14.70 (q, C27), 15.30 (q, C24), 16.10 (q, C25), 16.20 (q, C26), 18.30 (t, C6), 19.60 (q, C30), 21.10 (t, C11), 21.29 (**CH_3**CO), 25.60 (t, C12), 27.20 (d, C13), 27.40 (t, C2),

28.00 (q, C23), 28.30 (t, C4'), 30.20 (t, C21), 31.40 (t, C15), 32.20 (t, C16), 34.40 (t, C22), 36.10 (t, C7), 37.00 (s, C10), 38.70 (s, C4), 38.80 (t, C1), 40.70 (s, C8), 42.00 (s, C14), 45.60 (t, C3'), 45.80 (d, C19), 47.70 (t, C5'), 48.10 (t, C1'), 50.80 (d, C9), 53.00 (d, C18), 55.10 (s, C17), 55.30 (q, C11'), 55.40 (d, C5), 79.00 (d, C3), 109.20 (t, C29), 113.70 (d, C9'), 128.00 (s, C7'), 128.40 (d, C8'), 151.40 (s, C20), 160.70 (s, C10'), 171.03 (CH_3**CO**), 171.40 (s, C6'), 174.90 (s, C28).

(3β)-3-Hydroxy-28-[*N*-(4-methoxybenzoyl)homopiperazin-1-yl]lup-20(29)-en-28-one (**14**)
^{1}H NMR (600.13 MHz, $CDCl_3$): δ [ppm] 0.68 (1H, bdd, J_1=1.8 Hz, J_2=11.2 Hz, H-5), 0.75 (3H, s, H-25), 0.82 (3H, s, H-26), 0.93 (3H, s, H-24), 0.96 (3H, s, H-23), 0.96 (3H, s, H-27), 1.16 (2H, bt, *J*=13.3 Hz, H-21), 1.67 (3H, bd, *J*=1.4 Hz, H-30), 2.07 (2H, bs, H-16), 2.89 (1H, bt, *J*=12.5 Hz, H-13), 3.00 (1H, bt, *J*=11.5 Hz, H-19), 3.17 (1H, dd, J_1=4.8 Hz, J_2=11.5 Hz, H-3), 3.35-4.10 (2H, m, H-1'), 3.35-4.10 (2H, m, H-2'), 3.35-4.10 (2H, m, H-3'), 3.35-4.10 (2H, m, H-4'), 3.35-4.10 (2H, m, H-5'), 3.82 (3H, s, H-11'), 4.57 (1H, bs, H-29), 4.72 (1H, bs, H-29), 6.86-6.92 (2H, m, H-9'), 7.35-7.41 (2H, m, H-8'). ^{13}C NMR (150.90 MHz, $CDCl_3$): δ [ppm] 14.70 (q, C27), 15.30 (q, C24), 16.10 (q, C25), 16.20 (q, C26), 18.30 (t, C6), 19.60 (q, C30), 21.10 (t, C11), 25.60 (t, C12), 27.20 (d, C13), 27.40 (t, C2), 28.00 (q, C23), 28.30 (t, C4'), 30.20 (t, C21), 31.40 (t, C15), 32.20 (t, C16), 34.40 (t, C22), 36.10 (t, C7), 37.00 (s, C10), 38.70 (s, C4), 38.80 (t, C1), 40.70 (s, C8), 42.00 (s, C14), 45.60 (t, C3'), 45.80 (d, C19), 47.70 (t, C5'), 48.10 (t, C1'), 50.80 (d, C9), 53.00 (d, C18), 55.10 (s, C17), 55.30 (q, C11'), 55.40 (d, C5), 79.00 (d, C3), 109.20 (t, C29), 113.70 (d, C9'), 128.00 (s, C7'), 128.40 (d, C8'), 151.40 (s, C20), 160.70 (s, C10'), 171.4 (s, C6'), 174.90 (s, C28).

Part I. NMR spectra (Fig. S1– S26) and 3D structures of the selected compounds (Fig. S27–S29)

Figure S1. (3β)-3-(acetyloxy)lup-20(29)-en-28-oic acid (**2**): ^{1}H NMR spectrum measured (top) and calculated by ChemBioDraw Ultra, v. 12.0 (bottom)

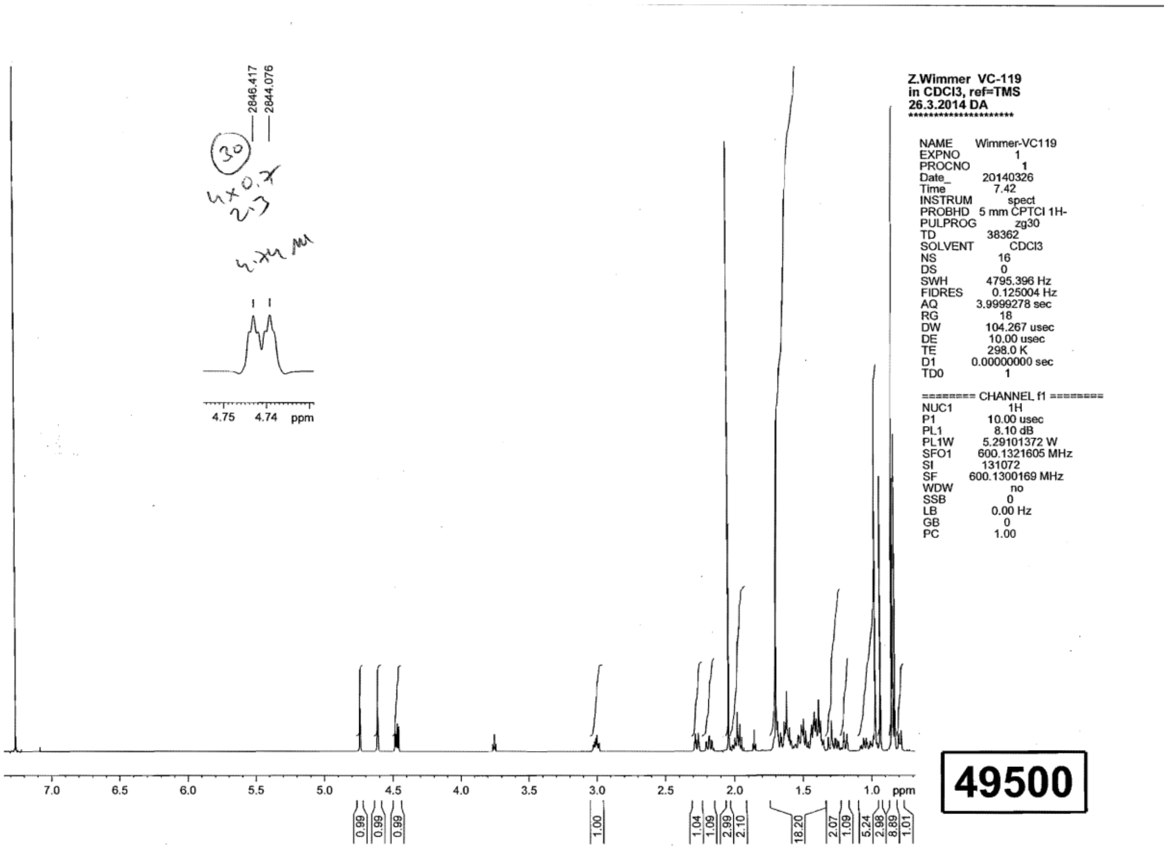

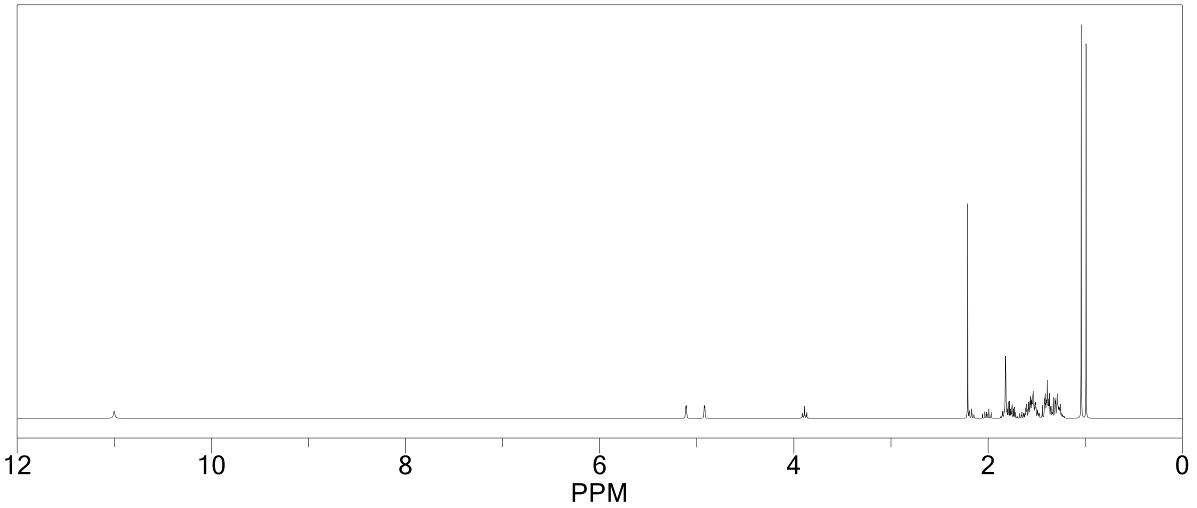

Figure S2. (3β)-3-(acetyloxy)lup-20(29)-en-28-oic acid (**2**): ^{13}C NMR spectrum measured (top) and calculated by ChemBioDraw Ultra, v. 12.0 (bottom)

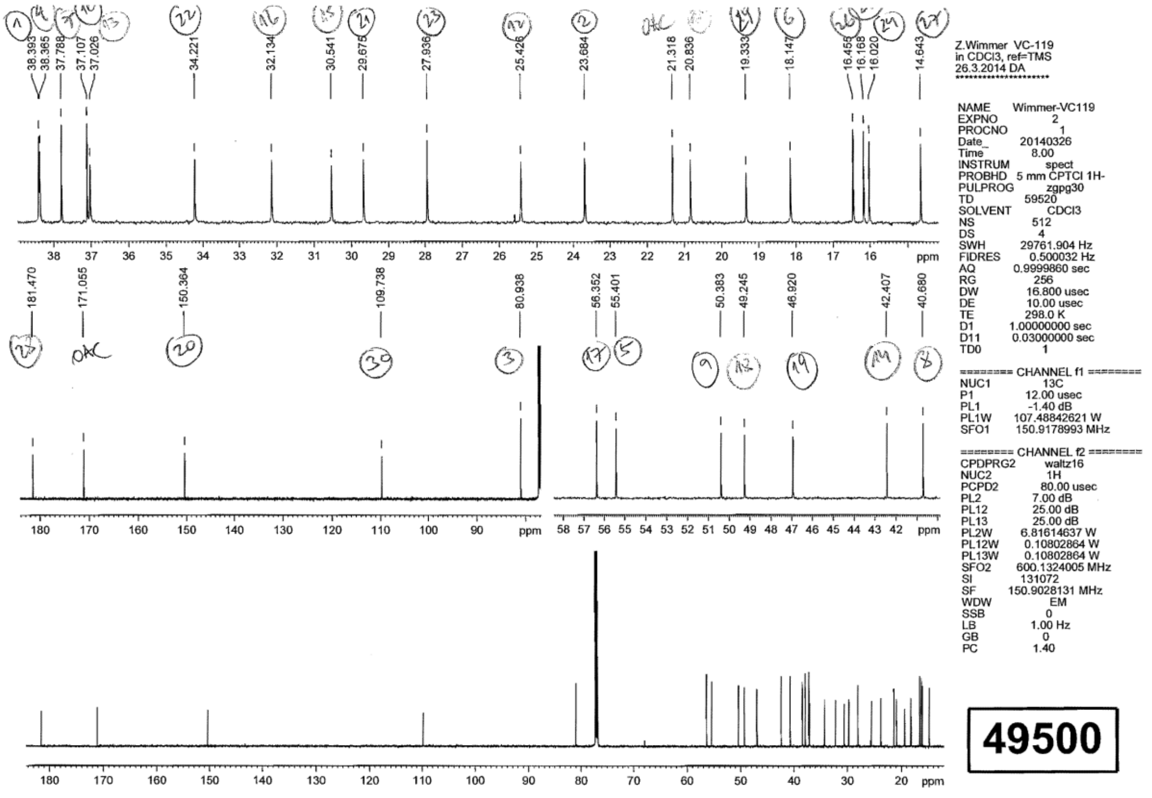

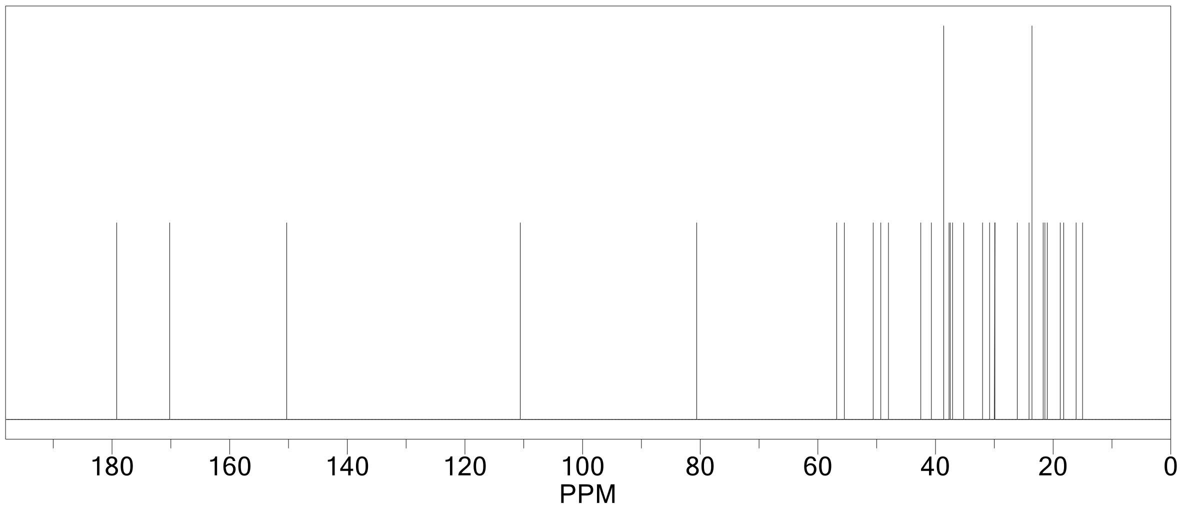

Figure S3. *tert*-Butyl-4-[(3β)-3-(acetyloxy)-28-oxolup-20(29)-en-28-yl]ethylenediamine-1-carboxylate (**3**): ^{1}H NMR spectrum measured (top) and calculated by ChemBioDraw Ultra, v. 12.0 (bottom)

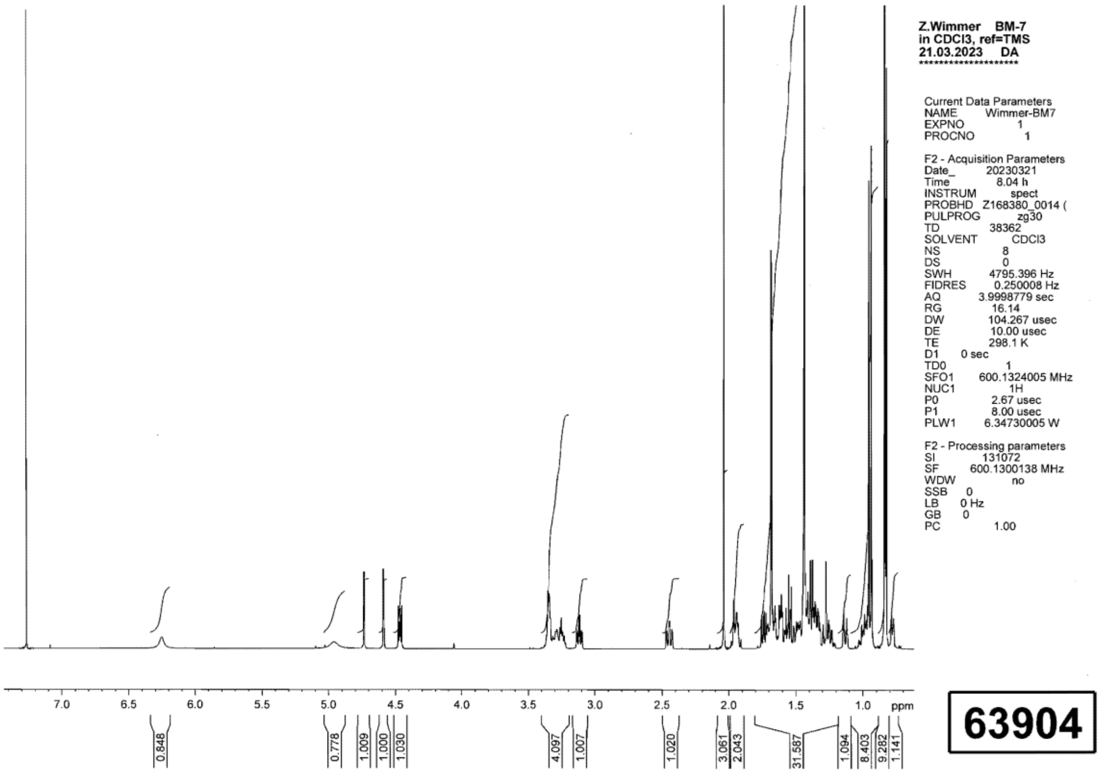

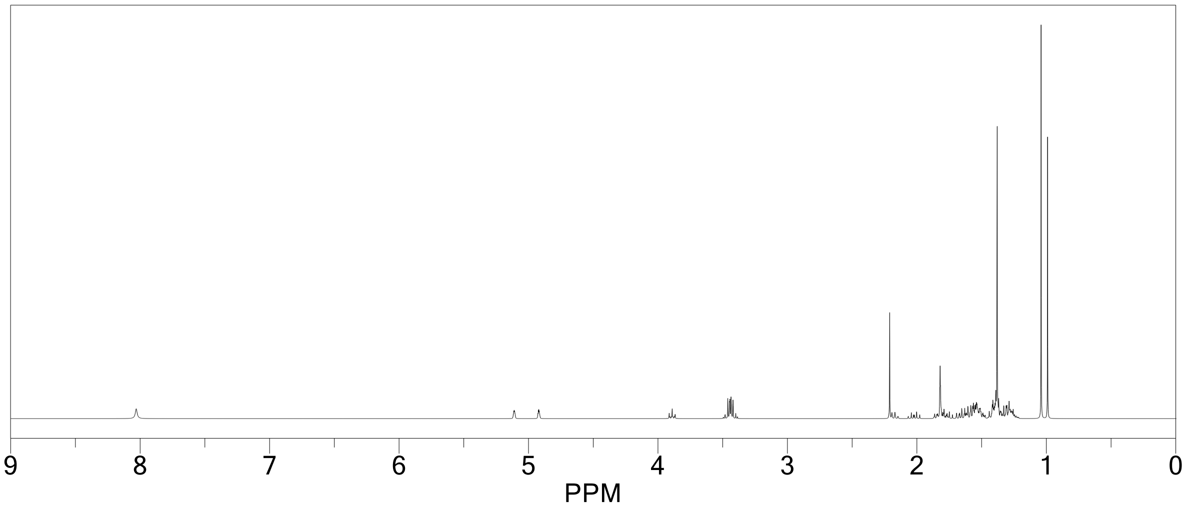

Figure S4. *tert*-Butyl-4-[(3β)-3-(acetyloxy)-28-oxolup-20(29)-en-28-yl]ethylenediamine-1-carboxylate (**3**): ^{13}C NMR spectrum measured (top) and calculated by ChemBioDraw Ultra, v. 12.0 (bottom)

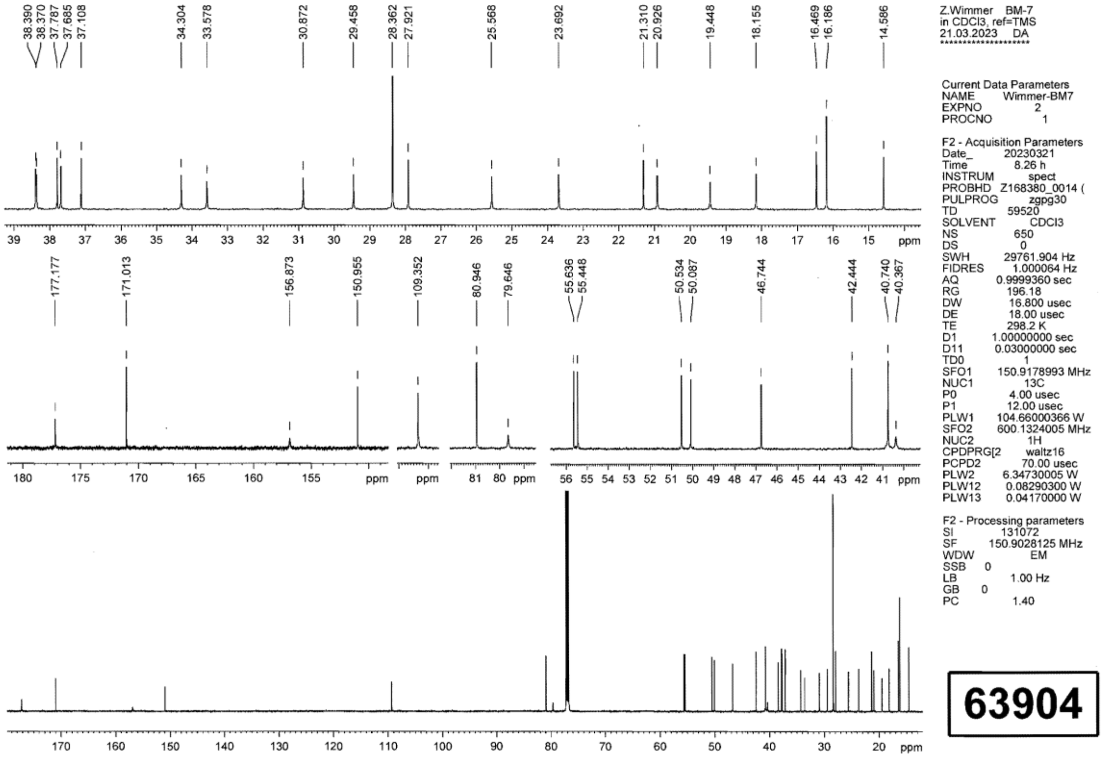

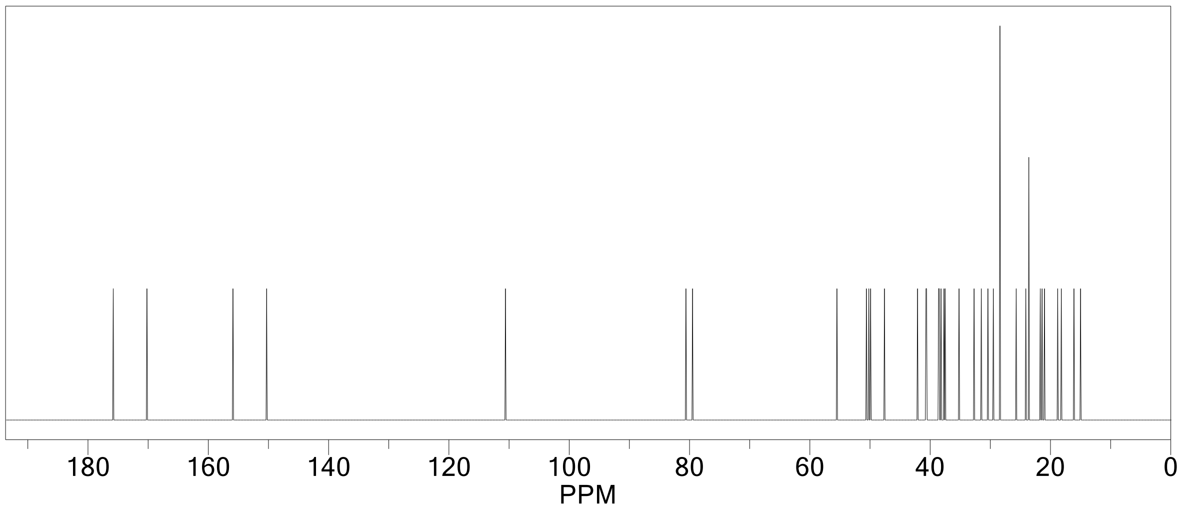

Figure S5. (3β)-28-Oxo-28-(ethylenediamin-1-yl)lup-20(29)-en-3-yl acetate (**4**): ^{1}H NMR spectrum measured (top) and calculated by ChemBioDraw Ultra, v. 12.0 (bottom)

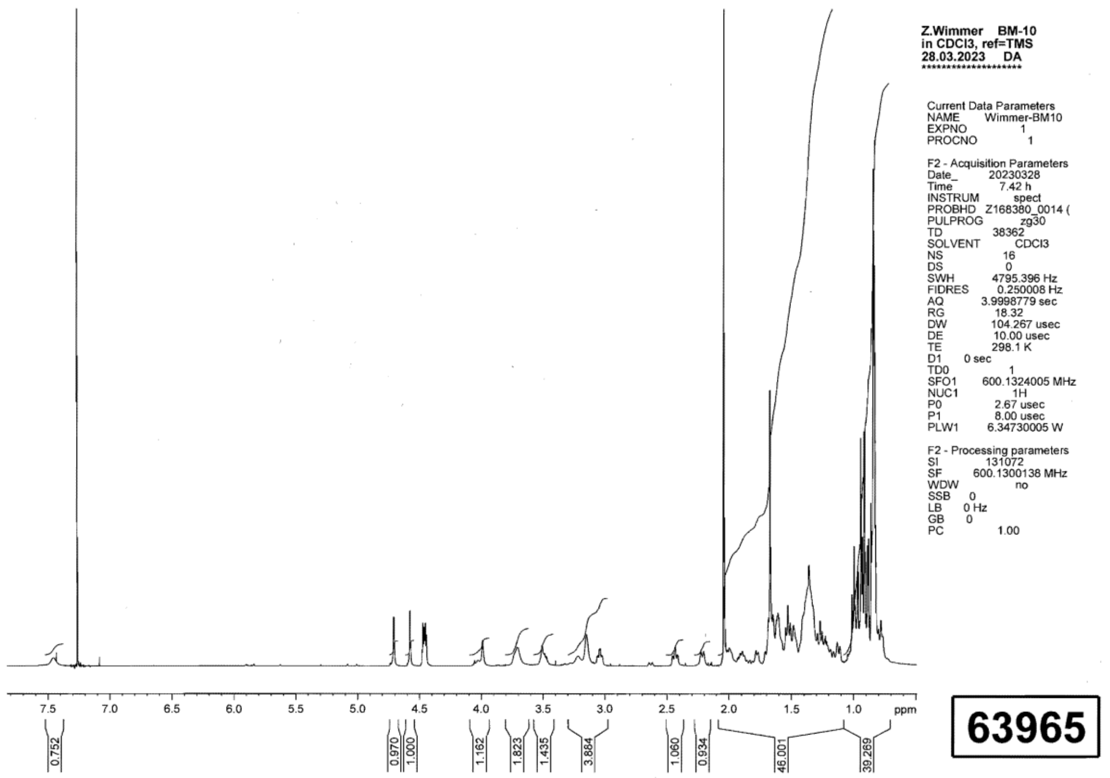

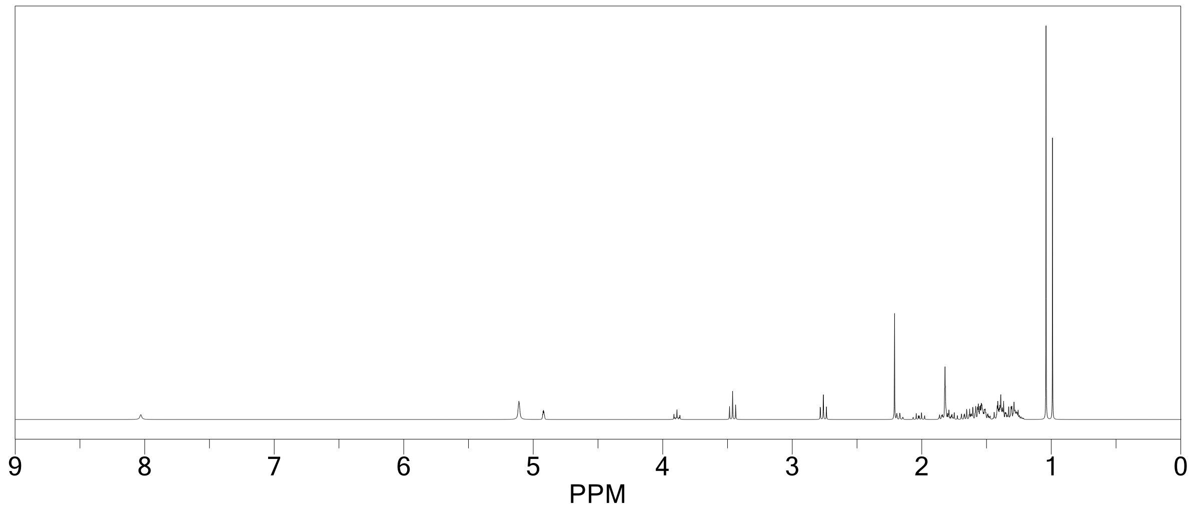

Figure S6. (3β)-28-Oxo-28-(ethylenediamin-1-yl)lup-20(29)-en-3-yl acetate (**4**): ^{13}C NMR spectrum measured (top) and calculated by ChemBioDraw Ultra, v. 12.0 (bottom)

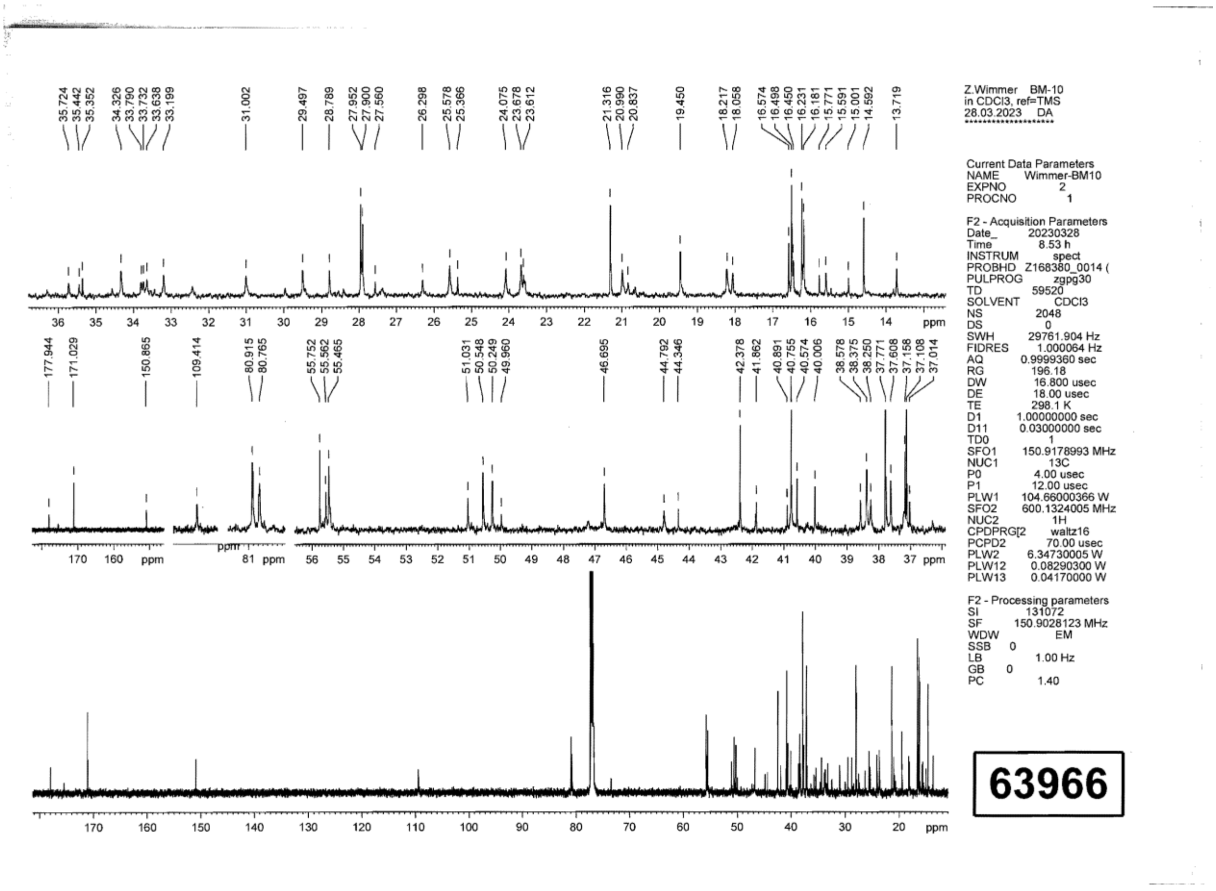

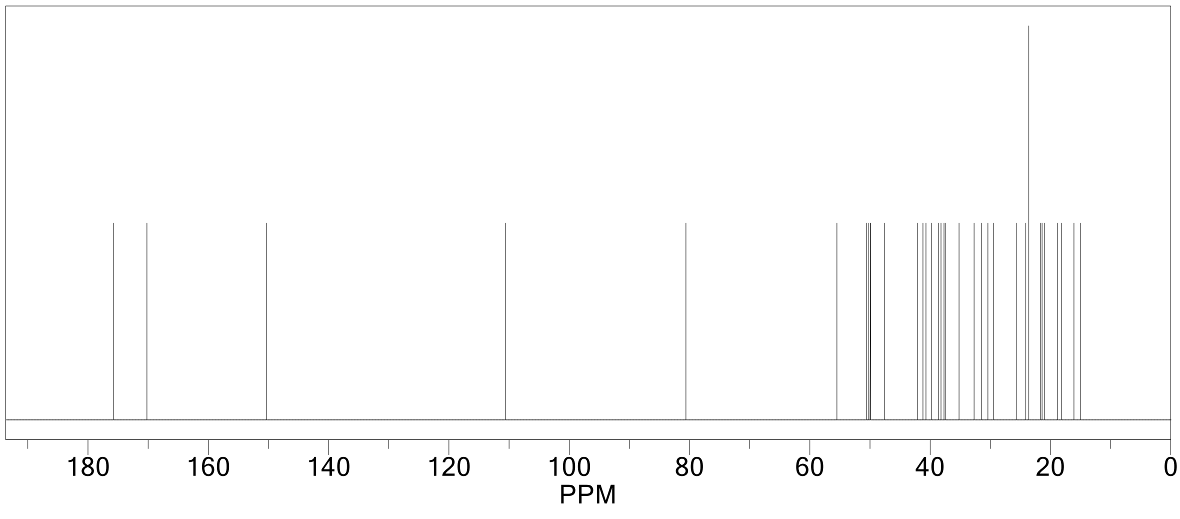

Figure S7. (3β)-28-Oxo-28-[*N*-(4-methoxybenzoyl)ethylenediamin-1-yl]lup-20(29)-en-3-yl acetate (**5**): ^{1}H NMR spectrum measured (top) and calculated by ChemBioDraw Ultra, v. 12.0 (bottom)

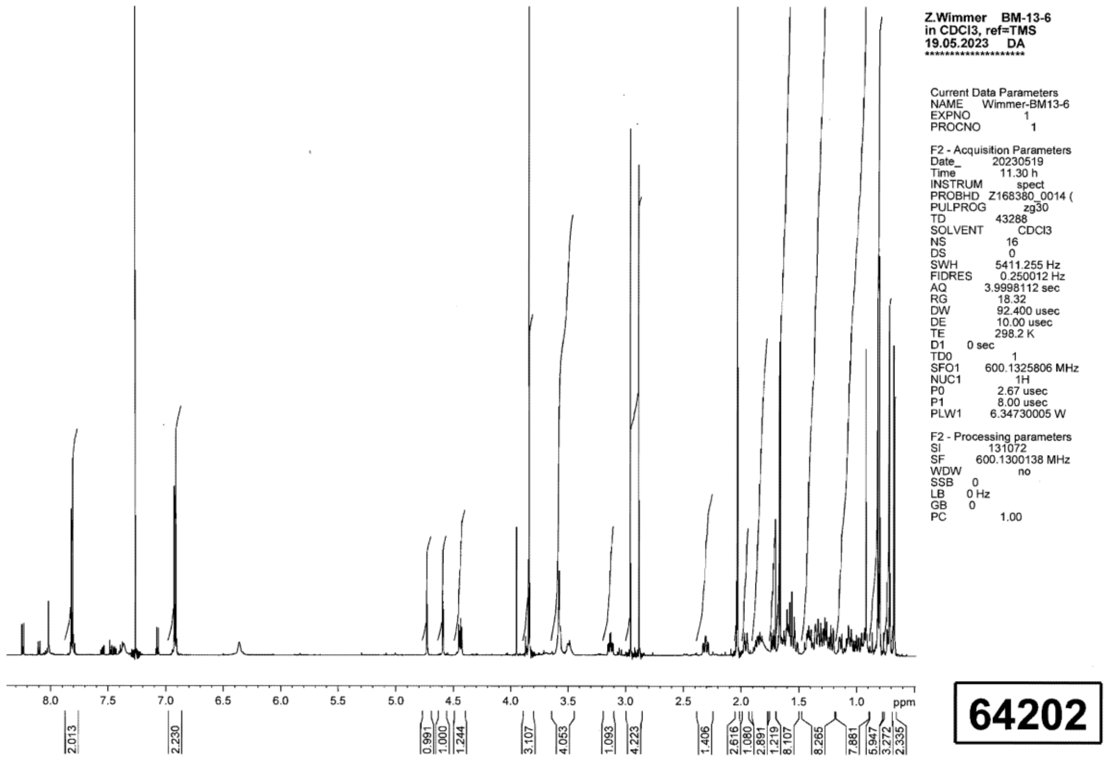

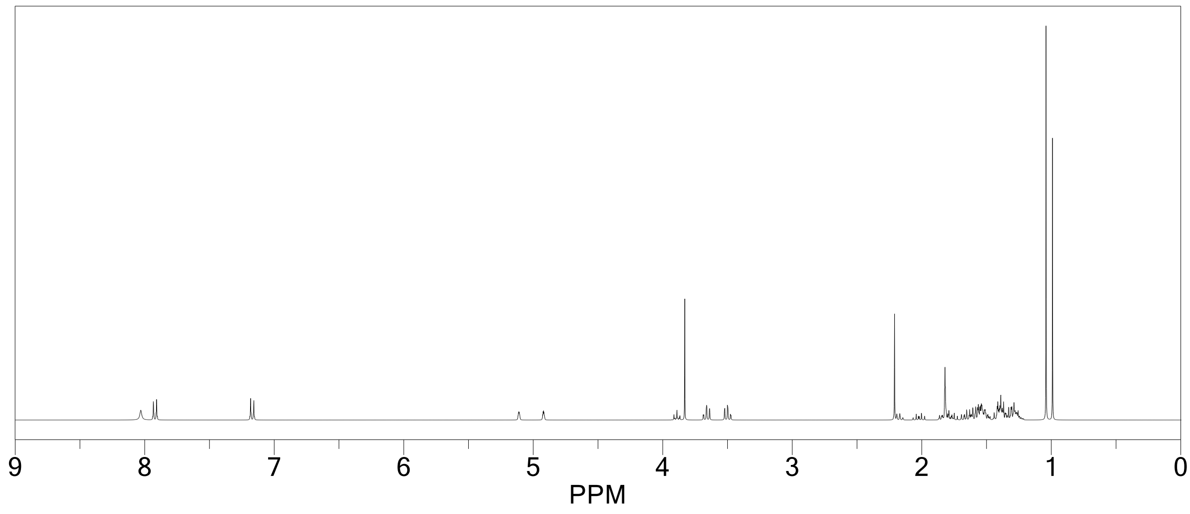

Figure S8. (3β)-28-Oxo-28-[*N*-(4-methoxybenzoyl)ethylenediamin-1-yl]lup-20(29)-en-3-yl acetate (**5**): ^{1}H NMR spectrum measured (top) and calculated by ChemBioDraw Ultra, v. 12.0 (bottom)

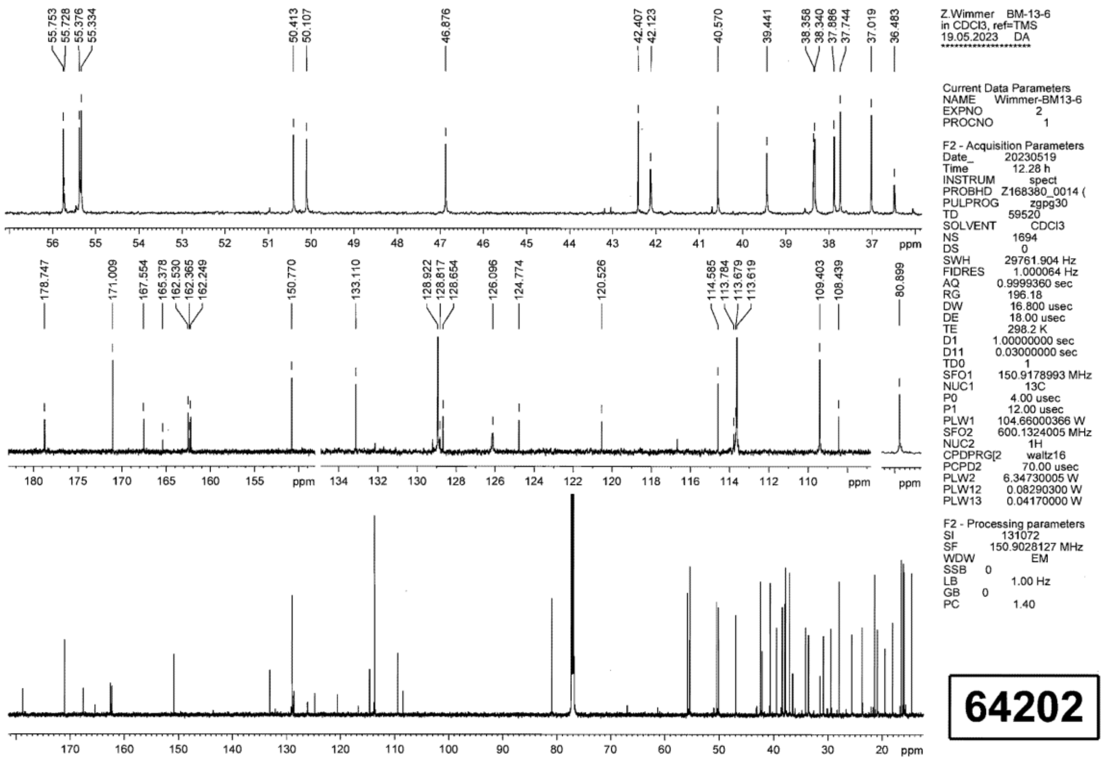

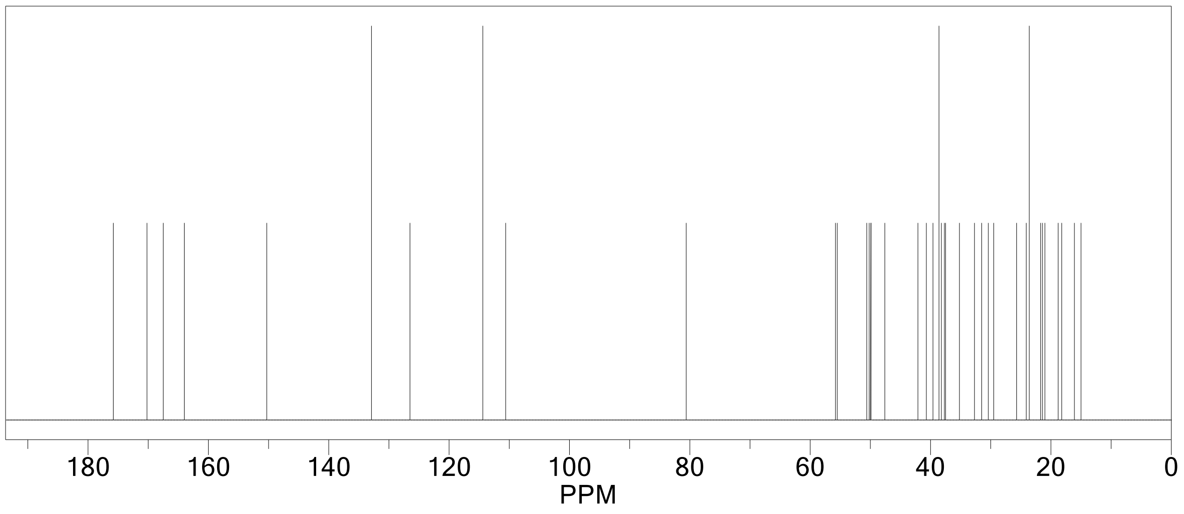

Figure S9. (3β)-3-Hydroxy-28-[*N*-(4-methoxybenzoyl)ethylenediamin-1-yl]lup-20(29)-en-28-one (**6**): ^{1}H NMR spectrum measured (top) and calculated by ChemBioDraw Ultra, v. 12.0 (bottom)

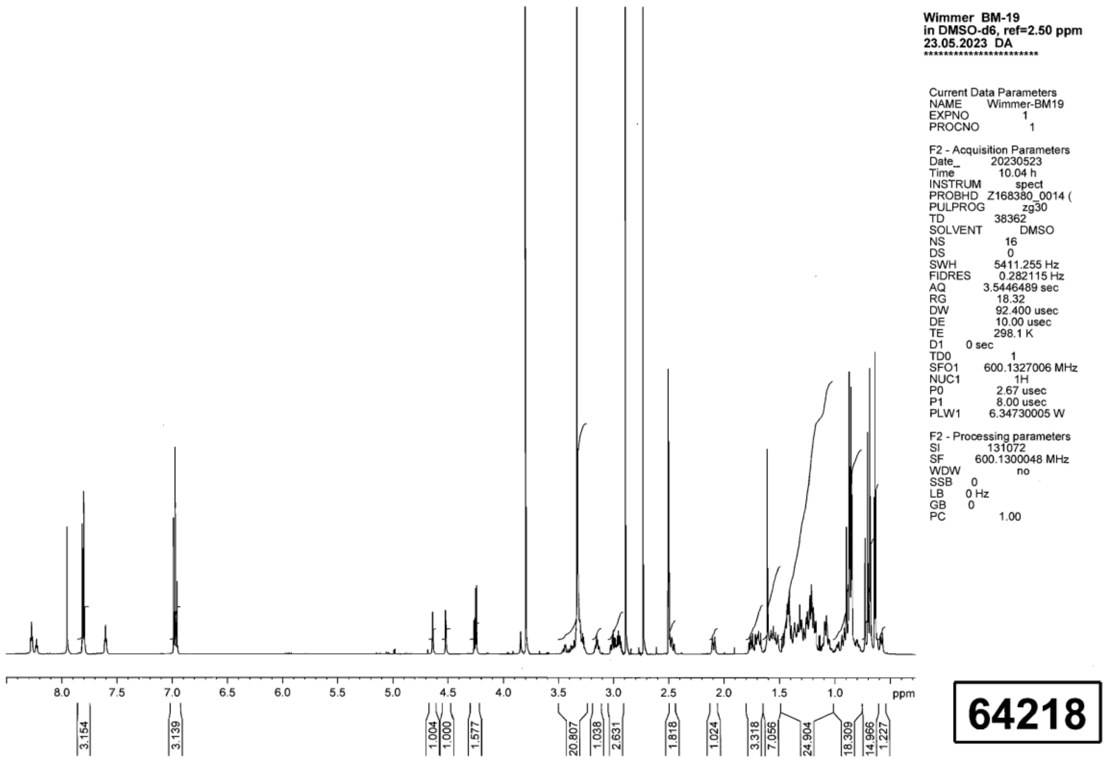

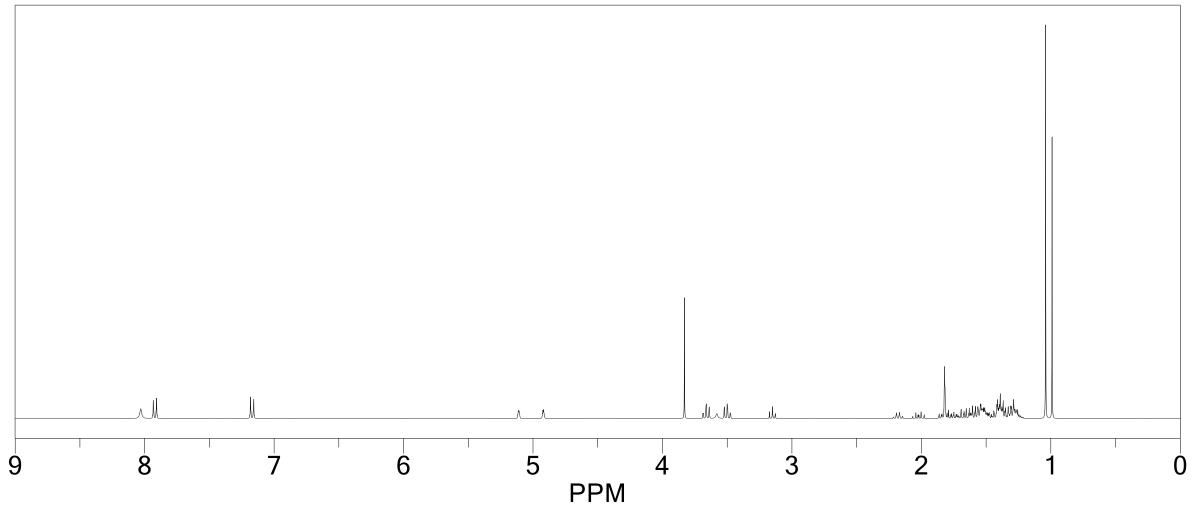

Figure S10. (3β)-3-Hydroxy-28-[*N*-(4-methoxybenzoyl)ethylenediamin-1-yl]lup-20(29)-en-28-one (**6**): ^{13}C NMR spectrum measured (top and center), and calculated by ChemBioDraw Ultra, v. 12.0 (bottom)

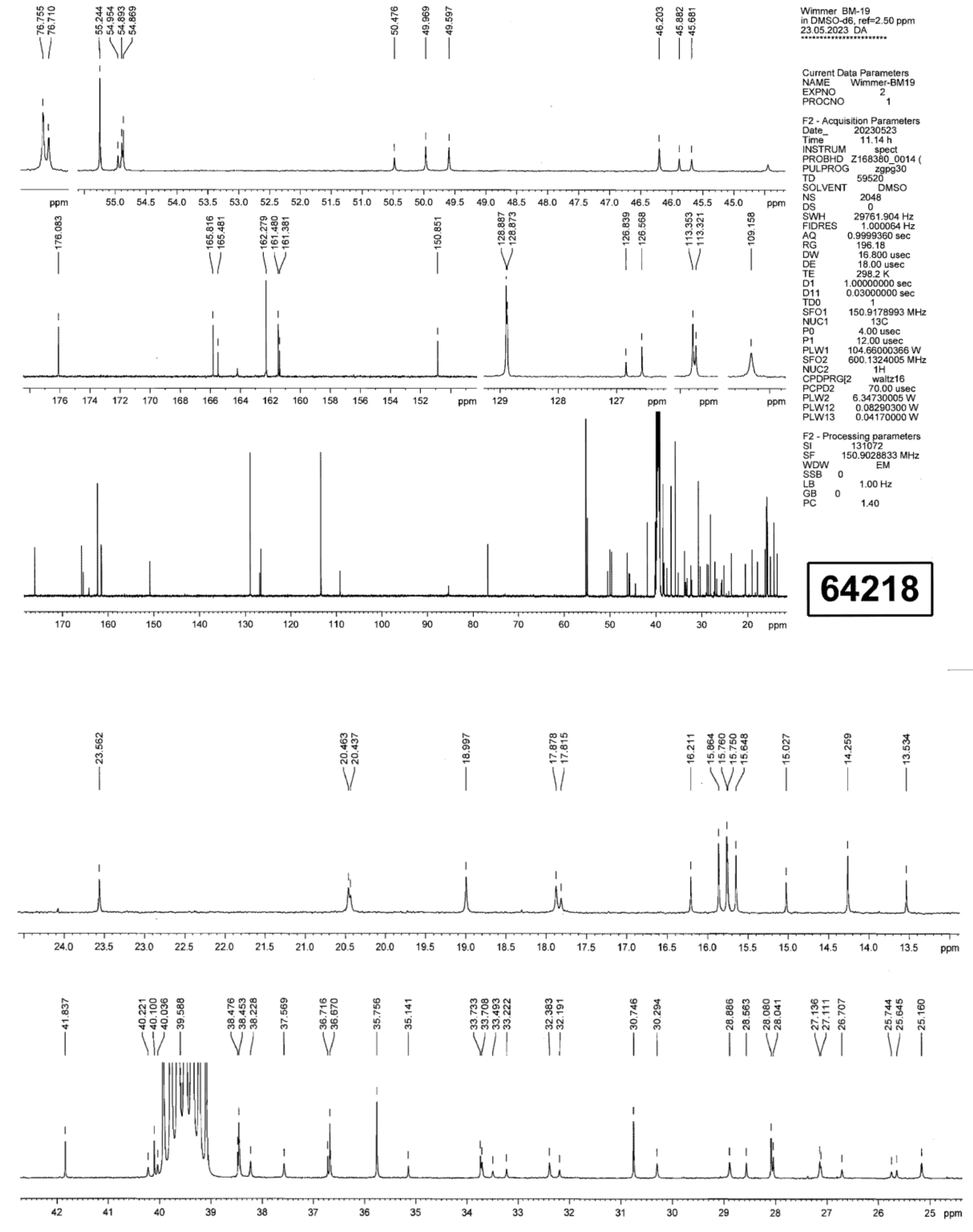

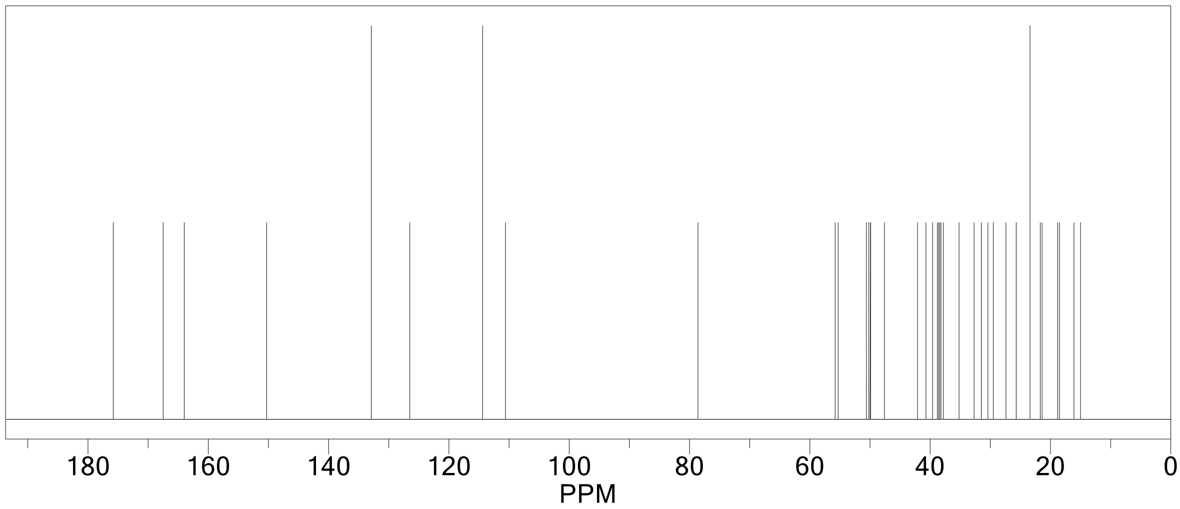

180
160
140
120
100
80
60
40
20
0
PPM

Figure S11. *tert*-Butyl 4-[(3β)-3-(acetyloxy)-28-oxolup-20(29)-en-28-yl]piperazin-1-carboxylate (**7**): ^{1}H NMR spectrum measured (top) and calculated by ChemBioDraw Ultra, v. 12.0 (bottom)

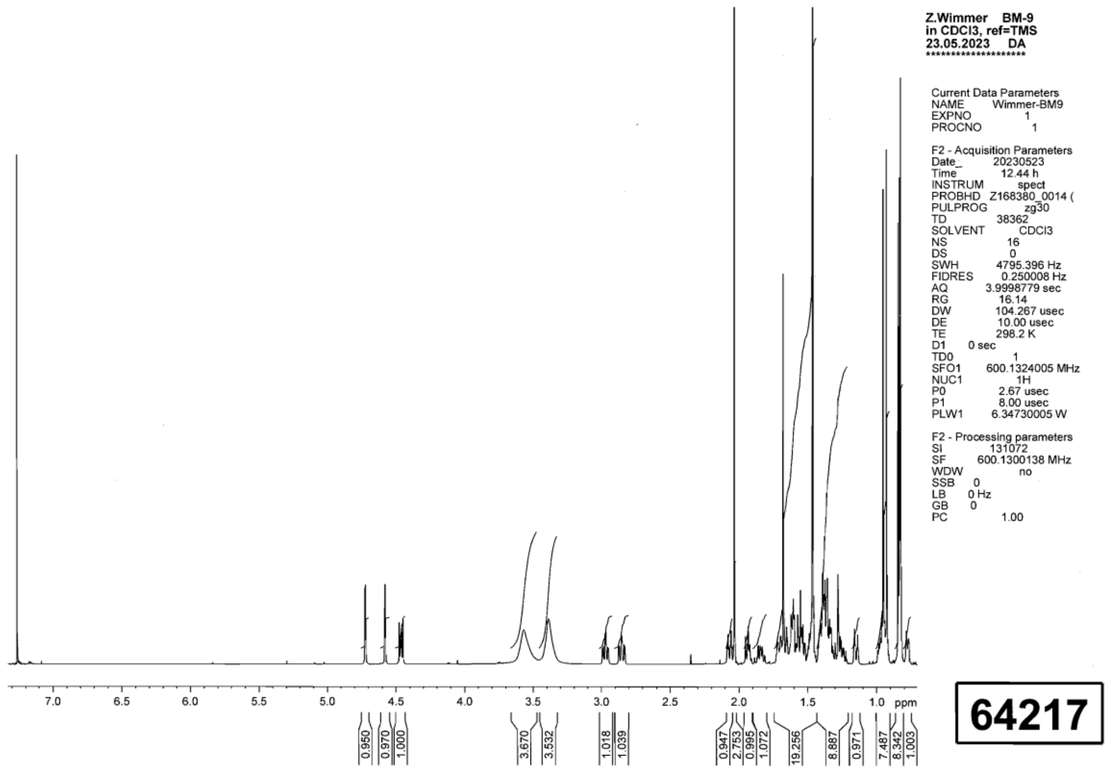

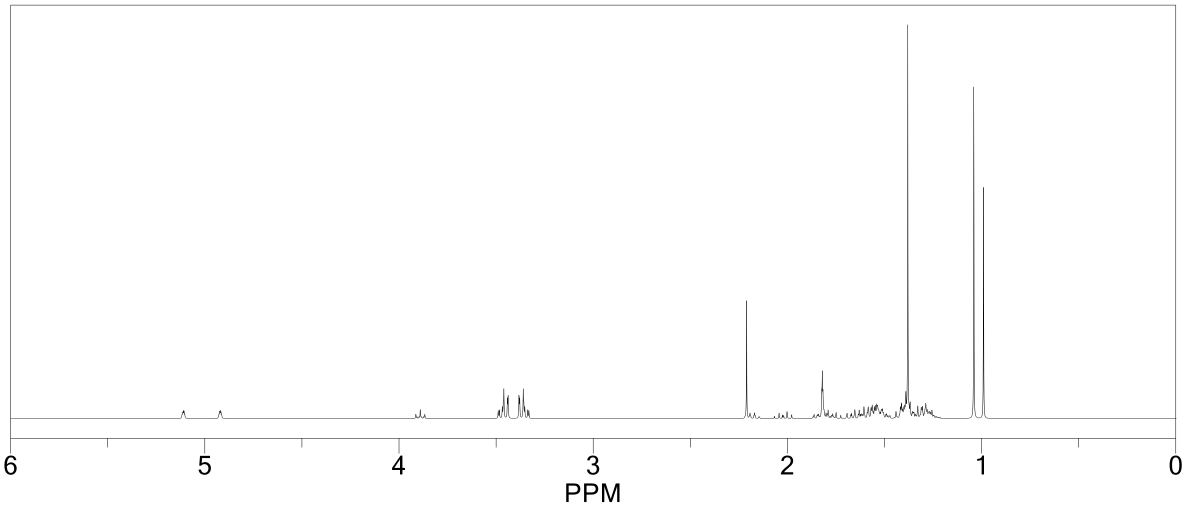

Figure S12. *tert*-Butyl 4-[(3β)-3-(acetyloxy)-28-oxolup-20(29)-en-28-yl]piperazin-1-carboxylate (**7**): ^{13}C NMR spectrum measured (top) and calculated by ChemBioDraw Ultra, v. 12.0 (bottom)

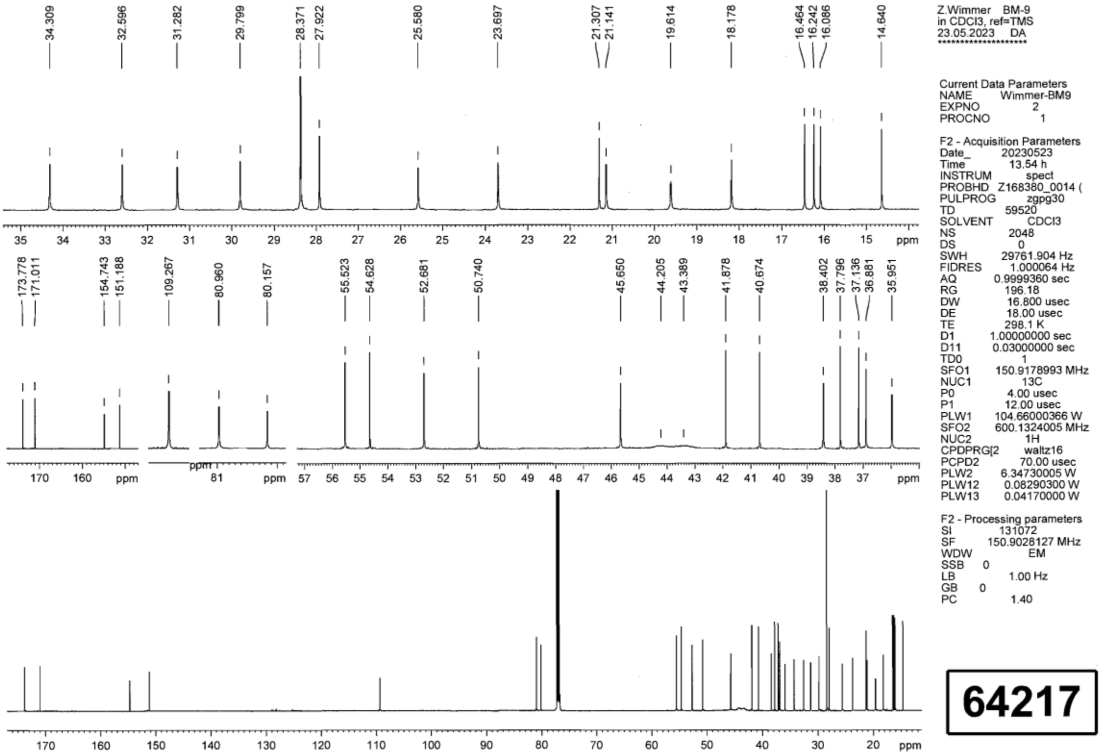

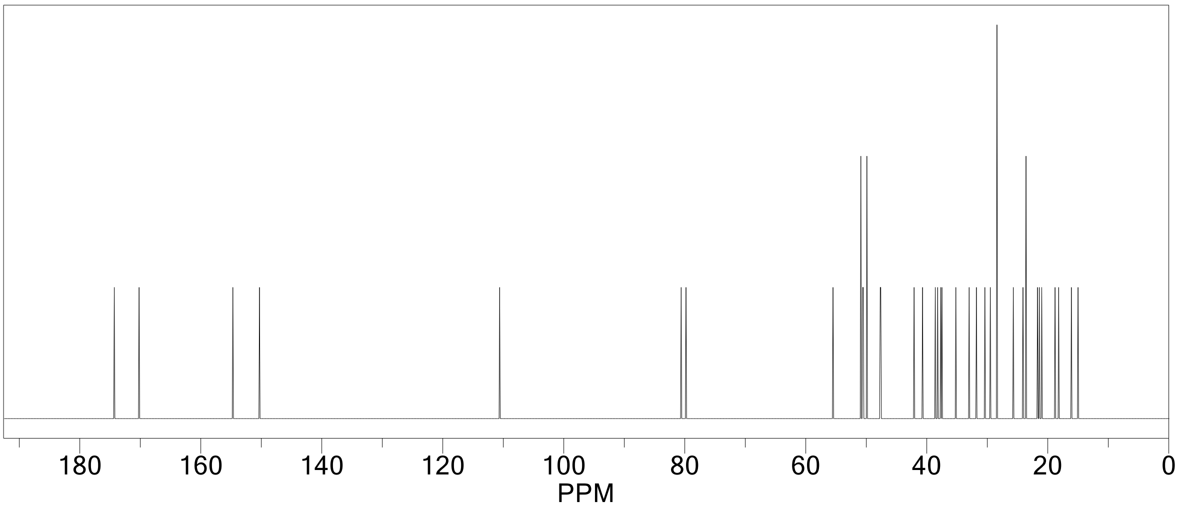

Figure S13. (3β)-28-Oxo-28-(piperazin-1-yl)lup-20(29)-en-3-yl acetate (**8**): ^{1}H NMR spectrum measured (top) and calculated by ChemBioDraw Ultra, v. 12.0 (bottom)

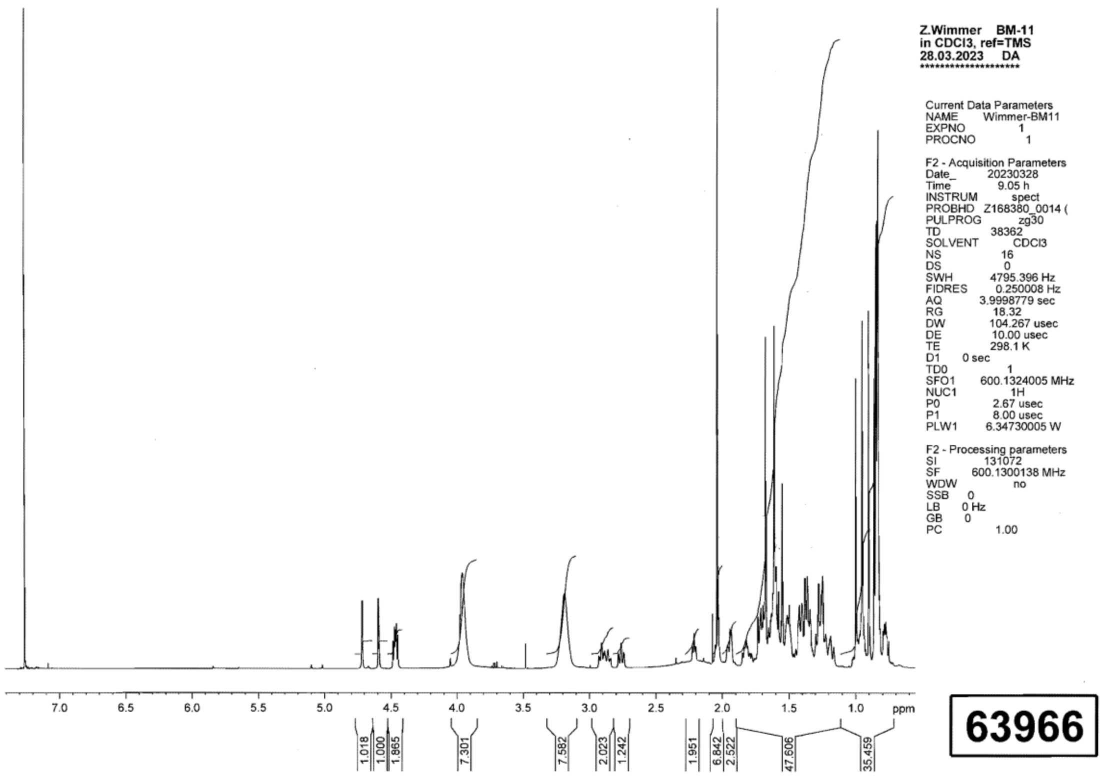

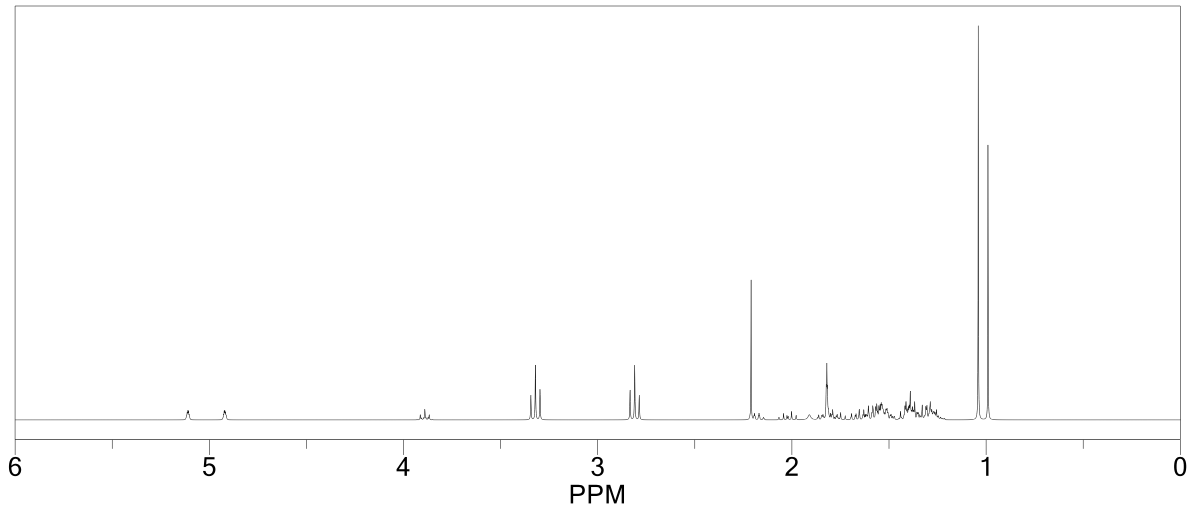

Figure S14. (3β)-28-Oxo-28-(piperazin-1-yl)lup-20(29)-en-3-yl acetate (**8**): ^{13}C NMR spectrum measured (top) and calculated by ChemBioDraw Ultra, v. 12.0 (bottom)

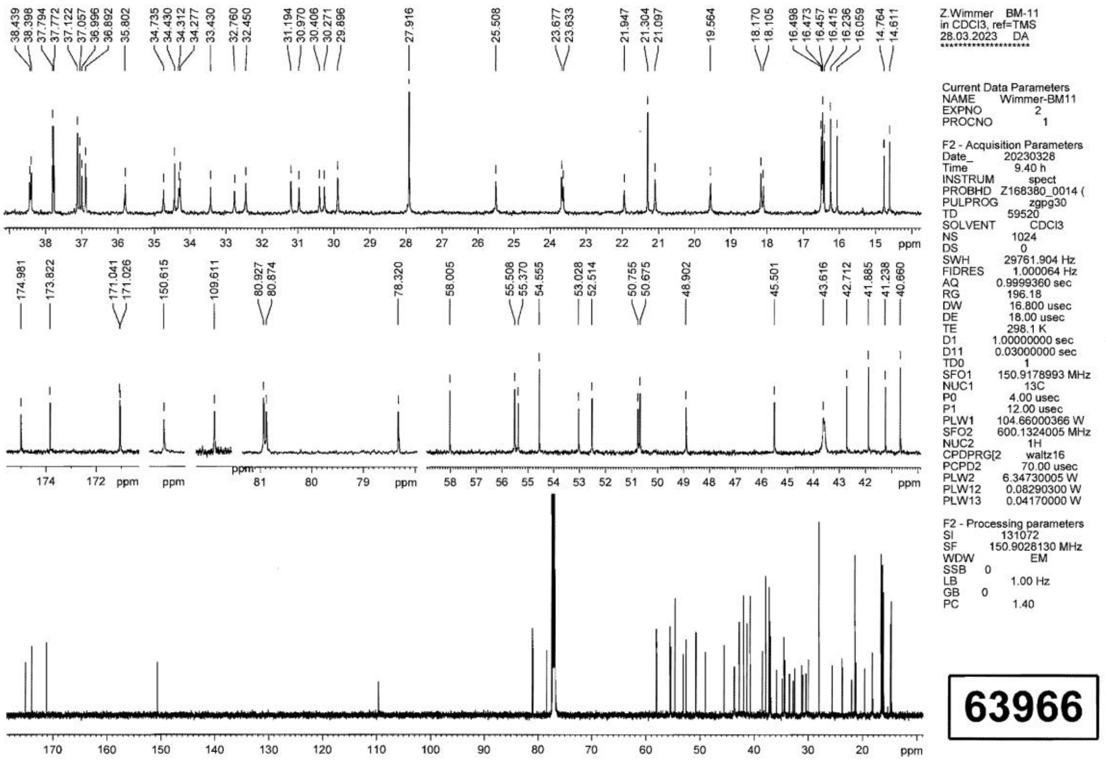

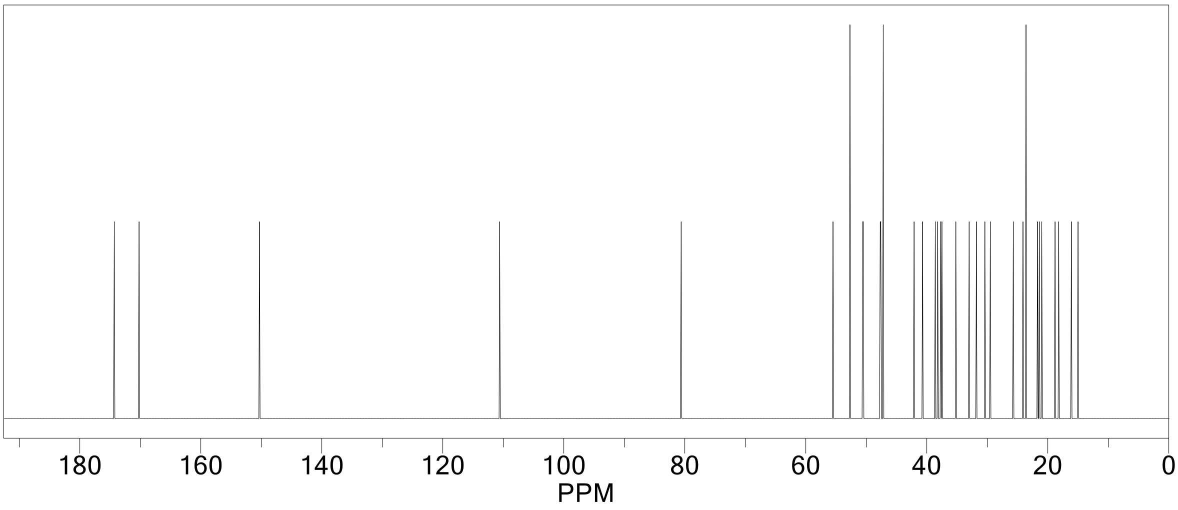

Figure S15. (3β)-28-Oxo-28-[*N*-(4-methoxybenzoyl)piperazin-1-yl]lup-20(29)-en-3-yl acetate (**9**): ^{1}H NMR spectrum measured (top) and calculated by ChemBioDraw Ultra, v. 12.0 (bottom)

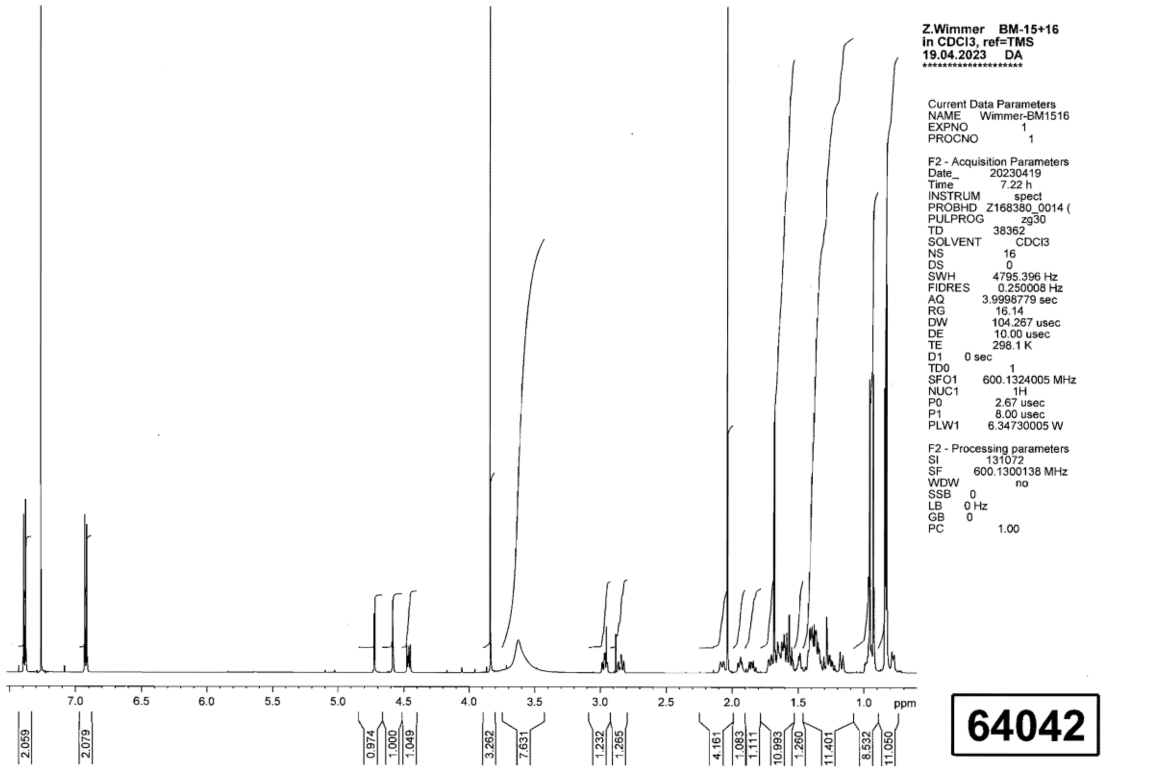

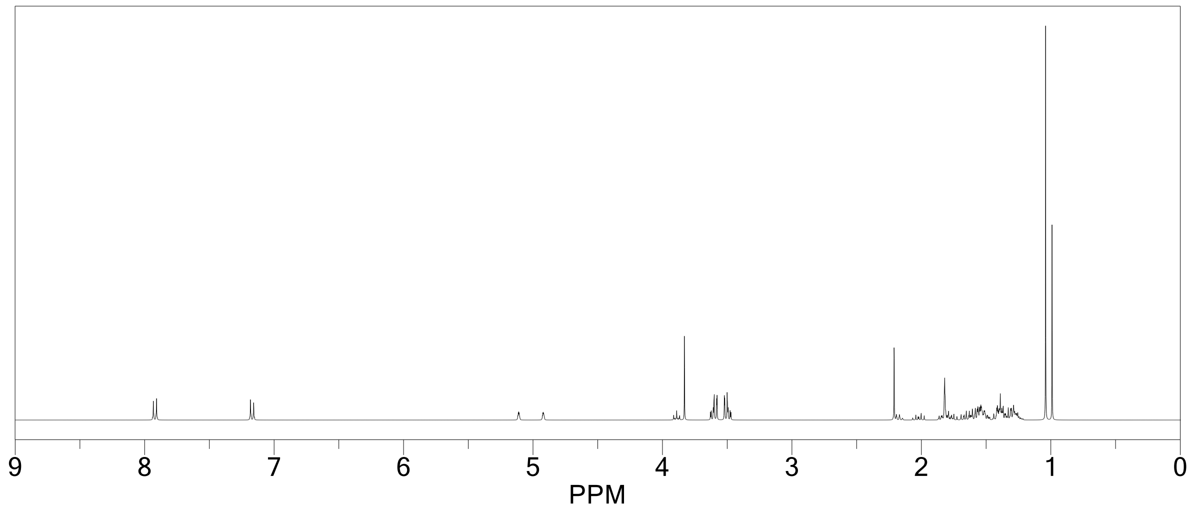

Figure S16. (3β)-28-Oxo-28-[*N*-(4-methoxybenzoyl)piperazin-1-yl]lup-20(29)-en-3-yl acetate (**9**): ^{13}C NMR spectrum measured (top) and calculated by ChemBioDraw Ultra, v. 12.0 (bottom)

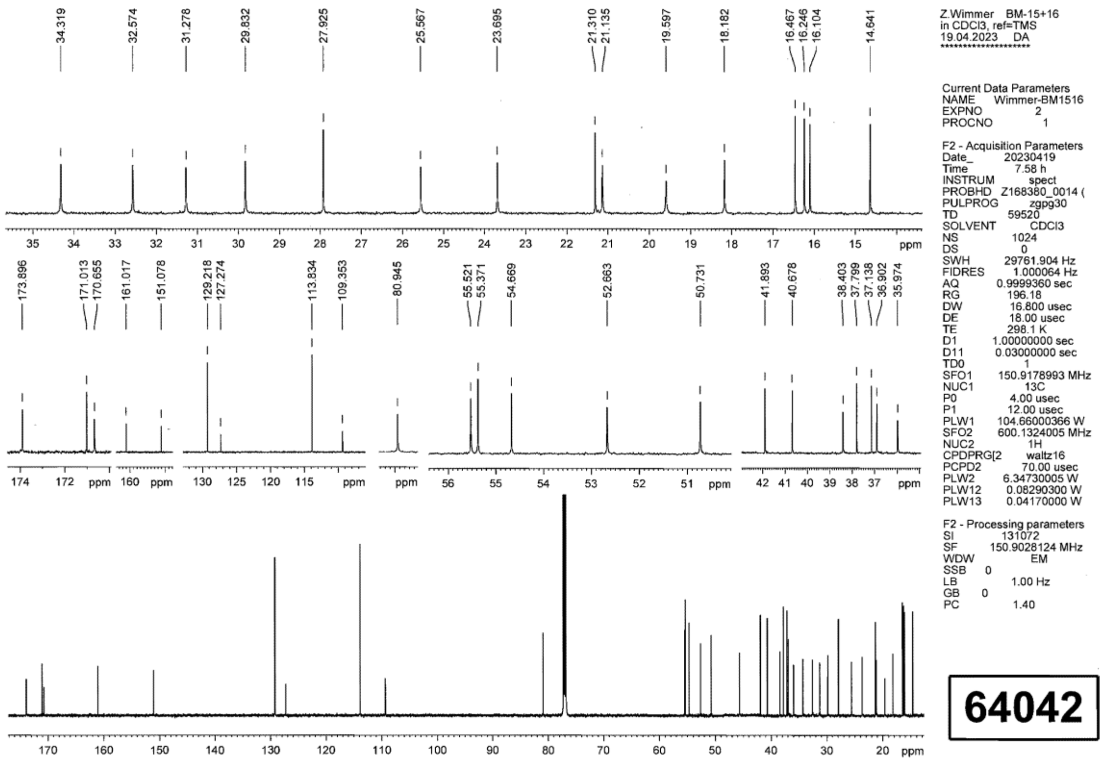

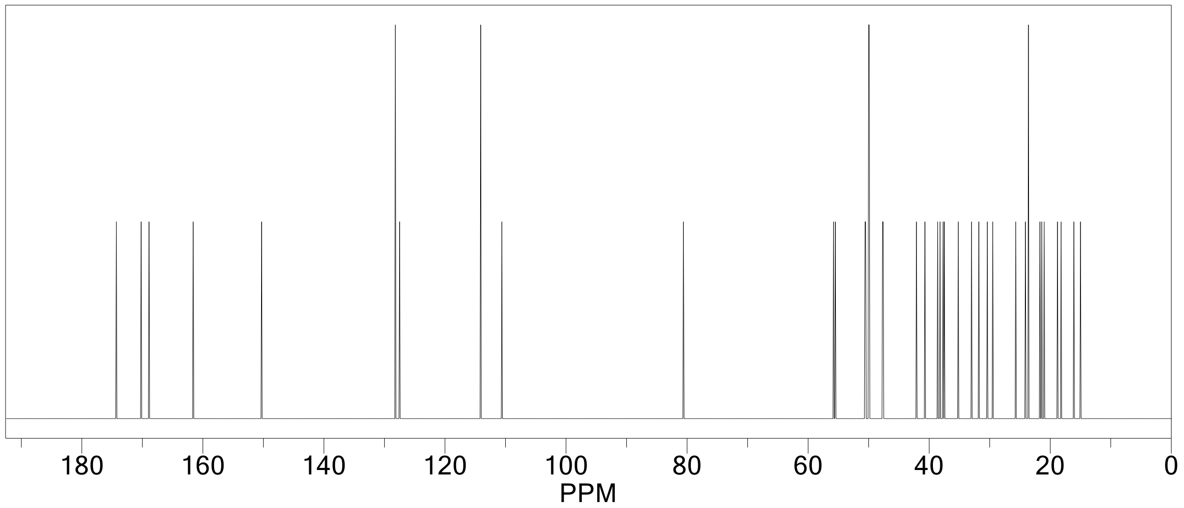

Figure S17. (3β)-3-Hydroxy-28-[*N*-(4-methoxybenzoyl)piperazin-1-yl]lup-20(29)-en-28-one (**10**): ^{1}H NMR spectrum measured (top) and calculated by ChemBioDraw Ultra, v. 12.0 (bottom)

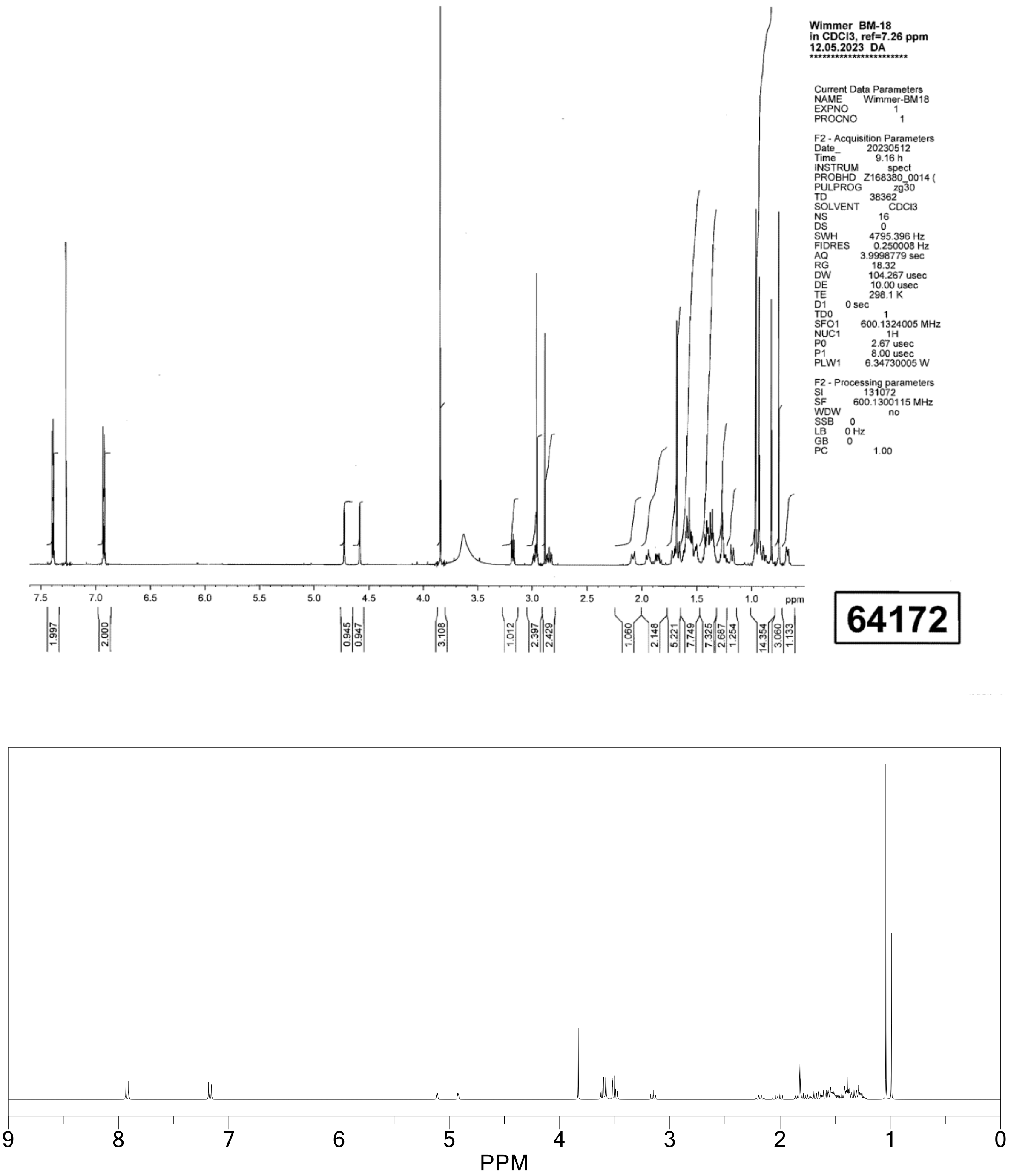

Figure S18. (3β)-3-Hydroxy-28-[*N*-(4-methoxybenzoyl)piperazin-1-yl]lup-20(29)-en-28-one (**10**): ^{1}H NMR spectrum measured (top) and calculated by ChemBioDraw Ultra, v. 12.0 (bottom)

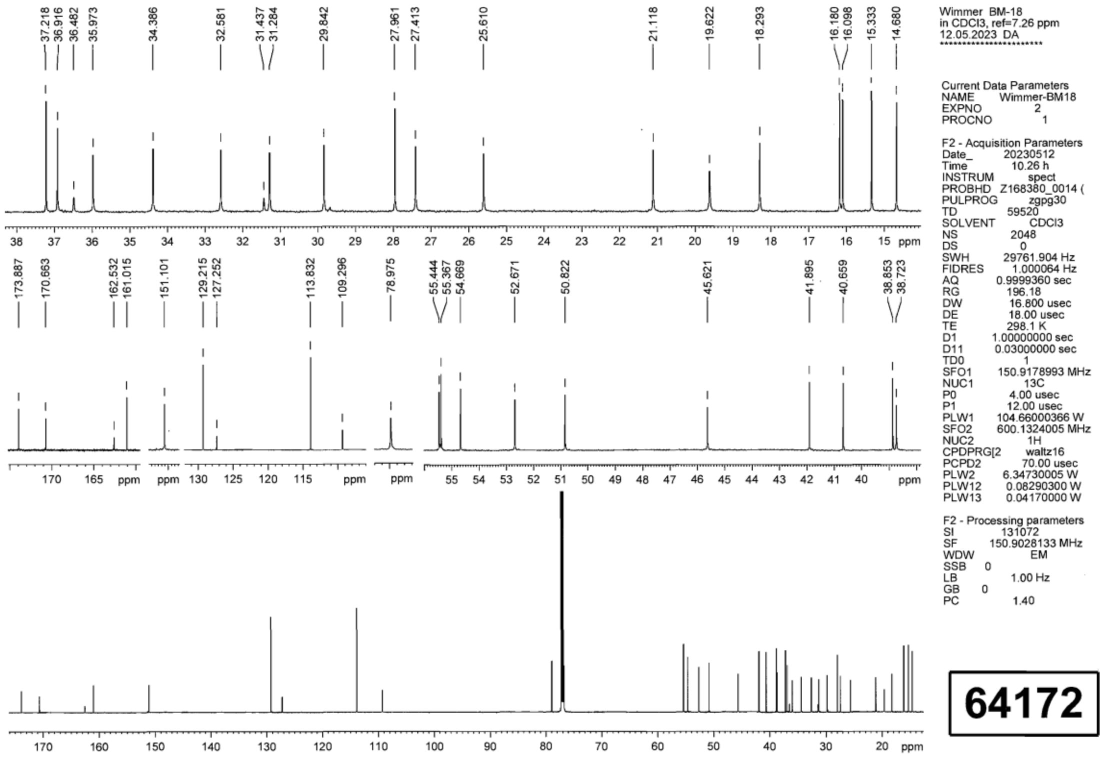

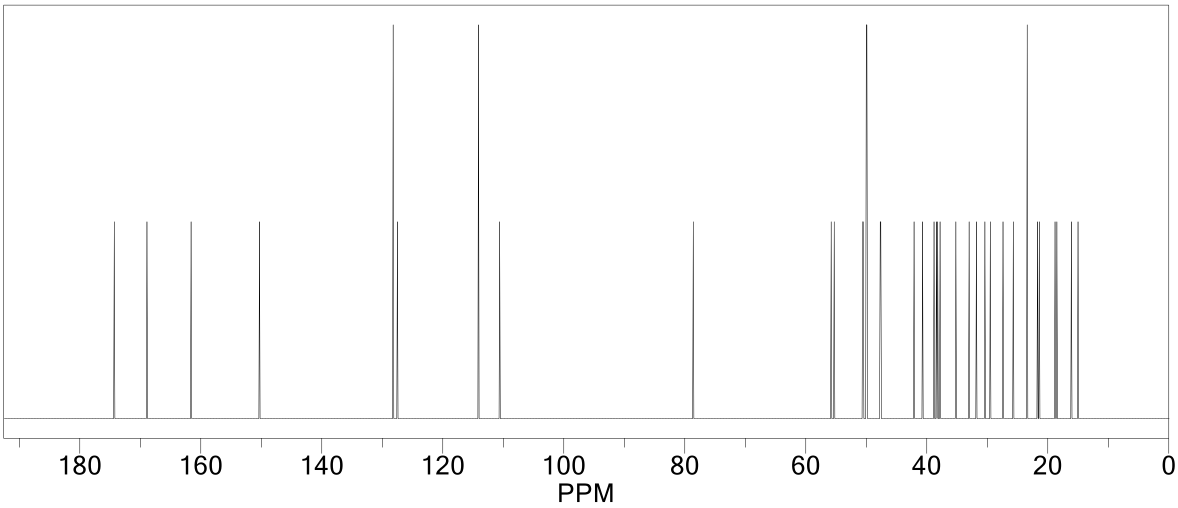

Figure S19. *tert*-Butyl 4-[(3β)-3-(acetyloxy)-28-oxolup-20(29)-en-28-yl]homopiperazin-1-carboxylate (**11**): ^{1}H NMR spectrum measured (top) and calculated by ChemBioDraw Ultra, v. 12.0 (bottom)

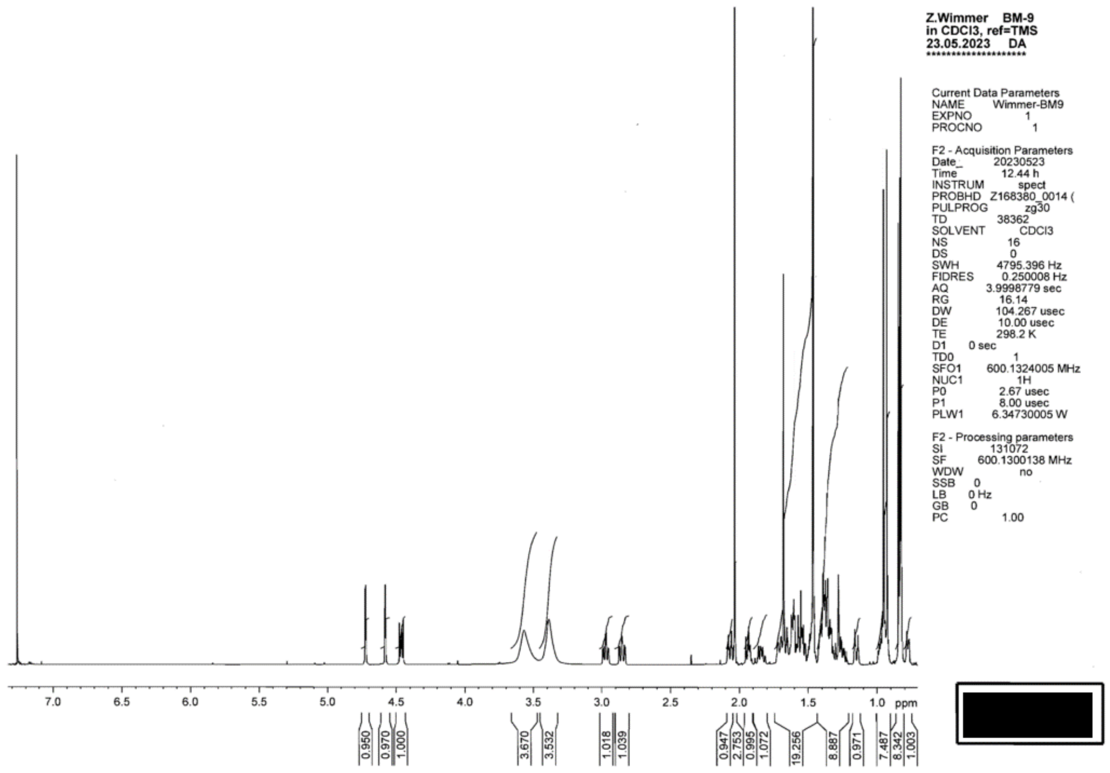

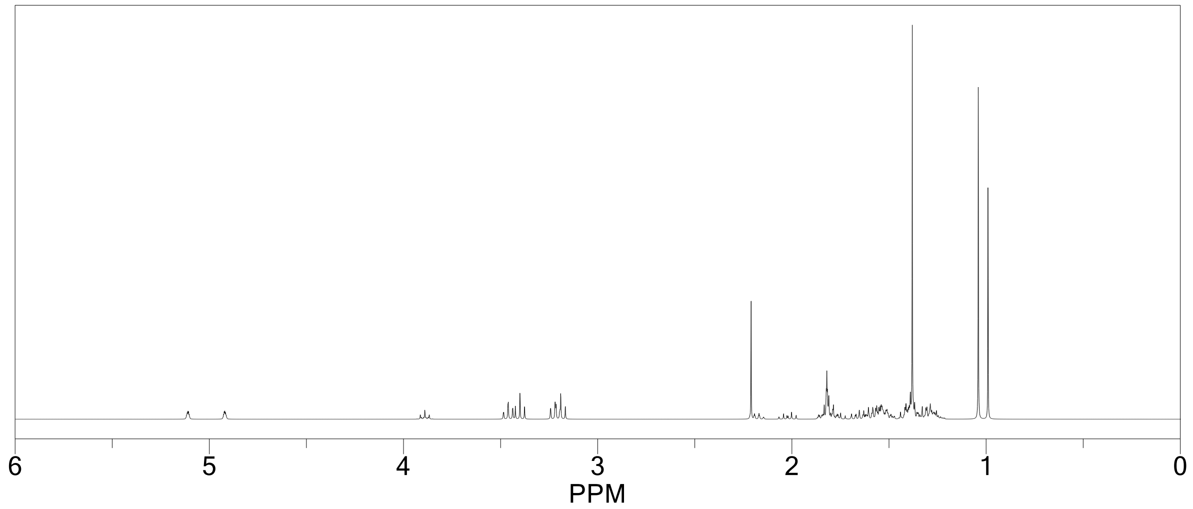

Figure S20. *tert*-Butyl 4-[(3β)-3-(acetyloxy)-28-oxolup-20(29)-en-28-yl]homopiperazin-1-carboxylate (**11**): ^{13}C NMR spectrum measured (top) and calculated by ChemBioDraw Ultra, v. 12.0 (bottom)

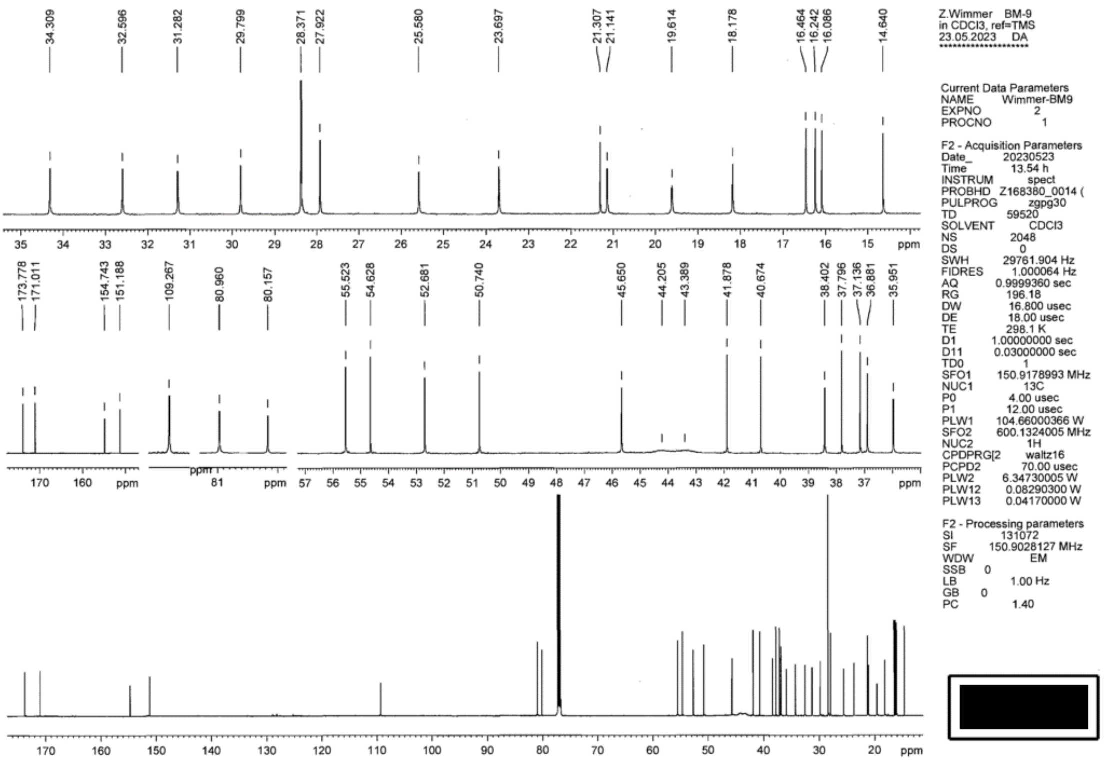

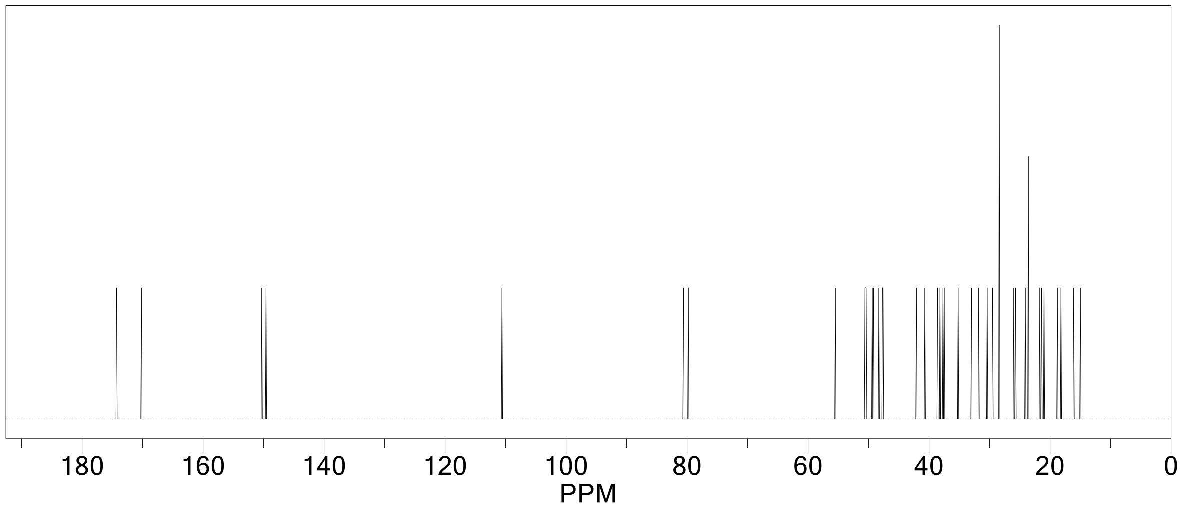

Figure S21. (3β)-28-Oxo-28-(homopiperazin-1-yl)lup-20(29)-en-3-yl acetate (**12**): ^{1}H NMR spectrum measured (top) and calculated by ChemBioDraw Ultra, v. 12.0 (bottom)

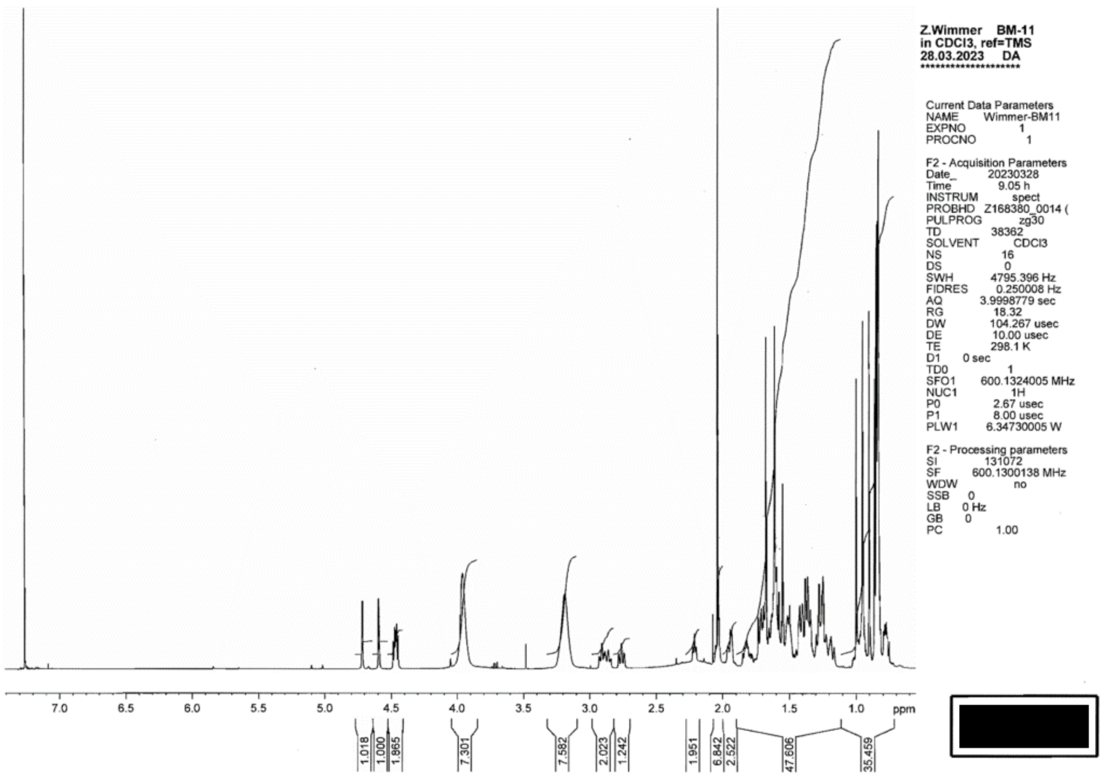

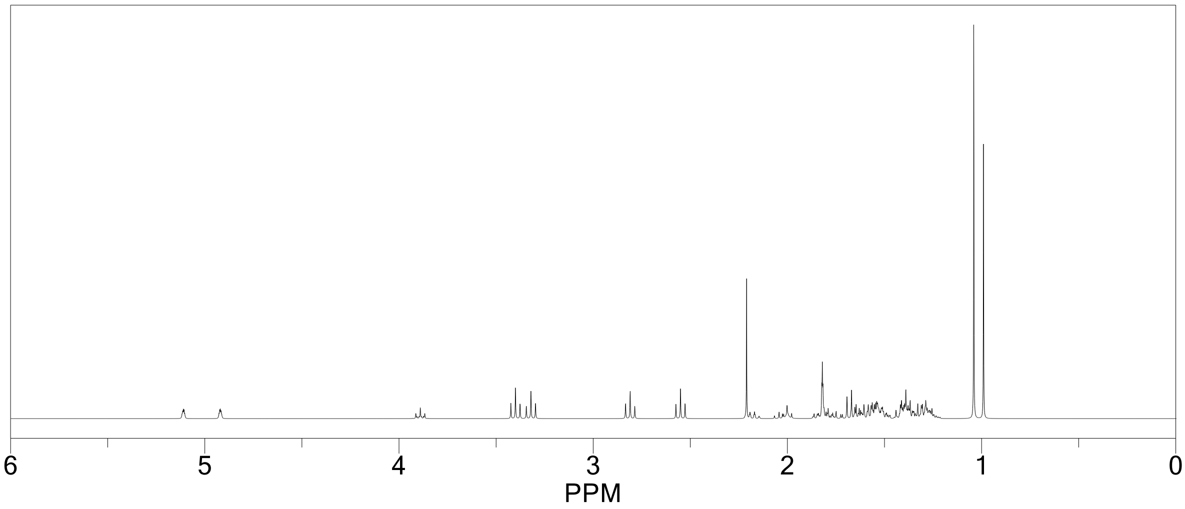

Figure S22. (3β)-28-Oxo-28-(homopiperazin-1-yl)lup-20(29)-en-3-yl acetate (**12**): ^{13}C NMR spectrum measured (top) and calculated by ChemBioDraw Ultra, v. 12.0 (bottom)

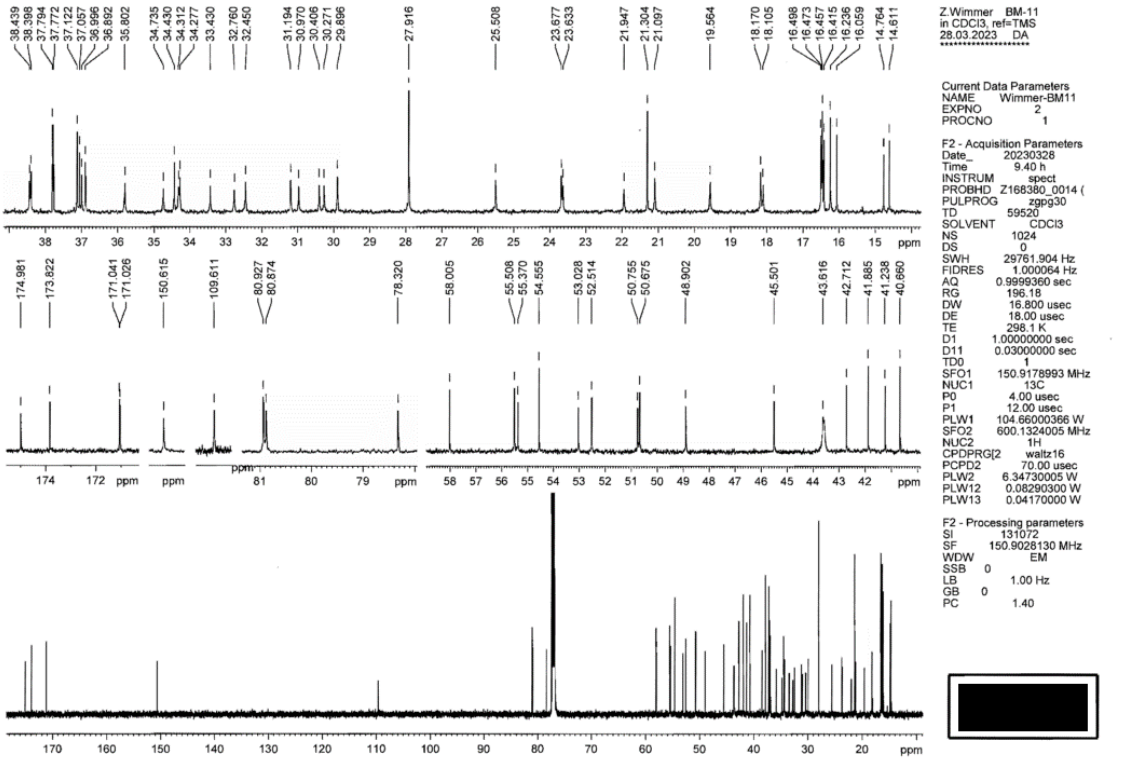

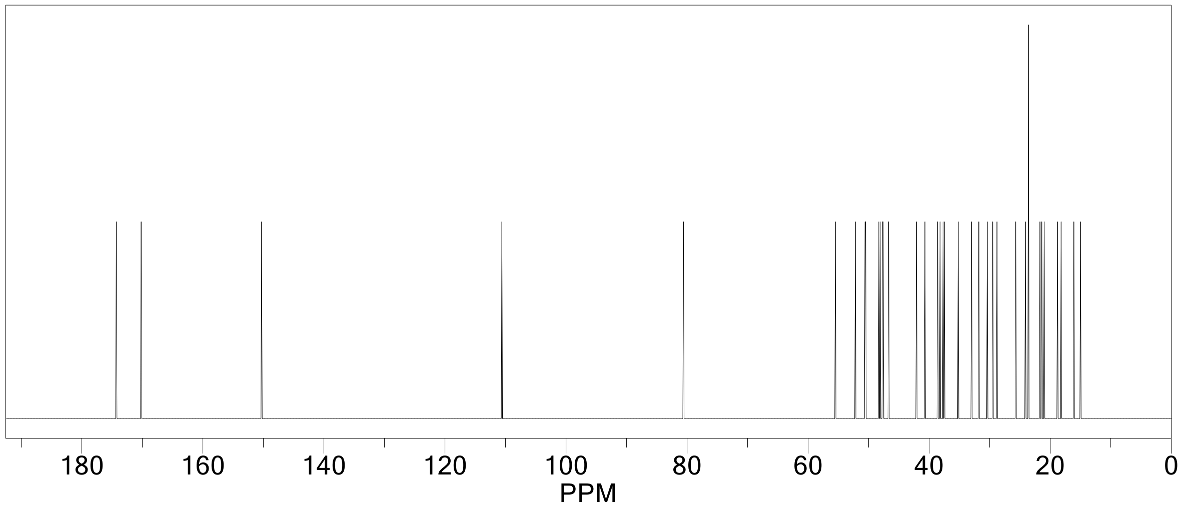

Figure S23. (3β)-28-Oxo-28-[*N*-(4-methoxybenzoyl)homopiperazin-1-yl]lup-20(29)-en-3-yl acetate (**13**): ^{1}H NMR spectrum measured (top) and calculated by ChemBioDraw Ultra, v. 12.0 (bottom)

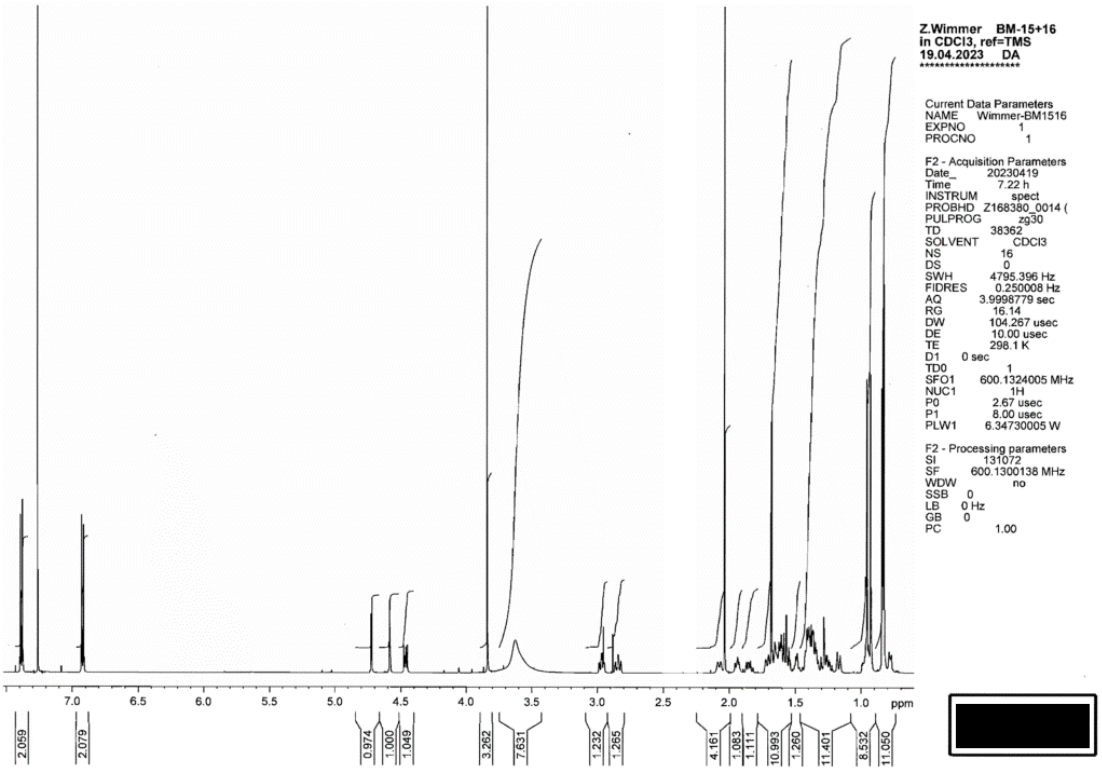

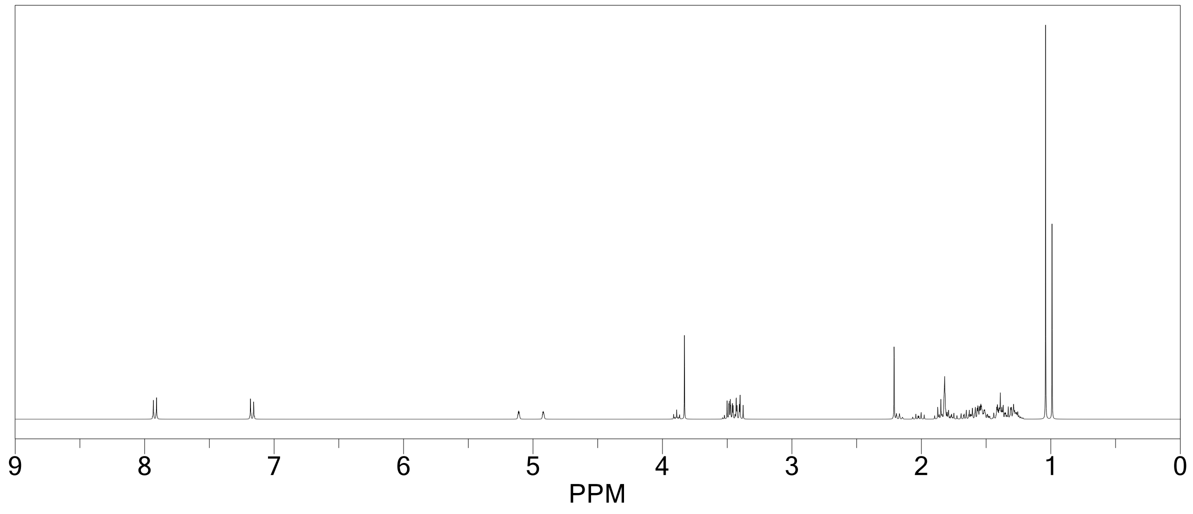

Figure S24. (3β)-28-Oxo-28-[*N*-(4-methoxybenzoyl)homopiperazin-1-yl]lup-20(29)-en-3-yl acetate (**13**): ^{13}C NMR spectrum measured (top) and calculated by ChemBioDraw Ultra, v. 12.0 (bottom)

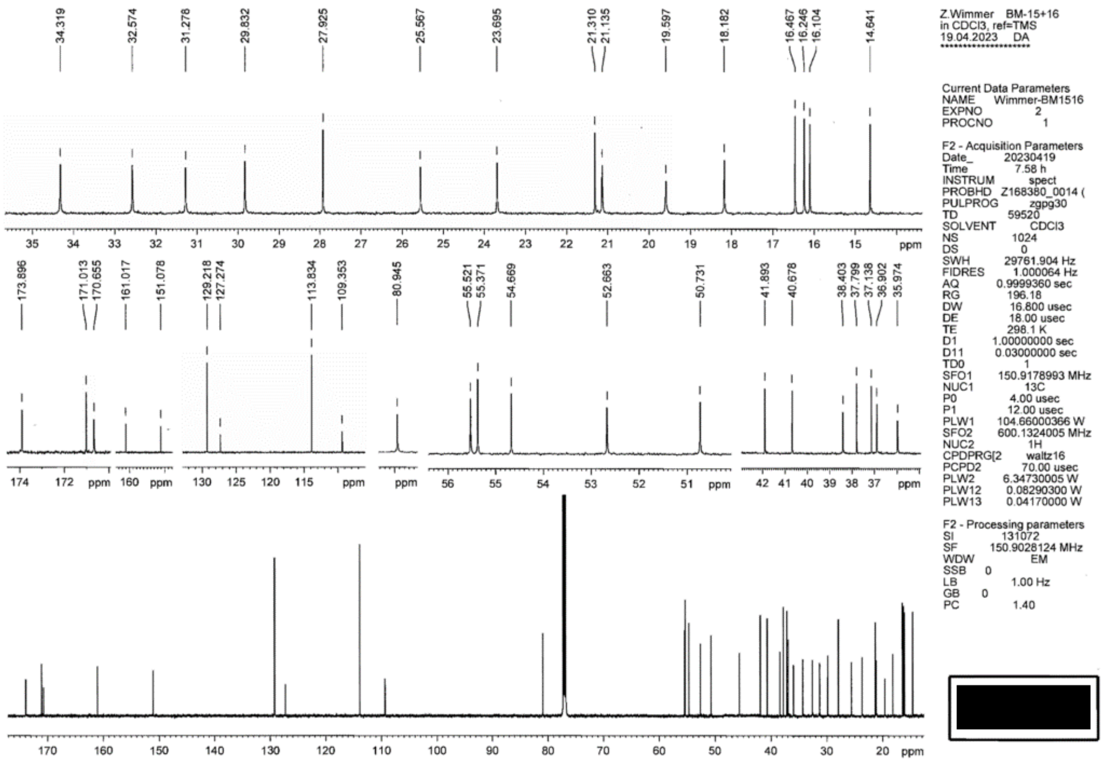

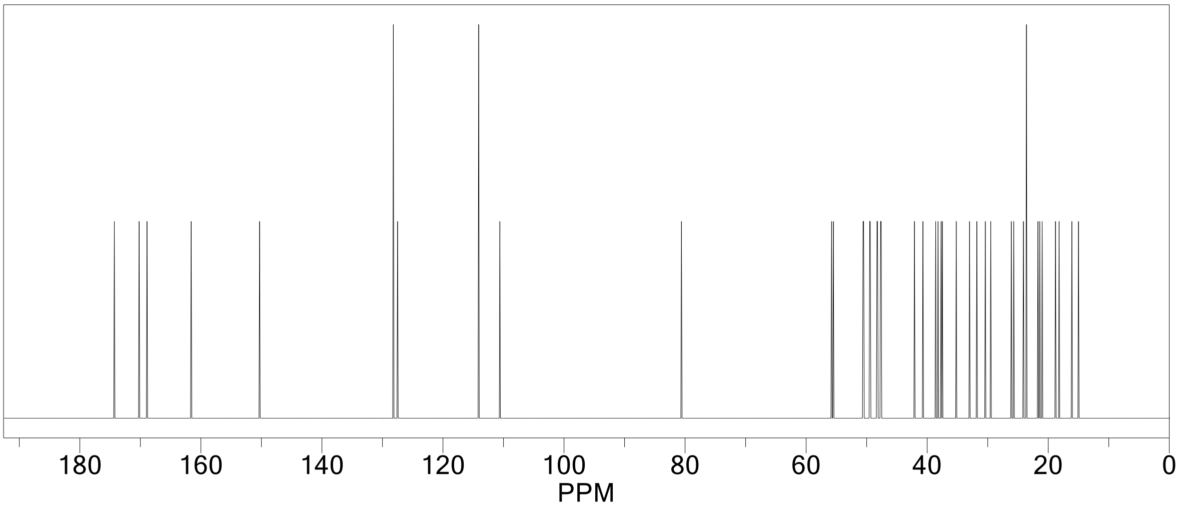

Figure S25. (3β)-3-Hydroxy-28-[*N*-(4-methoxybenzoyl)homopiperazin-1-yl]lup-20(29)-en-28-one (**14**): ^{1}H NMR spectrum measured (top) and calculated by ChemBioDraw Ultra, v. 12.0 (bottom)

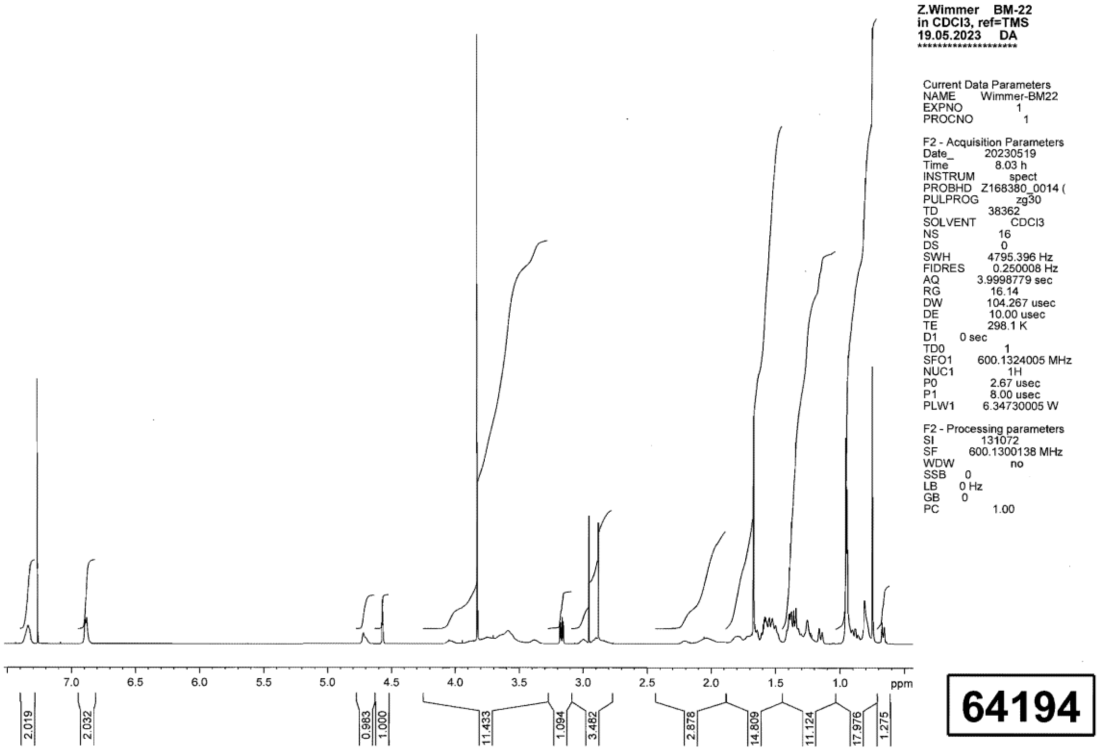

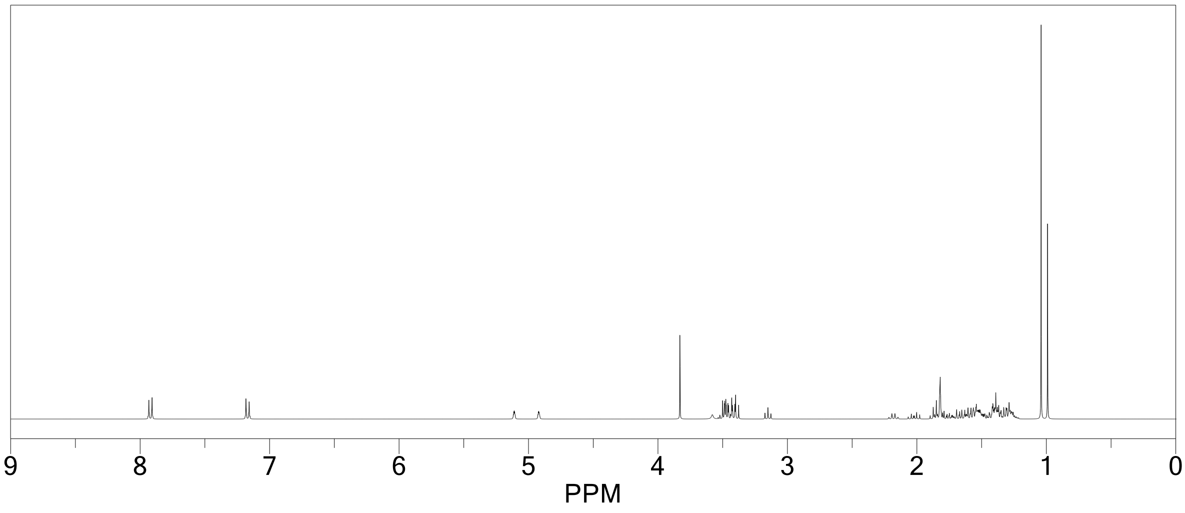

Figure S26. (3β)-3-Hydroxy-28-[*N*-(4-methoxybenzoyl)homopiperazin-1-yl]lup-20(29)-en-28-one (**14**): ^{13}C NMR spectrum measured (top) and calculated by ChemBioDraw Ultra, v. 12.0 (bottom)

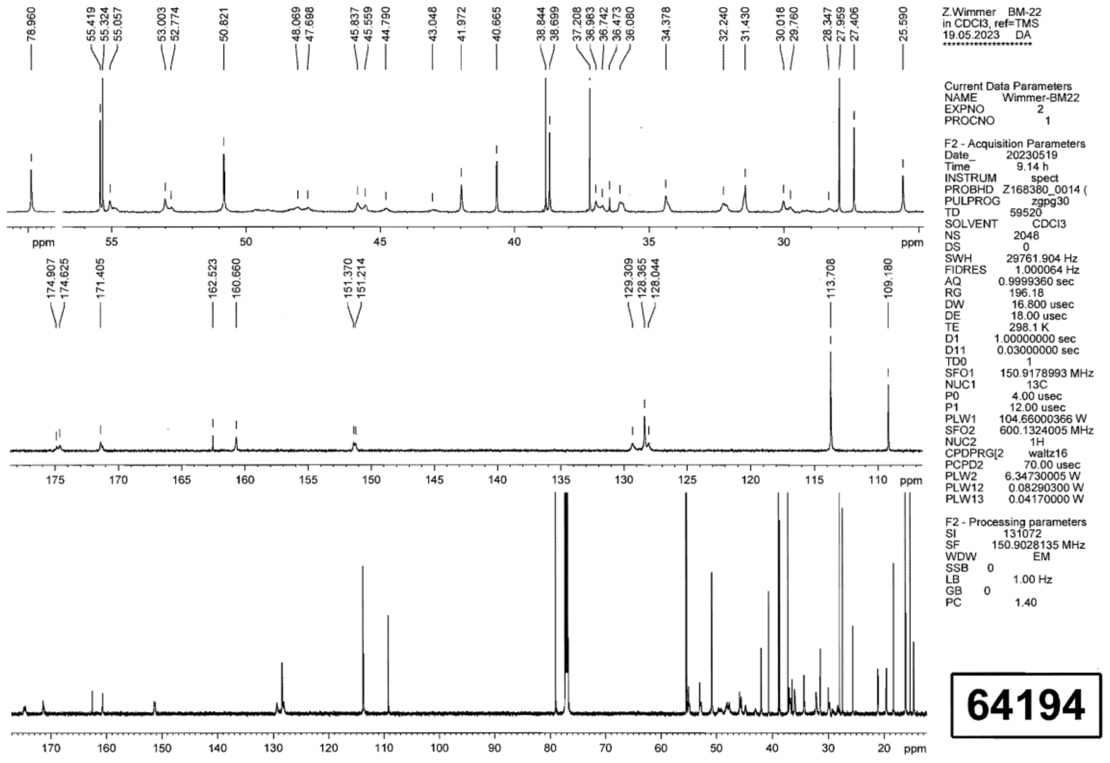

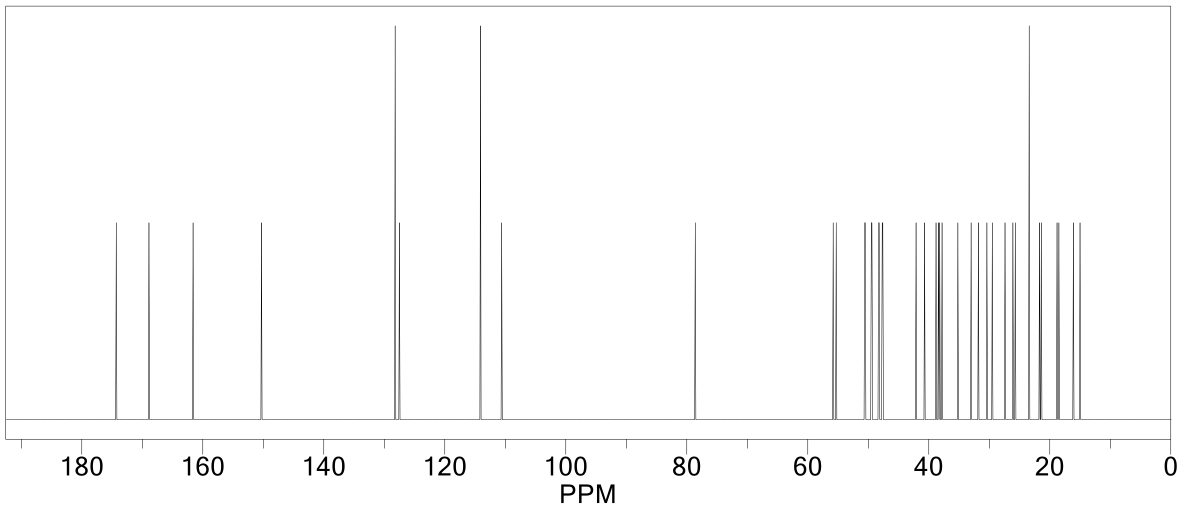

Figure S27. The 3D-structure of the compound **5**.

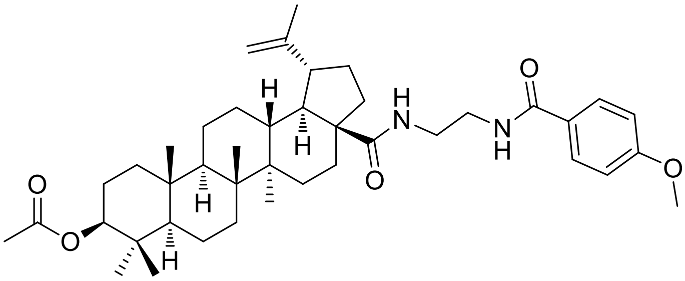

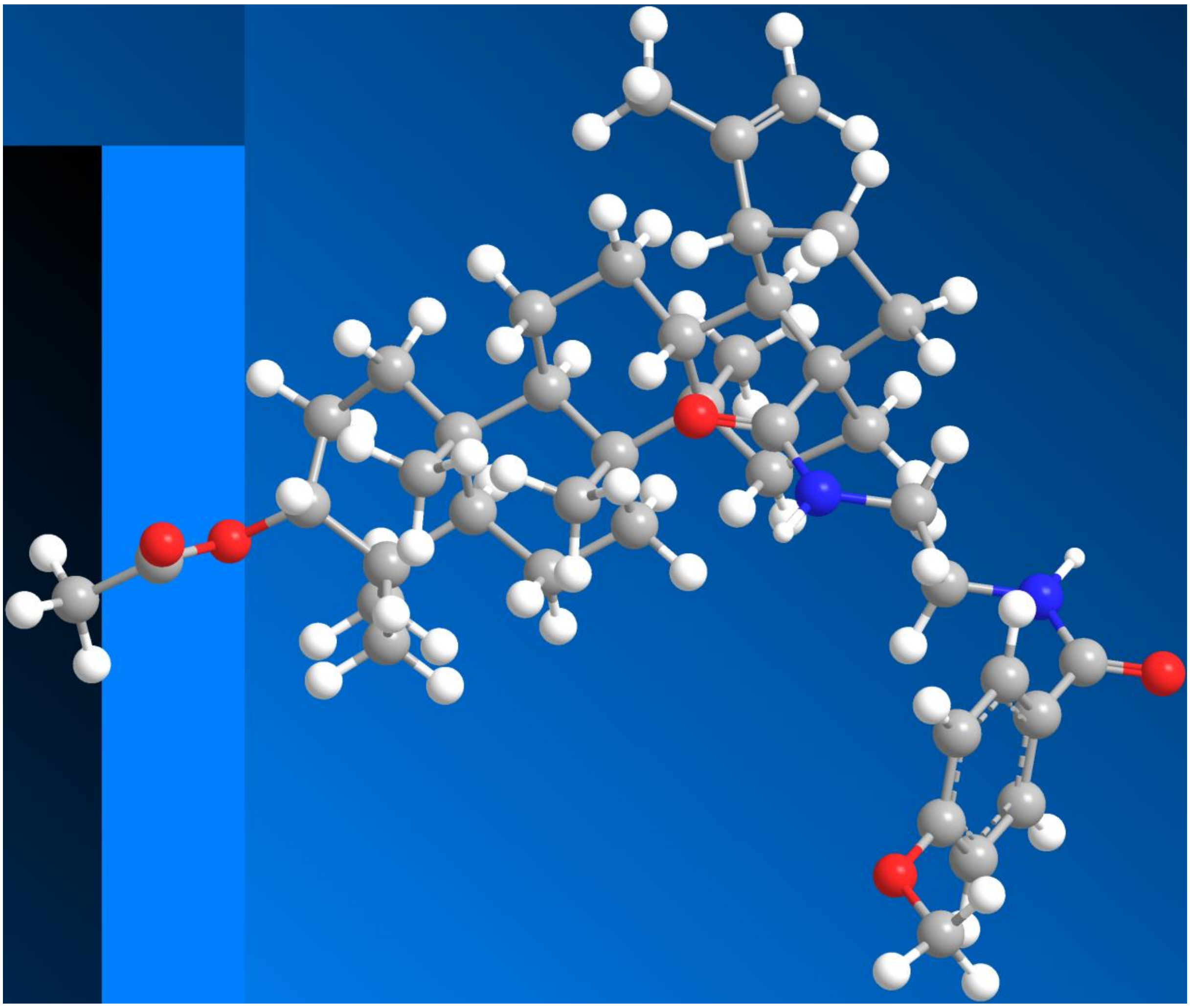

Figure S28. The 3D-structure of the compound **9**.

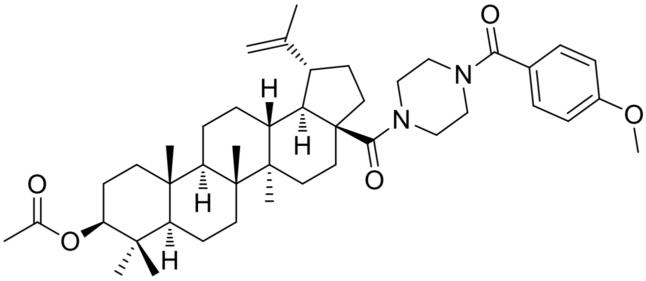

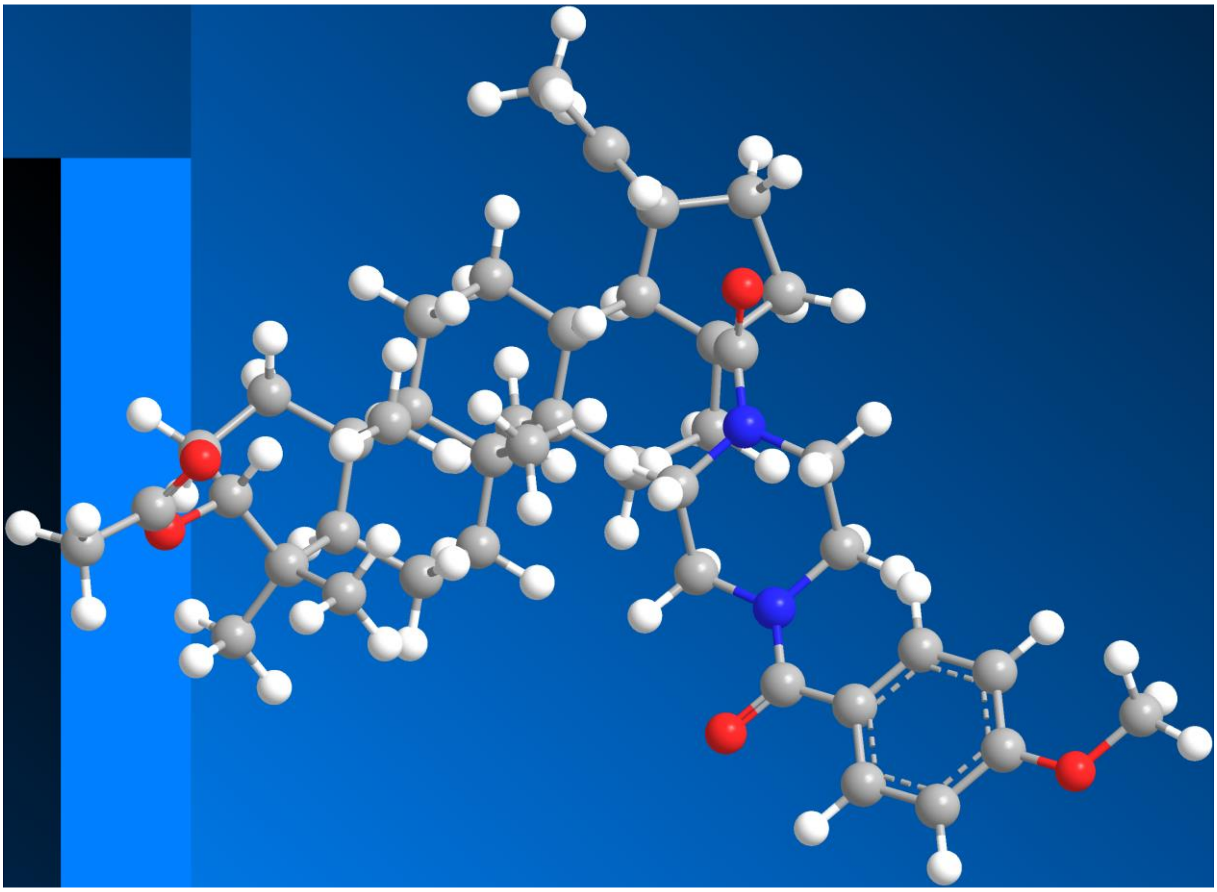

Figure S29. The 3D-structure of the compound **13**.

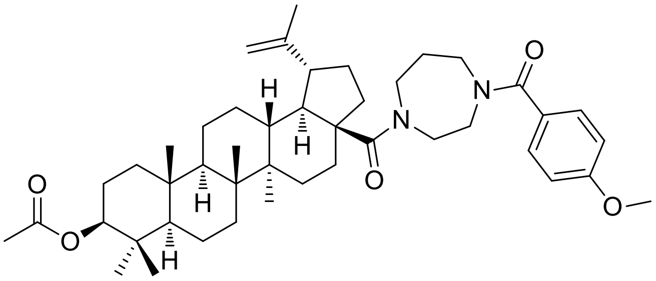

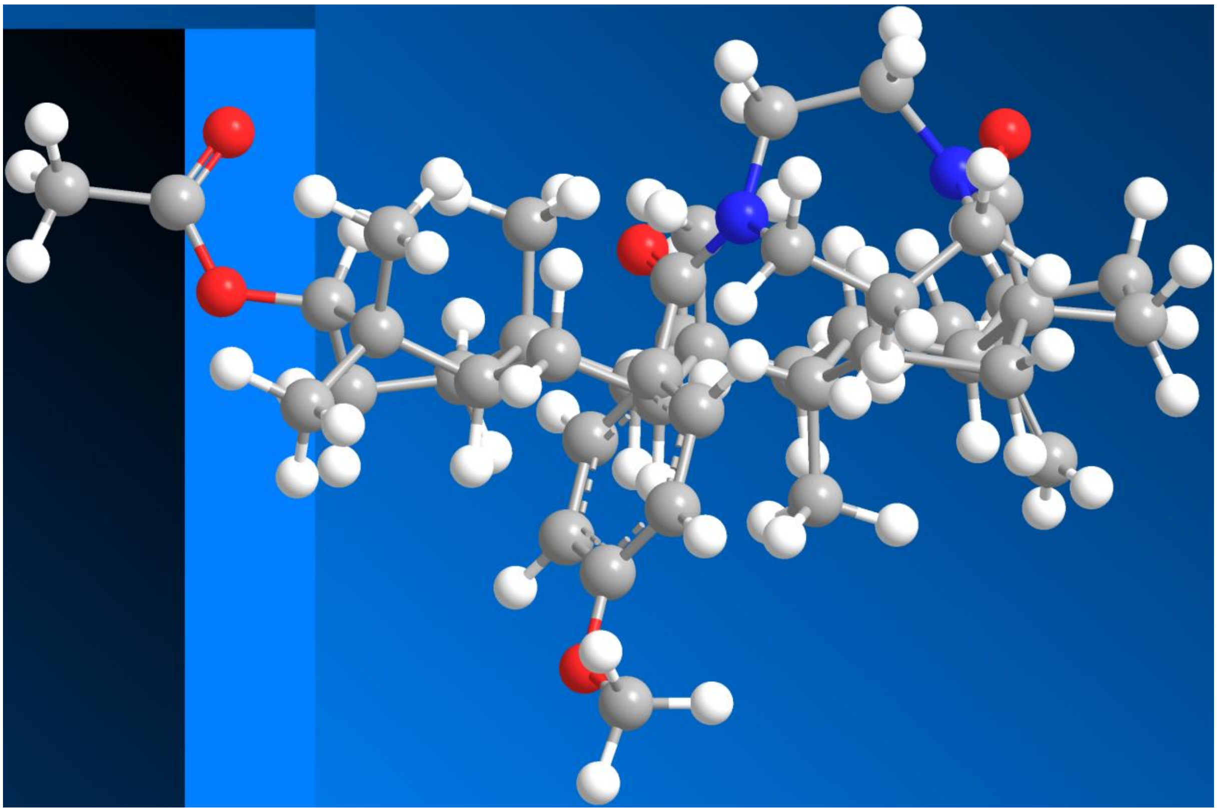

Part II. Results of the GC×GC analysis of the extracts of anise (*Pimpinella anisum* L.) seeds

The following pictures show the chromatogram of the mixture of compounds, a table of the identified compounds, and their structures mass spectra and the library hits.

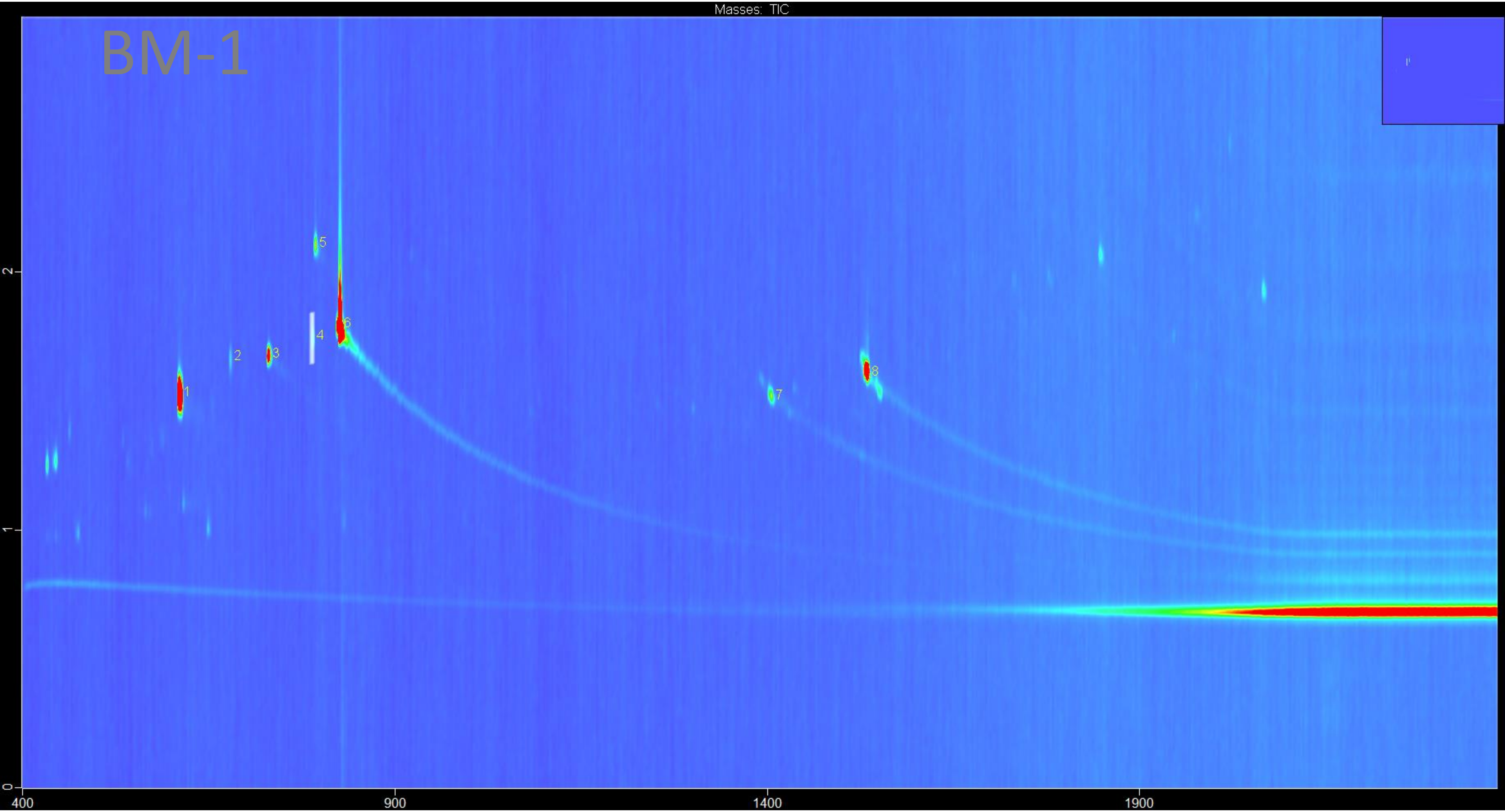
Masses: TIC
BM-1
1
2
3
4
5
6
7
8
0
1
2
400
900
1400
1900

Peak #	*Name*	*R.T. (s)*	*1st Dimension Time (s)*	*2nd Dimension Time (s)*	*UniqueMass*
1	Fenchone	610 , 1.533	610	1.533	81
2	Camphor	679 , 1.667	679	1.667	95
3	Estragole	730 , 1.678	730	1.678	148
4		790 , 1.745	790	1.745	103
5	Anisaldehyde	793 , 2.107	793	2.107	135
6	Anethole	826 , 1.802	826	1.802	119
7	Hexadecanoic acid	1405 , 1.519	1405	1.519	60
8	Oleic acid	1534 , 1.613	1534	1.613	141

Hit	Name	Similarity	Reverse	Probability	CAS	Library	Id	Formula	Weight	Contributor
1	Fenchone	944	953	4833	1195-79-5	mainlib	62373	C10H16O	152	NIST Mass Spectrometry Data Center, 1990.

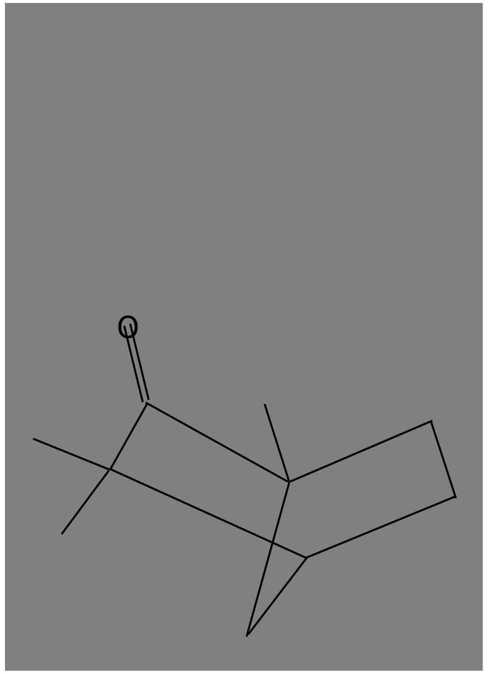

Peak True - sample "Library Search", peak 1, at 0 s (Spec # 0)

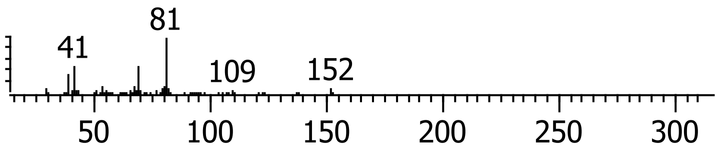

Peak True - sample "Library Search", peak 1, at 0 s (Spec # 0)
vs. Library Hit - similarity 944, "Fenchone"

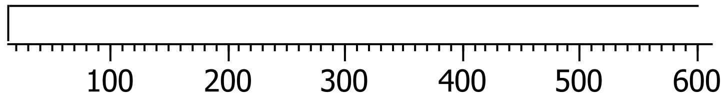

Library Hit - similarity 944, "Fenchone"

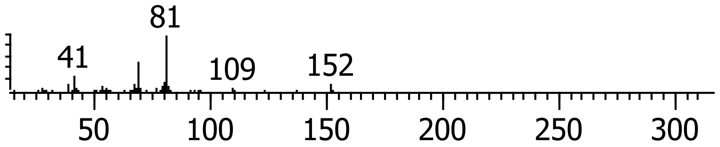

Hit	Name	Similarity	Reverse	Probability	CAS	Library	Id	Formula	Weight	Contributor
1	(+)-Camphor	916	930	7444	464-49-3	replib	17950	C10H16O	152	RADIAN CORP

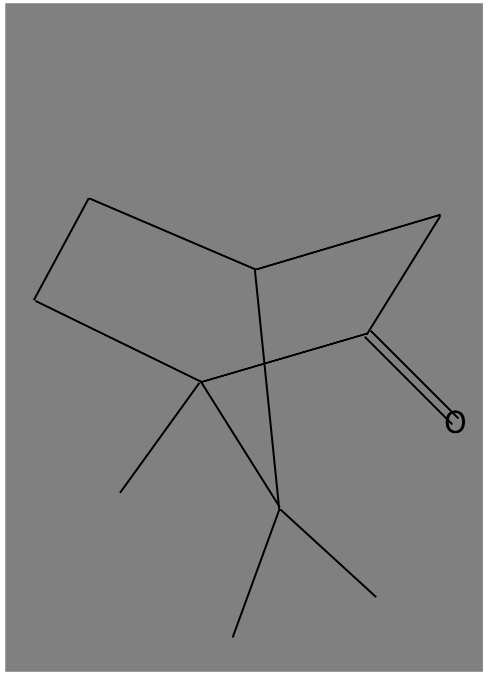

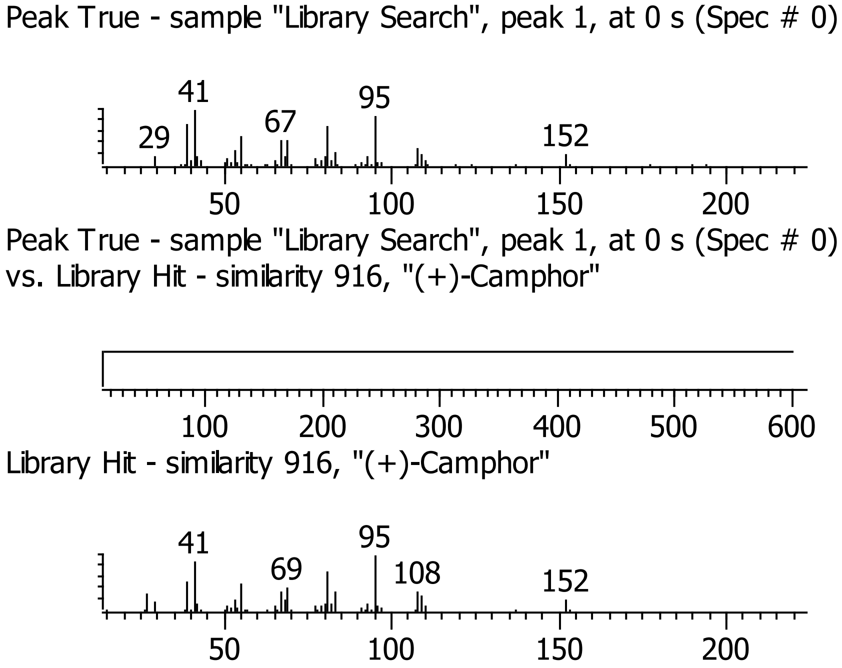

Hit	Name	Similarity	Reverse	Probability	CAS	Library	Id	Formula	Weight	Contributor
1	Estragole	939	947	7005	140-67-0	replib	29607	C10H12O	148	Chemical Concepts

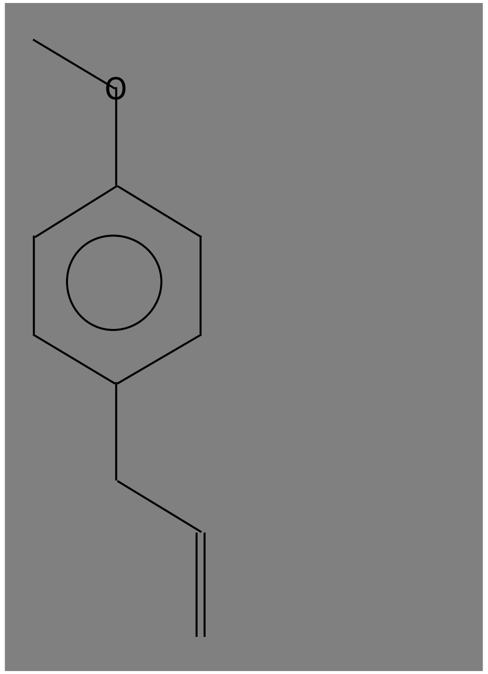

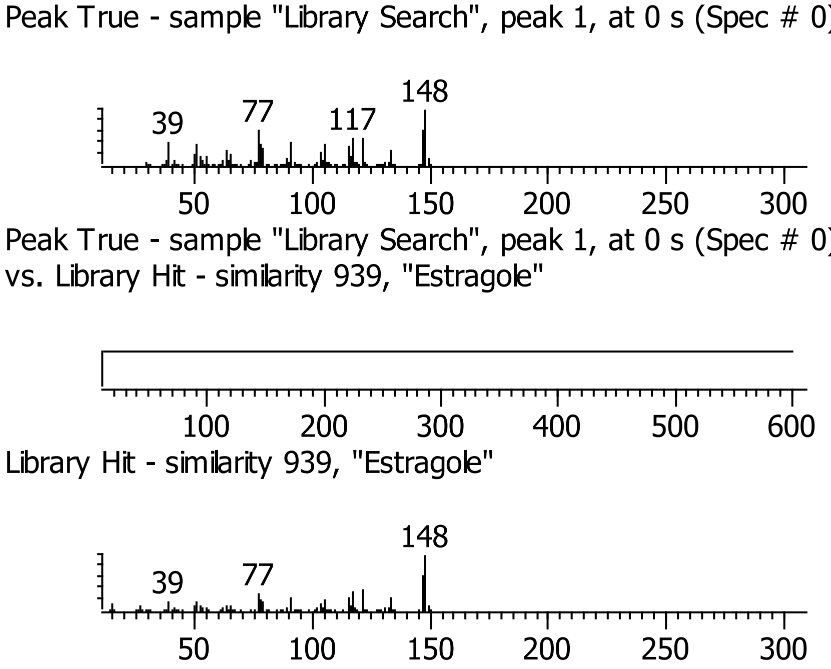

Hit	Name	Similarity	Reverse	Probability	CAS	Library	Id	Formula	Weight	Contributor	
1	Benzaldehyde, 4-methoxy-		925	928	7711	123-11-5	replib	27168	C8H8O2	136	V.A.Korolev, IOC RAS, Moscow, Russia

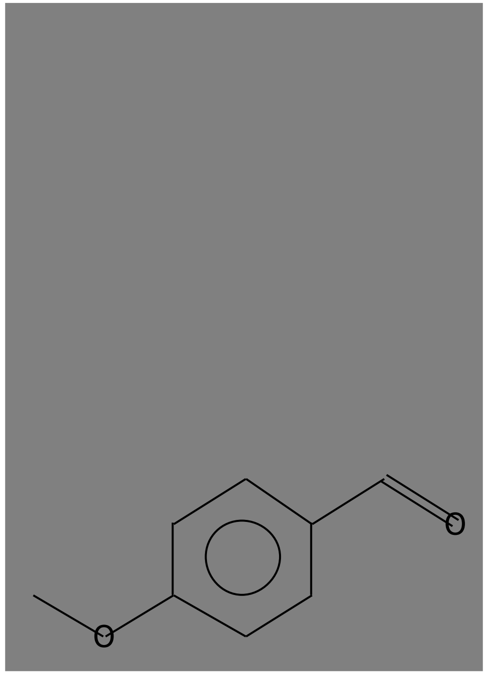

Peak True - sample "Library Search", peak 1, at 0 s (Spec # 0)

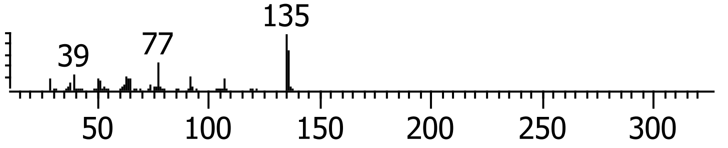

Peak True - sample "Library Search", peak 1, at 0 s (Spec # 0)
vs. Library Hit - similarity 925, "Benzaldehyde, 4-methoxy-"

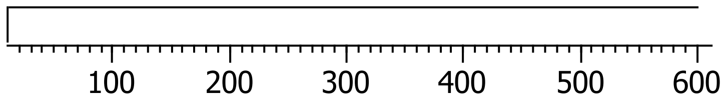

Library Hit - similarity 925, "Benzaldehyde, 4-methoxy-"

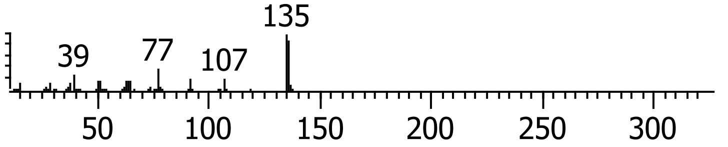

Hit	Name	Similarity	Reverse	Probability	CAS	Library	Id	Formula	Weight	Contributor
1	Anethole	933	935	3418	4180-23-8	mainlib	169700	C10H12O	148	Drug Lab

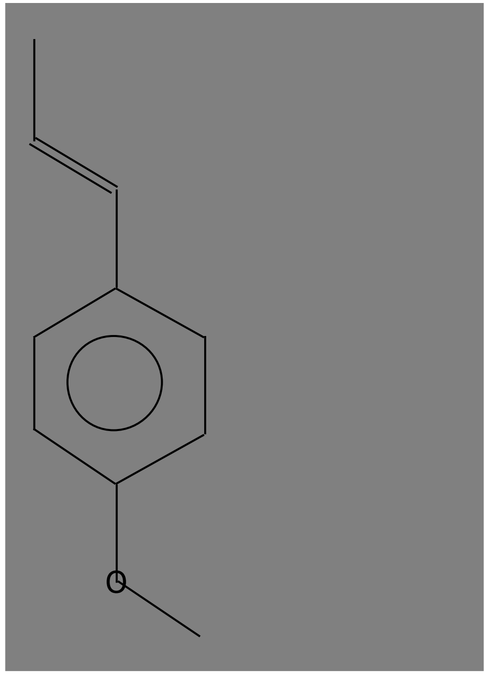

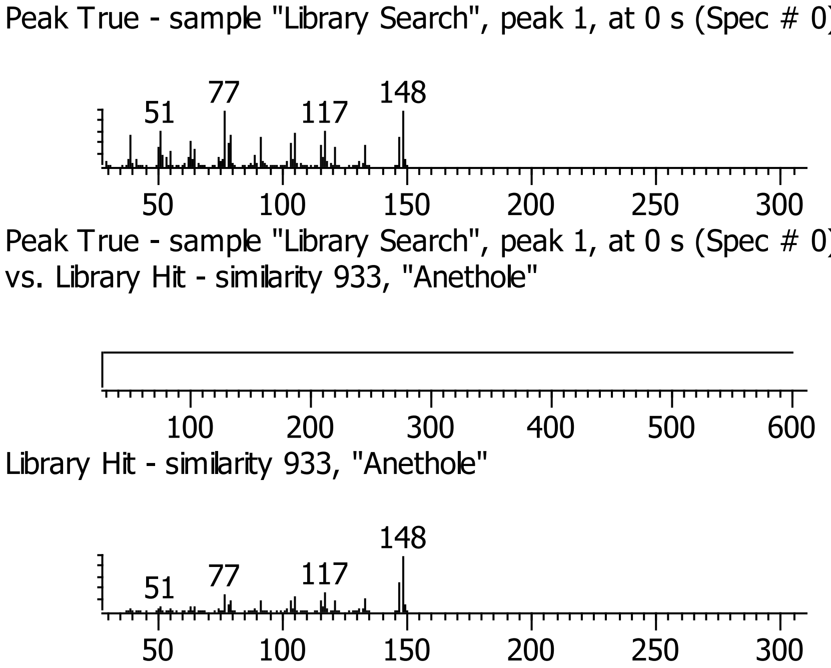

Hit	Name	Similarity	Reverse	Probability	CAS	Library	Id	Formula	Weight	Contributor
1	n-Hexadecanoic acid	894	897	6095	57-10-3	mainlib	10308	C16H32O2	256	Chemical Concepts

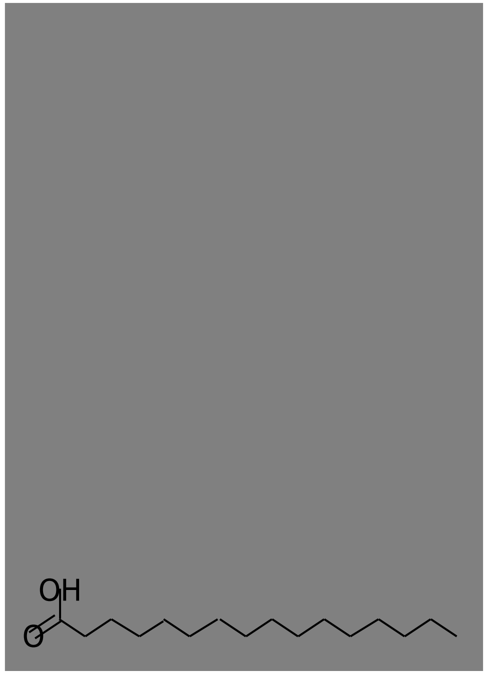

Peak True - sample "Library Search", peak 1, at 0 s (Spec # 0)

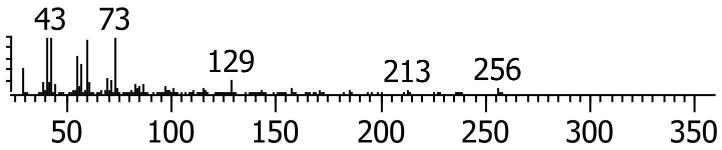

Peak True - sample "Library Search", peak 1, at 0 s (Spec # 0)
vs. Library Hit - similarity 894, "n-Hexadecanoic acid"

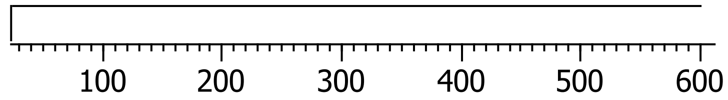

Library Hit - similarity 894, "n-Hexadecanoic acid"

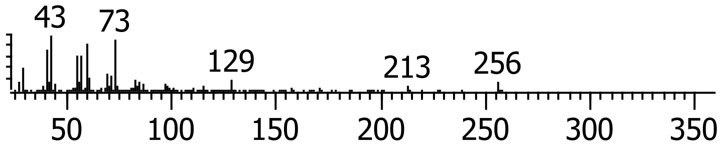

Hit	Name	Similarity	Reverse	Probability	CAS	Library	Id	Formula	Weight	Contributor
1	Oleic Acid	920	928	2052	112-80-1	mainlib	3111	C18H34O2	282	NIST Mass Spectrometry Data Center, 1994

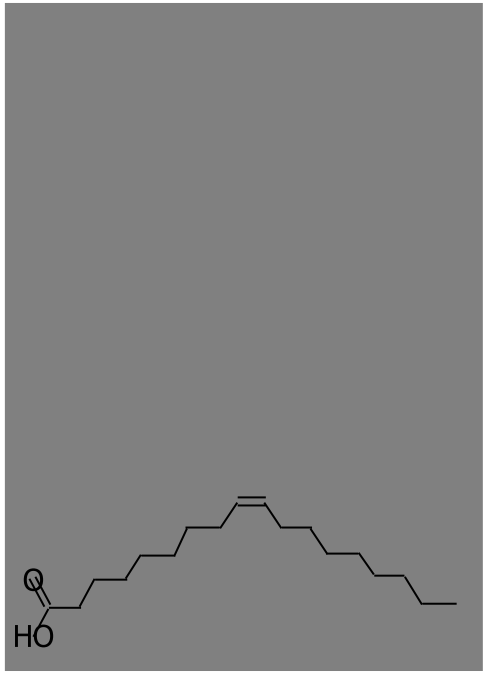

Peak True - sample "Library Search", peak 1, at 0 s (Spec # 0)

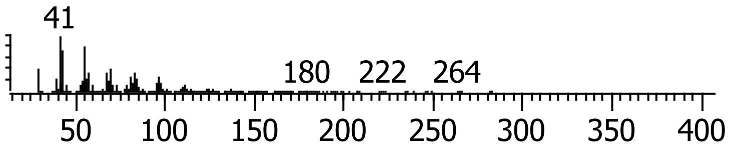

Peak True - sample "Library Search", peak 1, at 0 s (Spec # 0)
vs. Library Hit - similarity 920, "Oleic Acid"

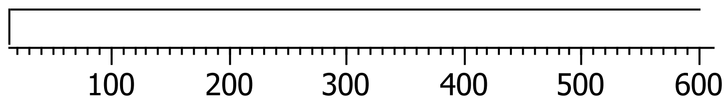

Library Hit - similarity 920, "Oleic Acid"

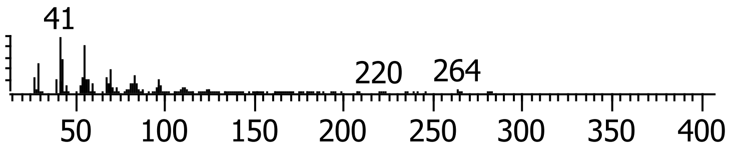

Part III. The 2D $^{1}H,^{13}C$ gHSQC and gHMBC NMR spectra.

The 2D NMR spectroscopy enables structure elucidation by spreading signals over two frequency axes, resolving overlapping peaks and revealing correlations between nuclei, such as homonuclear $^{1}H,^{1}H$ and heteronuclear $^{1}H,^{13}C$ interactions. As mentioned already in the main text, the 2D $^{1}H,^{13}C$ gHSQC and gHMBC NMR spectra were recorded and evaluated. The scanned 2D $^{1}H,^{13}C$ gHSQC NMR spectra clearly show the interactions correlating a hydrogen atom with the directly bonded carbon atom, $^{1}H,^{13}C$ gHMBC then confirm the correct structure assingment through the H/C interaction over two or three bonds. Most of the 2D NMR spectra shown in Figure S30–S37 are the 2D $^{1}H,^{13}C$ gHSQC NMR spectra giving the proofs of the correct structure elucidation. The 2D $^{1}H,^{13}C$ gHMBC NMR spectra were recorded for the only selected compounds to make additional proofs of the correctness of the structure elucidations. The 2D $^{1}H,^{13}C$ gHSQC and gHMBC NMR spectra are very illustrative and need no more explanations because this NMR technique is the commonly used one in the current structure elucidation practice.

Figure S30. The 2D $^{1}H,^{13}C$ gHSQC NMR spectrum of **3**

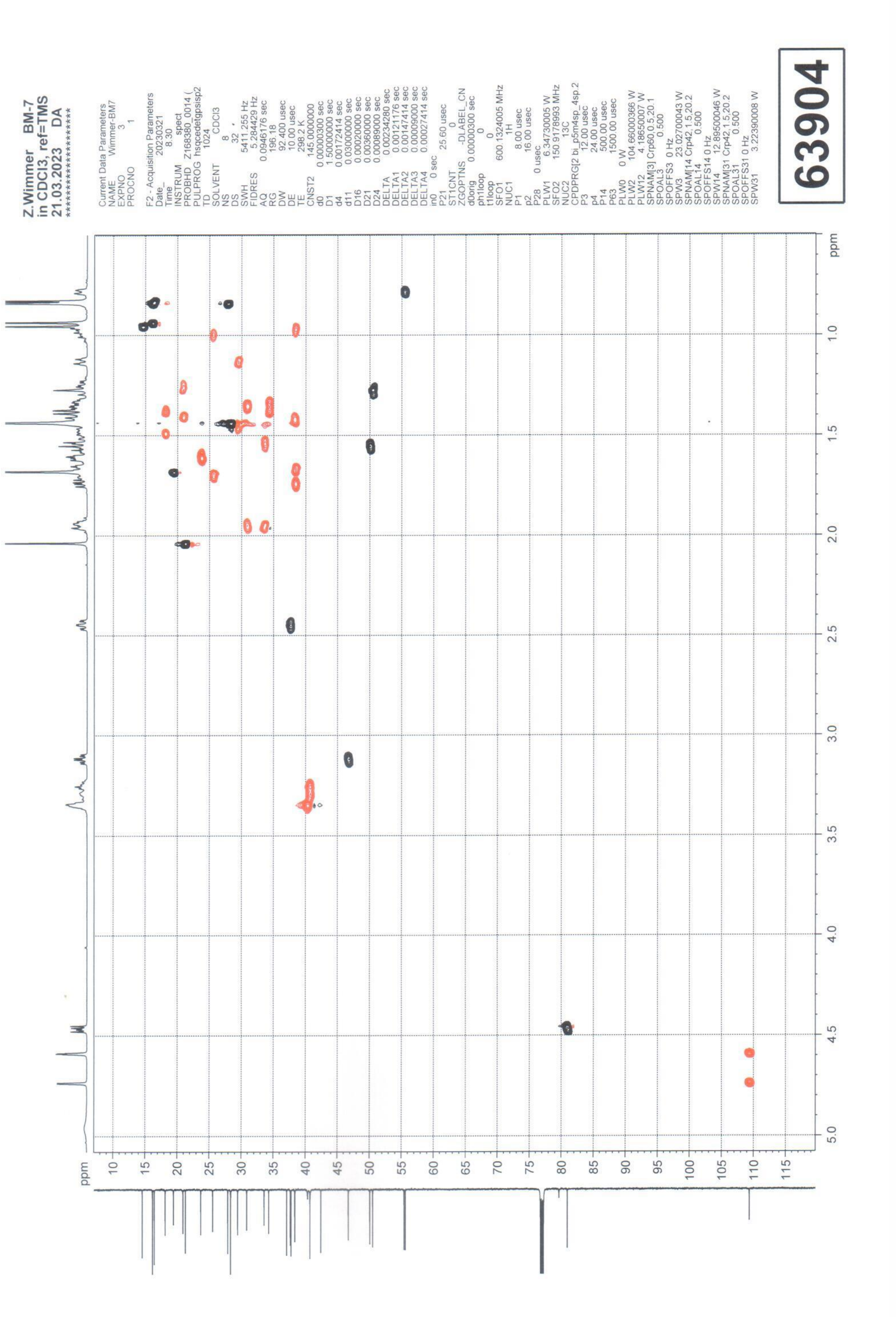

Figure S31. The 2D $^{1}H,^{13}C$ gHSQC NMR spectrum of **5**

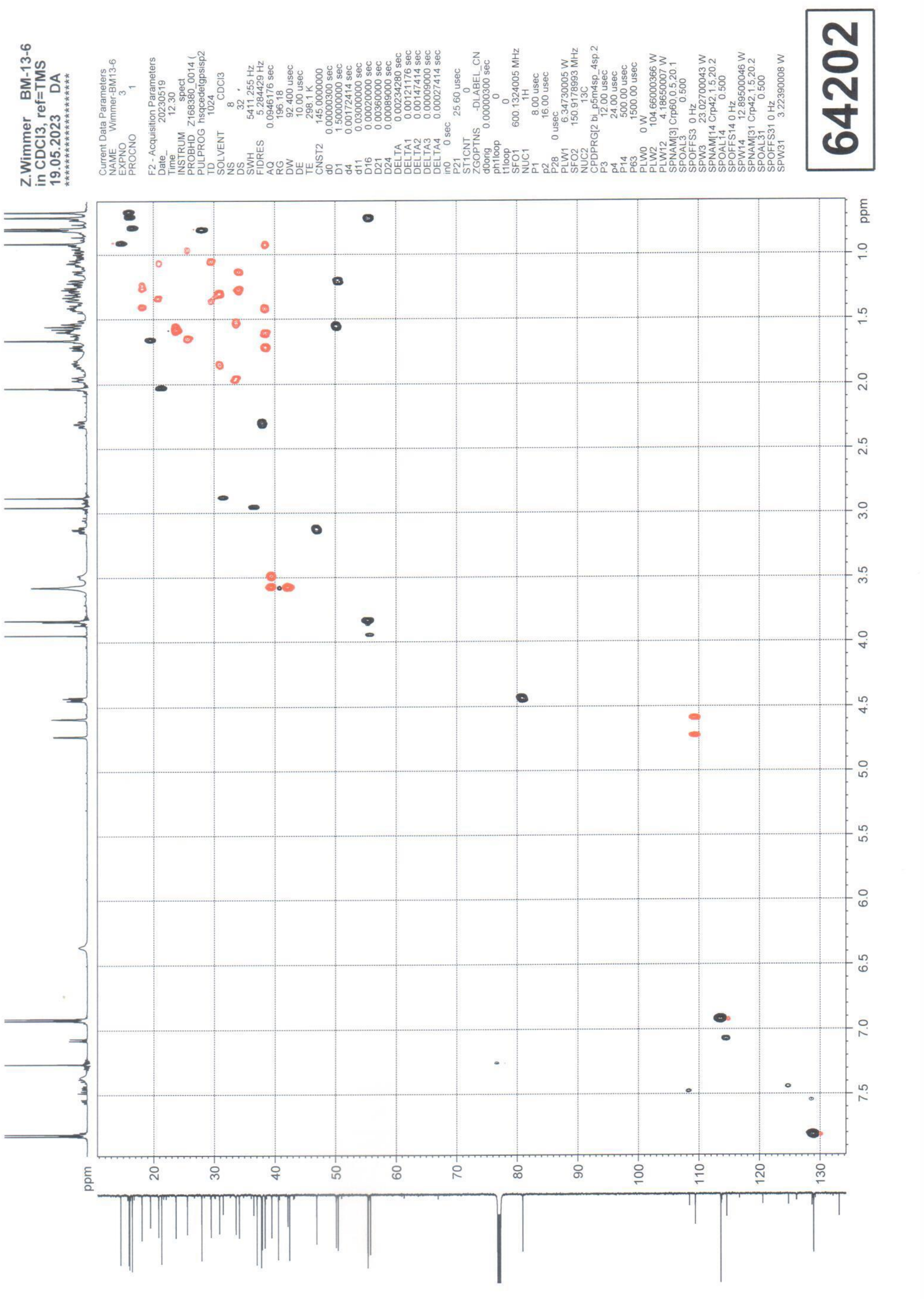

Figure S32. The 2D $^{1}H,^{13}C$ gHSQC NMR spectrum of **6**

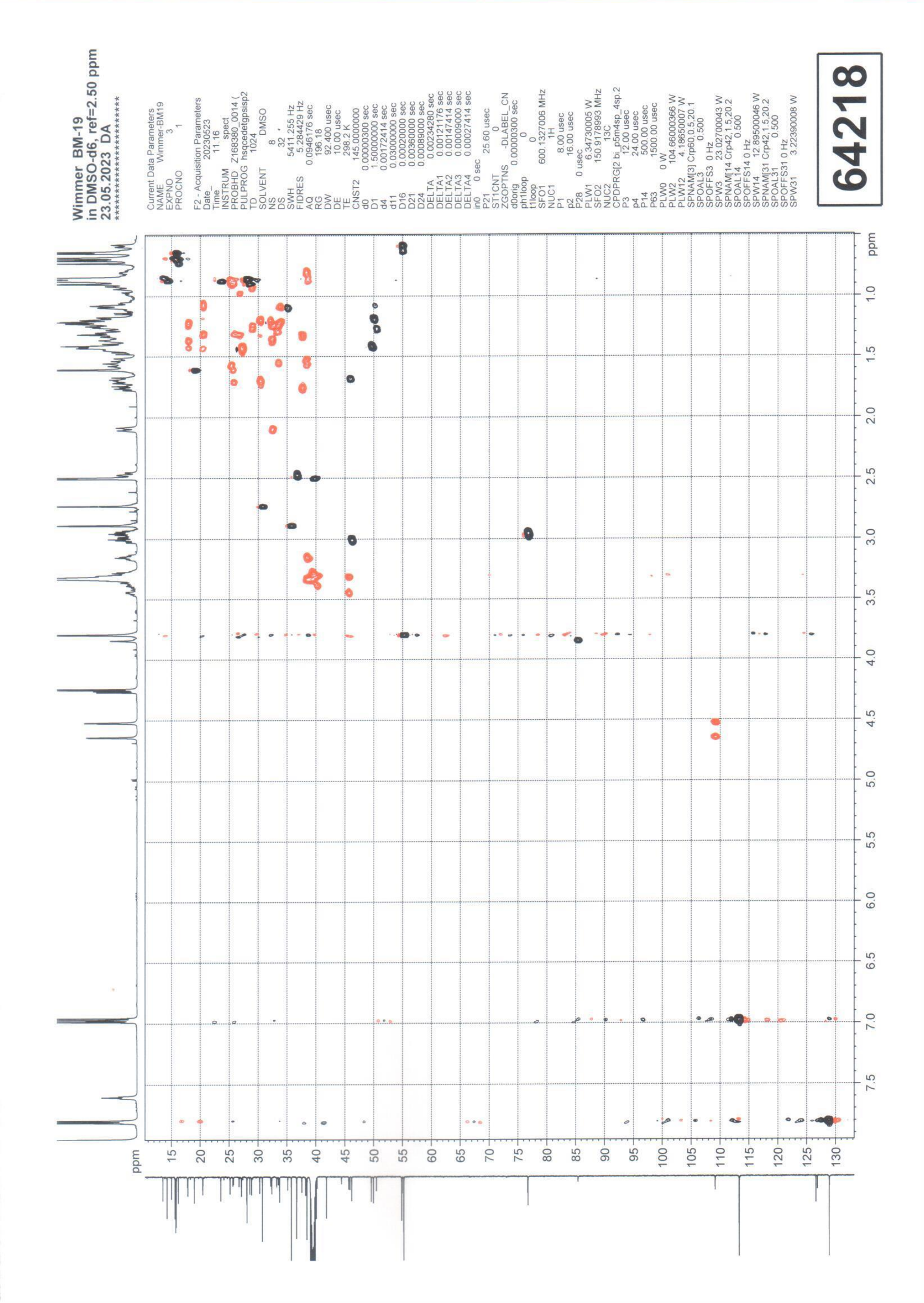

Figure S33. The 2D $^{1}H,^{13}C$ gHSQC NMR spectrum of **8**

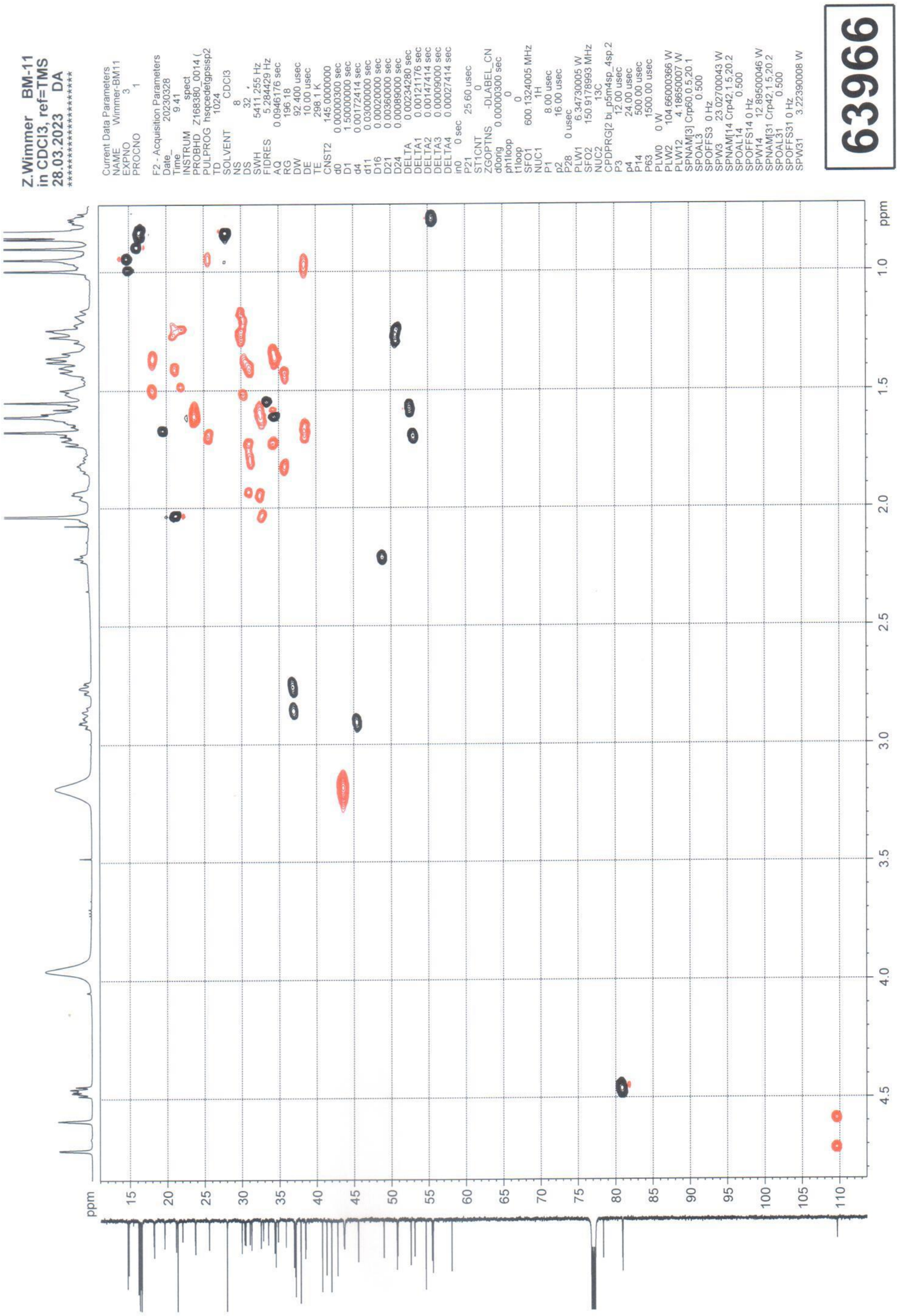

Figure S34. The 2D ^{1}H,^{13}C gHSQC NMR spectrum of **9**

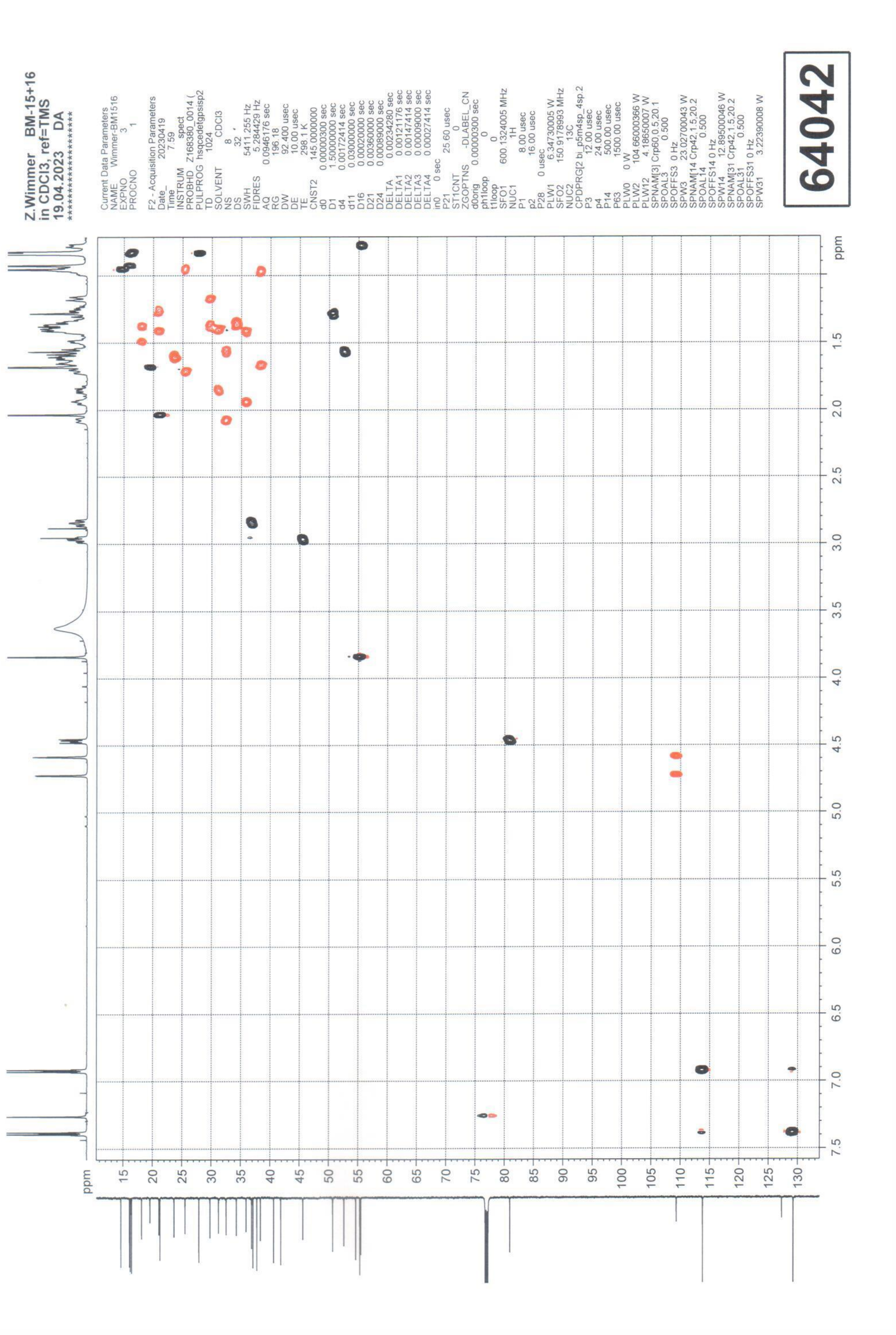

Figure S35. The 2D $^{1}H,^{13}C$ gHMBC NMR spectrum of **9**

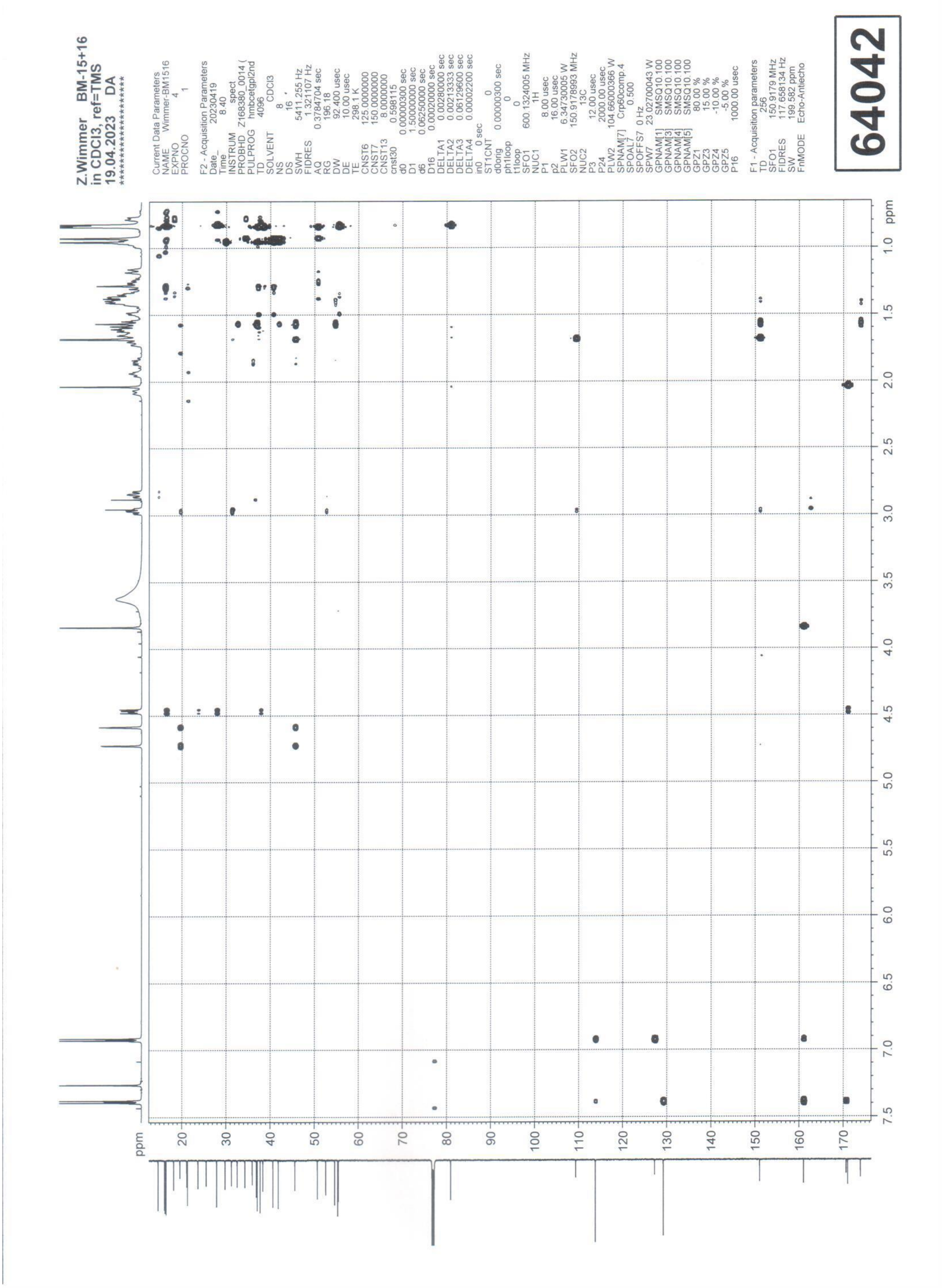

Figure S36. The 2D $^{1}H,^{13}C$ gHSQC NMR spectrum of **10**

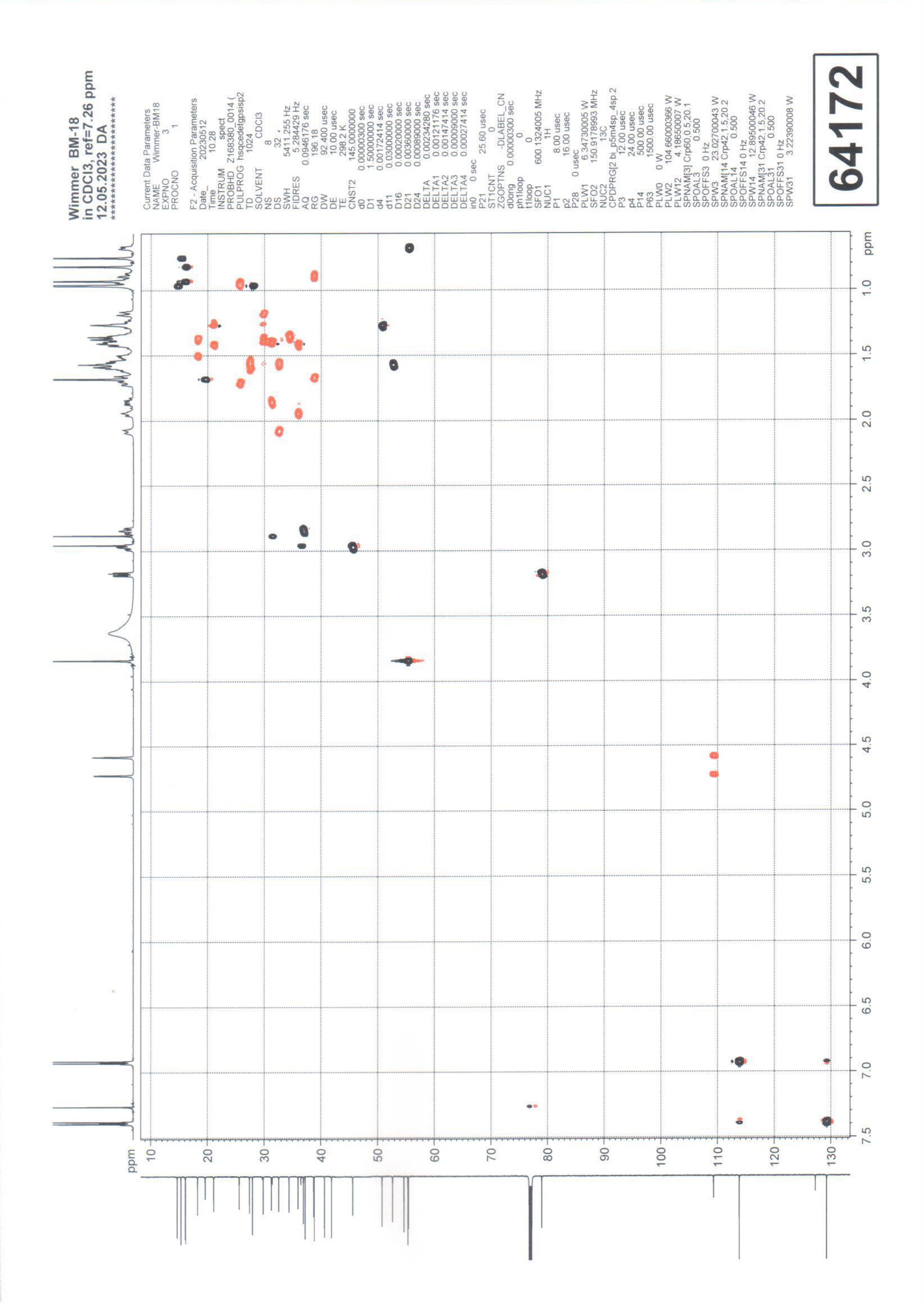

Figure S37. The 2D ^{1}H,^{13}C gHSQC NMR spectrum of **14**

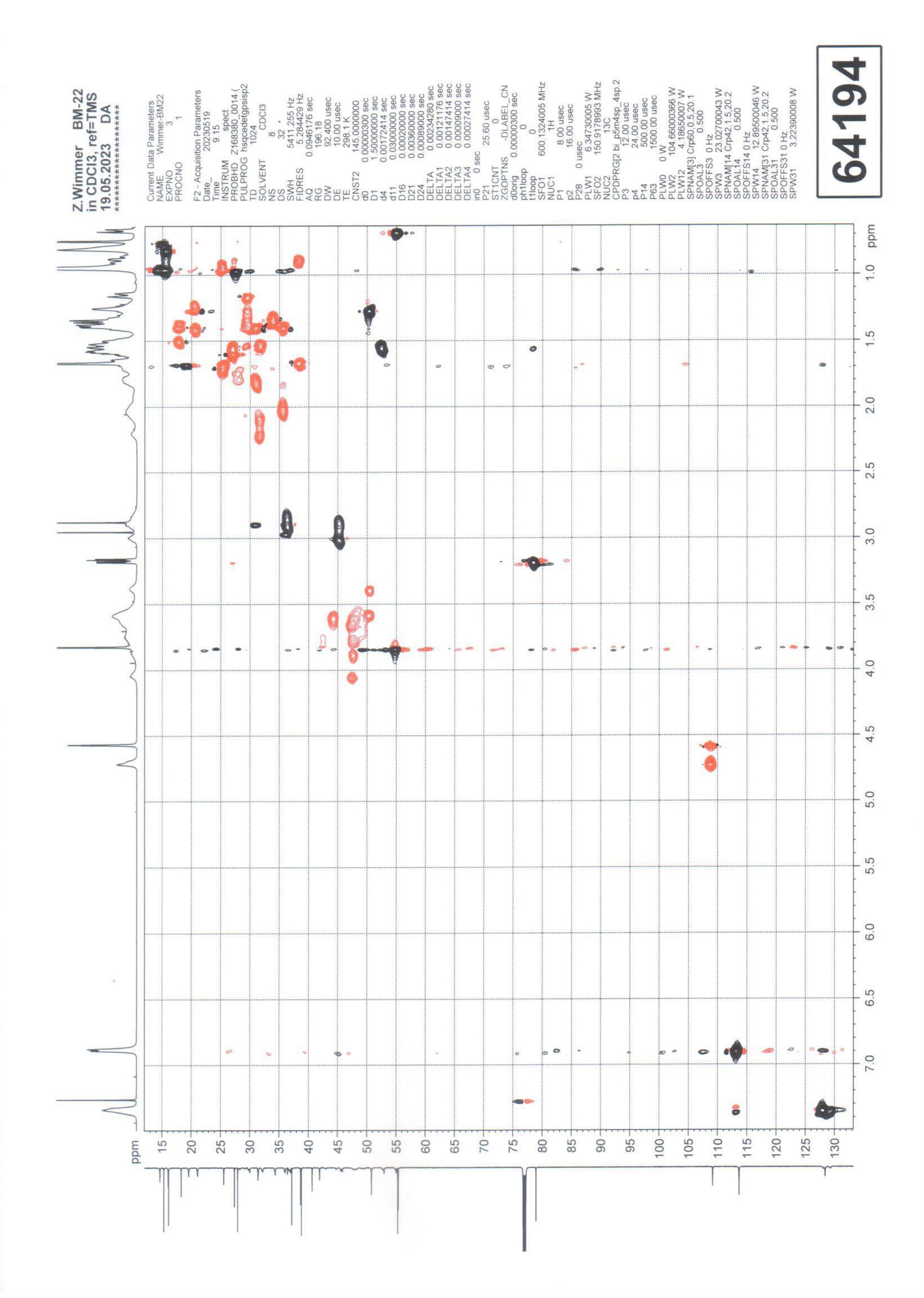

Supplement: Supplementary file 1 [file ao6c00184_si_001.pdf]
